# Real-world effectiveness and safety of tonic motor activation therapy for refractory restless legs syndrome: 90-day outcomes from the THRIVE prospective observational study

**DOI:** 10.1093/sleep/zsag140

**Published:** 2026-06-03

**Authors:** Haramandeep Singh, Mark J Buchfuhrer, Joseph Ojile, Asim Roy, Robert Joseph Thomas, Richard K Bogan, Avinash Aggarwal, Jessica Preciado, Kurtis J Swartz, Jonathan D Charlesworth

**Affiliations:** iSleep Physicians, San Ramon, CA, United States; Stanford University School of Medicine, Stanford, CA, United States; Clayton Sleep Institute, LLC, St. Louis, MO, United States; Ohio Sleep Medicine Institute, Dublin, OH, United States; Department of Medicine, Division of Pulmonary, Critical Care and Sleep Medicine, Beth Israel Deaconess Medical Center, Boston, MA, United States; Bogan Sleep Consultants, LLC, Columbia, SC, United States; University of Pittsburgh Medical Center, Department of Neurology, Pittsburgh, PA, United States; Noctrix Health, Inc., Pleasanton, CA, United States; Noctrix Health, Inc., Pleasanton, CA, United States; Noctrix Health, Inc., Pleasanton, CA, United States

**Keywords:** restless legs syndrome, peripheral nerve stimulation, tonic motor activation, neurological disorder, sleep disorder, neuromodulation, neurostimulation

## Abstract

**Study Objectives:**

The purpose of the THRIVE study is to evaluate the outcomes of patients receiving tonic motor activation (TOMAC) for treatment of refractory restless legs syndrome (RLS) in real-world clinical practice. This article examines outcomes after 90 days of TOMAC treatment.

**Methods:**

THRIVE is a multicenter, prospective, observational study that enrolled 325 patients prescribed TOMAC (Nidra) for the treatment of RLS. The indicated intent-to-treat analysis population included adults with refractory moderate–severe RLS. The primary endpoint was change in International RLS Study Group (IRLS) score. Secondary endpoints included Clinician Global Impressions-Improvement score and change in Medical Outcomes Study Sleep Problems Index II (MOS-II) score. Adjunctive RLS medications were assessed at study entry and day 90.

**Results:**

A total of 232 patients met criteria for indicated intent-to-treat analysis. Change in IRLS score at day 90 was −8.4 (95% CI: −7.4 to −9.4). Clinician Global Impressions-Improvement responder rate was 64% and mean score was 2.4 (95% CI: 2.2 to 2.6). Change in MOS-II score was −12.8 (95% CI −10.3, −15.3). Reductions to adjunctive RLS medication dosage were more common than increases (19% decreased vs. 11% increased; *p* = .01) and were well-tolerated (mean IRLS change: −8.1; 95% CI −6.3, −9.9). There were no statistically significant predictors of effectiveness. There were no severe or serious device-related adverse events.

**Conclusions:**

In real-world clinical practice, TOMAC is safe and effective at reducing RLS symptoms and improving sleep in adults with moderate-to-severe refractory RLS.

**Clinical Trial:**

A Post-Market Study for Long-Term Effectiveness and Safety of the Noctrix TOMAC System for RLS (THRIVE); https://clinicaltrials.gov/study/NCT06076499; Registered at ClinicalTrials.gov with the identifier number NCT06076499

**Statement of Significance**

This paper reports the 90-day results from the first real-world study to evaluate the safety and effectiveness of tonic motor activation (TOMAC) for treatment of refractory restless legs syndrome. We report that the effectiveness and safety of TOMAC is similar in real-world practice compared to controlled trials. This study had the largest sample size and the most heterogeneous patient population of TOMAC clinical trials, thereby allowing comparison of outcomes among different subgroups; we found that TOMAC is equally effective across subgroups. Furthermore, many patients were able to tolerate substantial reduction in dosage of dopamine agonists, which is of great interest given the shift away from dopamine agonists in clinical guidelines due to their risk for augmentation.

## Graphical Abstract

**Graphical Abstract:**
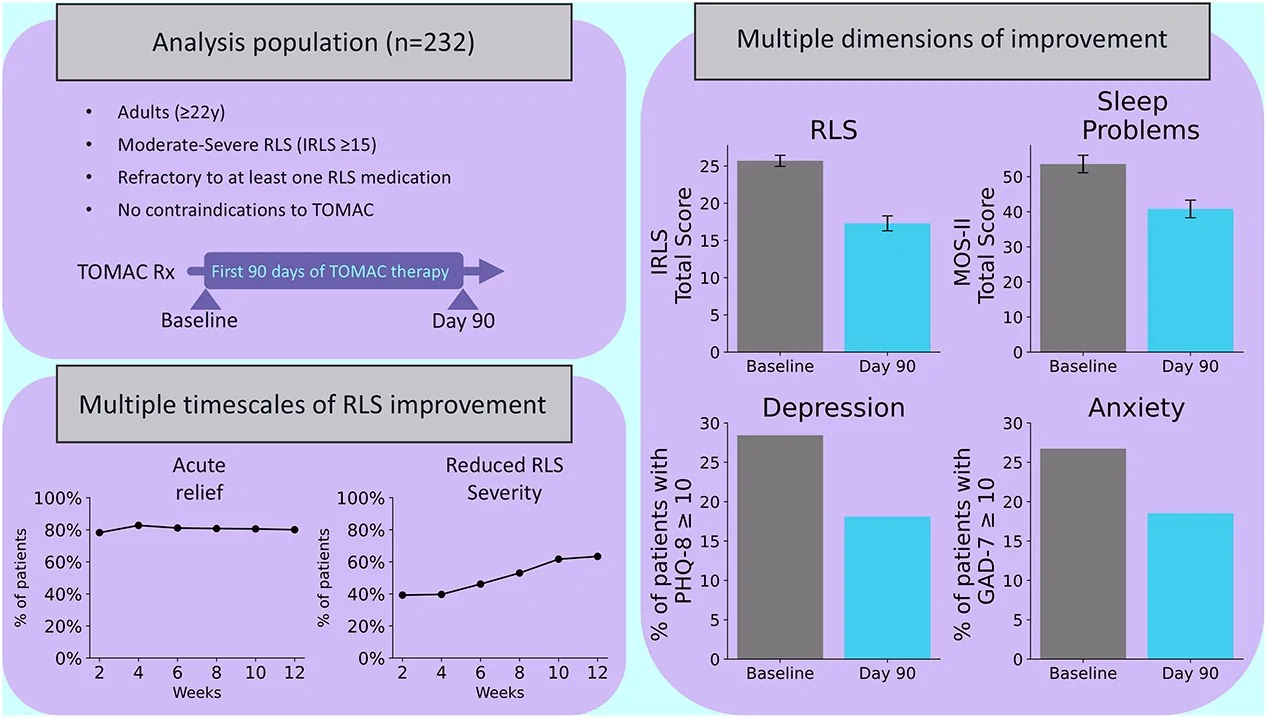

## Introduction

Restless legs syndrome (RLS) is a neurological disorder characterized by uncomfortable leg sensations and an irresistible urge to move the legs, usually late in the circadian day and most often experienced while resting or in bed [1]. Moderate-to-severe RLS, prevalent in 2%–3% of American and European adults [2], carries a significant clinical burden primarily due to its adverse effects on sleep resulting in sleep deprivation, excessive daytime sleepiness, and decreased daytime functioning [3].

With the recent shift away from standard use of dopamine agonists (DAs) for RLS treatment [4], there is a growing need for nonpharmacological treatment options for RLS. The one nonpharmacological treatment recommended in the 2025 AASM Clinical Practice Guidelines was bilateral high-frequency peroneal nerve stimulation, the generic terminology for tonic motor activation (TOMAC) [4]. TOMAC is externally worn and has a favorable safety profile [5] and plausible mechanism of action [6], but there is limited real-world clinical experience since TOMAC has only been available for prescription since August 2023.

The THRIVE study (see Materials and methods section for full name) is the first investigation of the safety and effectiveness of TOMAC in real-world clinical practice. THRIVE is a longitudinal, observational, prospective post-market study of participants prescribed TOMAC for the treatment of RLS. The prespecified primary effectiveness hypothesis was that the mean International RLS Study Group (IRLS) score will decrease after 90 days of TOMAC relative to baseline. Previously, three randomized controlled trials [7–9], and two open-label studies [10, 11] demostrated the efficacy and safety of TOMAC in adults with moderate-to-severe RLS. THRIVE employs an all-comers design to evaluate response to TOMAC in a more heterogeneous patient population, which is representative of real-world clinical practice and has the potential to identify potential predictors of response. Moreover, THRIVE employs a purely observational design, allowing investigation of the response to TOMAC when participants have no incentives for adherence.

## Materials and methods

### Study design

The THRIVE study is a multicenter, prospective, observational study enrolling patients prescribed TOMAC to treat RLS. The full study name is “A Post-Market Observational Clinical Study to Determine the Long-Term Effectiveness and Safety of the Noctrix TOMAC (Tonic Motor Activation) System for the Treatment of Restless Legs Syndrome—The THRIVE Study.” Patients prescribed TOMAC by any clinician in the United States were referred to one of 7 clinical trial centers for screening and enrollment. The study investigators at the clinical trial centers had no role in treatment of the patient, only in confirming eligibility and assessing Clinician Global Impressions-Improvement (CGI-I) score. There were no study requirements related to TOMAC usage or management of RLS. Enrolled patients consented to 1-year of follow-up, with primary and secondary outcome measures collected every 90 days relative to baseline. This analysis represents the first data available: the interim 90-day outcomes. The THRIVE study was conducted in accordance with the International Conference on Harmonization guidelines on good clinical practice and the Declaration of Helsinki. Each site investigator was responsible for adjudicating adverse events (AEs). The study protocol and informed consent were approved by a central institutional review board (Advarra, Columbia, MD, USA). All participants provided informed consent. The trial was preregistered (ClinicalTrials.gov number, NCT06076499) on October 6, 2023.

### Participants

All patients prescribed TOMAC in the US were invited to participate in THRIVE until the prespecified sample size of 325 was enrolled (refer to Effectiveness Analysis section below for sample size determination methodology). Screening was completed after prescription and prior to TOMAC activation; the only eligibility criteria were a diagnosis of RLS, ability to communicate via internet and phone, and ability to converse in English.

### Indicated intent-to-treat analysis population

Consistent with the prespecified statistical analysis plan (SAP), all analyses presented are specific to the indicated intent-to-treat (IITT) population which was comprised of patients who activated TOMAC and who met the US FDA indication for TOMAC listed in DEN220059: adults (aged ≥22) with moderate-to-severe refractory RLS who were not contraindicated [5, 7]. RLS diagnosis was confirmed based on International RLS Study Group diagnostic criteria [1]. Moderate-to-severe RLS was defined as IRLS total score ≥ 15 [12]. Refractory was defined as in the RESTFUL pivotal TOMAC trial [7] as having failed one or more of the following RLS medications—ropinirole, pramipexole, gabapentin, pregabalin, gabapentin enacarbil, and/or rotigotine—for at least one of the following reasons: intolerable adverse effects, symptoms of augmentation, up-titration needed due to reduced efficacy, and insufficient response at maximum approved, recommended, or tolerated dosage. Contraindications included active medical implants, epilepsy, known allergy to device materials, and/or metal implant at device site (not including knee replacements).

### TOMAC instructions for use

TOMAC instructions for use apply to all real-world clinical practice and are not specific to participation in this study. TOMAC is comprised of two therapy units instructed to be positioned bilaterally just below the knee over the head of the fibula where the peroneal nerve is close to the skin surface. The system is designed to stimulate the afferent fibers of the peroneal nerve. When activated, TOMAC delivers 4000 Hz electrical stimulation at stimulation intensities determined during the personalized activation session (always ≤40 mA). Patients are instructed to self-administer 30-min TOMAC sessions as needed after RLS symptoms start and before symptoms become severe. TOMAC sessions automatically turn off after 30 min. TOMAC is powered by a rechargeable battery. There is no limit to the number of sessions administered per day and no constraint on the times of day that TOMAC can be used.

Following TOMAC prescription, TOMAC devices are shipped to the patient in an inactive state and a “personalized activation session” with a certified technician is required to activate therapy delivery. This activation session includes titration of stimulation intensity, fitting, patient education on instructions for use, and activation of therapy.

### Study procedures

The following were assessed at baseline, day 90 and no times in between: IRLS, Medical Outcomes Study Sleep Problems Index II (MOS-II) [13], frequency of RLS symptoms (same wording as IRLS question 7 but with 8 possible responses: 0–7 days per week), Generalized Anxiety Disorder-7 (GAD-7) [14], and Patient Health Questionnaire-8 (PHQ-8) [15]. The following were assessed only at day 90 compared to baseline: CGI-I score [16] and Patient Global Impressions-Improvement (PGI-I) score [16].

The primary effectiveness endpoint was mean change in IRLS score from baseline to day 90. The secondary effectiveness endpoints were the following: mean change in MOS-II score from baseline to day 90, mean CGI-I score assessed at day 90 relative to baseline, mean PGI-I score assessed at day 90 relative to baseline, and mean change in frequency of RLS symptoms from baseline to day 90. Additional endpoints were the CGI-I and PGI-I responder rate (proportion of responses of “much improved” or “very much improved”) and improver rate (proportion of responses of “minimally improved,” “much improved,” or “very much improved”). The change from baseline to day 90 in the proportion of patients with moderate–severe anxiety (GAD-7 ≥ 10) and depression (PHQ-8 ≥ 10) were exploratory outcomes.

All baseline assessments (other than iron testing) were performed prior to the personalized activation session and were completed remotely (*n* = 230) or in-person (*n* = 2); the latency between baseline and activation was minimized to the greatest extent possible based on patient availability (e.g. the day before). Patients who failed to attend and complete the personalized activation session were withdrawn from the study and not included in the analysis population. Patients completed a blood draw (within 4 weeks of enrollment; 213/232 of IITT population completed blood draw) with iron panel analysis (Quest or LabCorp based on location). As the THRIVE study was purely observational, patients’ physicians were not informed about results of iron panel, and no treatment was given based off results. Day 90 assessments were completed remotely. Patients self-identified their race and ethnicity at enrollment.

For additional exploratory analyses, patients filled out a biweekly survey remotely. On this questionnaire, participants were asked to rate their overall RLS severity using an 11-point numerical rating scale (NRS): “*On a typical night in the past 2 weeks, rate the average severity of your RLS symptoms on a scale from 0 (no symptoms) to 10 (very severe symptoms)*.” Participants were also asked to rate acute RLS severity before—and at the end of—individual 30-min TOMAC sessions using a similar methodology to Buchfuhrer et al., 2021 [8]: “*For your 30-minute therapy sessions in the past 2 weeks, rate your typical RLS symptoms on a scale from 0 (no symptoms) to 10 (very severe symptoms): Before (immediately before you turned therapy on), and At the end of therapy (immediately before the therapy session ended)*.” Patients also reported changes in their RLS medication dosage on this biweekly survey.

### Effectiveness analysis

The primary and secondary effectiveness endpoints were prespecified in the order listed in the Study Procedures section above and assessed at day 90 compared to baseline. All effectiveness analyses were performed on the IITT population. Patients who exited before day 90 were asked to complete outcomes at time of exit with these values imputed for their day 90 results using the last observation carried forward method. For patients who did not complete this exit assessment and had no observed outcomes, Markov Chain Monte Carlo (MCMC) multiple imputation was used for all primary and secondary endpoints. Consistent with the SAP for interim reports (prior to 1-year), confidence intervals (CIs) were employed as the primary data representation; hypothesis testing via one-sided *t*-tests were employed exclusively as supportive analysis. Assuming 90% power and the IRLS mean and standard deviation (*SD*) observed after 8 weeks of TOMAC treatment in the RESTFUL study [7], a sample size of only *N* = 10 is required for a one-sided one sample *t-*test with an alpha level of 0.0125. The larger sample size of 325 was prespecified and selected for three reasons: (1) to increase precision of estimates for the primary endpoint (to a 95% CI margin of error of 0.69 points), (2) to provide greater statistical power to identify subgroup differences, and (3) to be on par with the largest clinical trials for FDA approved RLS medications [17]. All statistical analysis was conducted using R version v4.5.1 [18]. MCMC multiple imputation was conducted using the R mice v3.18.0 package [19]. Estimated marginal means were calculated using the R emmeans v1.11.2-8 package [20]. The study protocol and SAP are available in eSAP 1 and eSAP 2, respectively.

### Sensitivity analysis

A sensitivity analysis was conducted to assess if there was concomitant initiation of iron therapy, initiation sleep disorder treatment, or cessation of antihistamines. Initiation of intravenous or oral iron, continuous positive airway pressure (CPAP) machine, and other prescription medications for insomnia were analyzed in the 90 days before enrollment or during the study. Cessation of antihistamines was assessed in the same period. As initiation date of CPAP was not reported, patients with a new obstructive sleep apnea diagnosis in the 90 days before enrollment or during the study who also reported using a CPAP machine were analyzed. Analysis of covariance (ANCOVA) was used to calculate *p*-values comparing patients who initiated one of these new therapies to patients who did not. Cohen’s *d* (difference in group means in units of standard deviation) was used to assess effect size.

The robustness of the primary outcome with respect to the imputation approach was evaluated using two alternative, simpler imputation methods. The “worst-case” approach assumed that outcomes for patients without data were the same as outcomes for patients who returned the TOMAC device for a refund (“returners”). The “matched imputation based on exit status” approach assumed that outcomes for returners without data were the same as returners with data (same as the worst-case approach) but that outcomes for nonreturners without data were the same as nonreturners with data. Since baseline IRLS score was highly correlated with IRLS change (Pearson’s correlation = −0.44), both approaches also adjusted for differences in baseline IRLS score between the patients with missing data and comparison group with data based on a linear fit.

### Safety analysis

All safety analyses were conducted on both the IITT and the full population. The count and proportion of participants reporting AEs with new onset or worsening (relative to baseline) were reported. AEs were coded and summarized at the participant level by Medical Dictionary for Regulatory Activities System Organ Class and Preferred Terms and by seriousness, severity, and relationship to the device.

### Exploratory analyses

As noted in Study Procedures above, exploratory assessments included an electronic biweekly questionnaire where participants rated overall RLS severity and acute RLS severity before and at the end of individual TOMAC sessions. Patients with missing survey entries due to device return were imputed as no acute change in RLS symptoms. Patients with missing survey entries for other reasons (e.g. forgot to fill out the questionnaire) were omitted from analysis. For overall RLS severity, all missing entries were imputed by linearly interpolating between observed responses. The overall RLS severity at Week 0 was imputed from the baseline IRLS score by dividing the IRLS score by 4 and rounding to the nearest whole number. If no response was given for the final biweekly survey, the overall severity was imputed using the day 90 IRLS score.

The GAD-7 [14] andPHQ-8 [15] were administered at baseline and day 90 to assess changes in the prevalence of clinically significant anxiety and depression, respectively. The percentage of patients who met moderate-to-severe criteria for GAD-7 and PHQ-8 were reported (GAD-7 ≥ 10; PHQ-8 ≥ 10) [14, 15]. Patients whose data were imputed at day 90 for each questionnaire were assumed to have no change. The percentage of patients with moderate-to-severe anxiety or depression at baseline compared to day 90 were tested for significance using McNemar’s test.

Analysis was conducted to determine which components of sleep were improved in response to TOMAC. Change in each item of the MOS sleep scale was tested for significance using a one-sided *t-*test with an alpha level of 0.025 and the Holm-Bonferroni method to account for multiple comparisons.

For assessing potential predictors of response, imputed data were used for all analyses. Categorical variables were evaluated using linear models with subgroup category as the dependent variable. Continuous variables were evaluated using the Pearson correlation coefficient. Correction for baseline severity was performed using ANCOVA and/or the partial Pearson correlation. All analyses were performed using statistical packages in R version v4.5.1 [18]. Two-sided tests of significance with an alpha level of 0.025 were used with the Holm-Bonferroni method to account for multiple comparisons. RESTFUL criteria were only compared if at least 10 patients were on both sides of the comparison. The categorical variable for prescriber type was determined from the National Provider Identifier registry [21]. Prescriber type was listed as either board-certified sleep specialist, board-certified neurologist, board-certified pulmonologist, or primary care practitioner if none of the above specialties were listed and provider was classified as “Internal Medicine,” “Family Medicine,” “Nursing Service Providers,” “Physician Assistants & Advanced Practice Nursing Providers,” or “Nursing Service Providers.”

### Analysis of changes to adjunctive medication

Changes to adjunctive medication dosage were determined by comparing dosage of prescription RLS medications at baseline compared to day 90. Participants who reduced one RLS medication and increased another RLS medication were not included in the analysis. Reductions and increases in dosage were analyzed for the three most common classes of RLS medication after converting to milligram equivalents (ME): DAs (pramipexole ME), alpha-2-delta ligands (gabapentin ME), and opioids (morphine ME). Starting or stopping of other prescription medications for RLS compared to baseline were also analyzed. Previously proposed conversion factors [22–28] were employed and are listed in Table S1. To summarize reductions and increases, the percentage change in dosage was calculated for each participant and then averaged over participants.

### Data availability

The deidentified data that support the findings of this study are available from the corresponding author upon reasonable request.

## Results

### Patient characteristics

**Figure 1:**
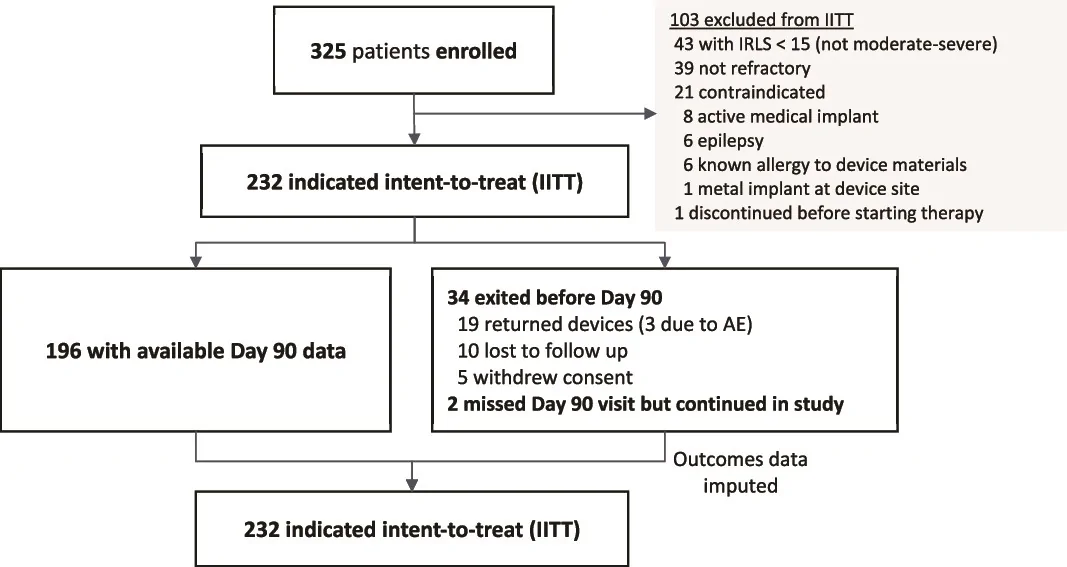
Participant disposition. Flow chart of IITT analysis population determination and imputation. Exclusions from the indicated intent-to-treat population were not mutually exclusive. AE, adverse event; IRLS, international RLS study group rating scale; IITT, indicated intent to treat.

Between November 15, 2023, and June 24, 2025, 329 participants were screened for eligibility and 325 were enrolled (Figure 1). Consistent with the prespecified SAP, the primary IITT analysis population included 232 participants and excluded 103: 43 with baseline IRLS score below 15, 39 nonrefractory, 21 contraindicated, and 1 who discontinued before starting TOMAC due to complications from a recent knee surgery. The IITT population included patients prescribed TOMAC by 145 unique healthcare providers. In the IITT population, the average time from enrollment to titration was 4.4 days (*SD*, 7.1 days). Of the 232 patients in the IITT population, 196 completed through day 90, 13 exited before day 90 and provided outcomes data at study exit, and 23 had no data available at any time point after baseline (21 exited, 2 continued in study but were unreachable at day 90); data were imputed for the 23 patients with unavailable data as described in Methods. Of the 34 patients who exited the study, 19 returned the device for a refund (16 due to inadequate benefit, 2 due to discomfort, 1 due to muscle spasms, 0 due to malfunction), 10 were lost to follow up, and 5 withdrew consent.

Table 1 describes participant baseline characteristics. Mean age was 67.2 years, 60% were female, mean IRLS score was 25.7 (*SD*, 5.8), and 90% of patients were taking adjunctive RLS medication (45% one medication, 45% two or more medications). The most common classes of adjunctive RLS medication were DAs (58%) and alpha-2-delta ligands (53%). On average, patients were prescribed their first RLS medication 14.3 years ago, and 8% of patients were prescribed their first RLS prescription within 1 year. Iron testing indicated that 7% of patients had low ferritin levels (≤30 μg/L) and 12% of patients had transferrin saturation below 20%. The most frequently diagnosed comorbidity was obstructive sleep apnea (47%). Baseline questionnaires also suggested high prevalences of moderate-to-severe insomnia (55% ISI ≥ 15) [29], anxiety (27% GAD-7 ≥ 10) [14], and depression (28% PHQ-8 ≥ 10) [15].

**Table 1:** Demographic and Clinical Characteristics.

**Variable**	**IITT (*n* = 232)**
**Sex, No. (%)**	
Female	139 (60%)
Male	93 (40%)
**Age**	
Age, mean (*SD*), y	67.2 (10.9)
Age ≥ 65 years, No. (%)	160 (69%)
**Race, No. (%)**	
American Indian or Alaska Native	0 (0%)
Asian	9 (4%)
Black or African American	4 (2%)
Native Hawaiian or Pacific Islander	1 (0%)
White	225 (97%)
**Ethnicity, No. (%)**	
Hispanic or Latino	9 (4%)
Not Hispanic or Latino	223 (96%)
**RLS history**	
Duration of RLS symptoms, mean (*SD*), y	28.9 (19.1)
Years since first RLS prescription, mean (*SD*), y	14.3 (11.6)
Familial RLS No. (%)	128 (55%)
Age of RLS symptom onset, mean (*SD*), y	38.3 (19.4)
IRLS total score, mean (*SD*)	25.7 (5.8)
**Iron, mean (*SD*)**	
Serum iron (μg/dL)	95.2 (40.8)
TIBC (μg/dL)	313.2 (49.6)
TSAT (%)	29.9% (11.0)
Serum ferritin (μg/L)	166.8 (141.7)
**Classes of current RLS medication, No. (%)**	
Dopamine agonists	135 (58%)
Alpha-2-delta ligands	124 (53%)
Opioids	58 (25%)
Benzodiazepines	8 (3%)
Other	11 (5%)
No prescribed RLS medications	23 (10%)
Multiple classes of RLS medications	104 (45%)
**Comorbidities, No. (%)**	
OSA	109 (47%)
Neuropathy	44 (19%)
Diabetes	31 (13%)
Insomnia (ISI ≥ 15)	128 (55%)
Anxiety (GAD-7 ≥ 10)	62 (27%)
Depression (PHQ-8 ≥ 10)	66 (28%)

Abbreviations: GAD-7, Generalized Anxiety Disorder-7; IITT, indicated intent to treat; IRLS, International RLS Study Group Rating Scale; ISI, Insomnia Severity Index; OSA, obstructive sleep apnea; PHQ-8, Patient Health Questionnaire-8; RLS, restless legs syndrome; *SD*, standard deviation; TIBC, total iron-binding capacity; TSAT, transferrin saturation.

### Effectiveness endpoints

Outcomes of primary and secondary effectiveness endpoints are presented in Table 2. The primary endpoint, mean IRLS score, improved from 25.7 at baseline to 17.3 at day 90 (change = −8.4; 95% CI: −7.4 to −9.4). Mean CGI-I score relative to baseline at day 90 was 2.4 (95% CI: 2.2 to 2.6) and the responder rate was 64% (very much or much improved). Mean PGI-I score relative to baseline at day 90 was 2.6 (95% CI: 2.4 to 2.8) and the responder rate was 54%. Mean MOS-II score improved from 53.6 at baseline to 40.8 at day 90 (change = −12.8; 95% CI: −10.3 to −15.3). Mean days/week with RLS symptoms reduced from 6.1 at baseline to 4.8 days at day 90 (change = −1.3; 95% CI: −0.9 to −1.7), corresponding to an increase in symptom-free days per week from 0.9 to 2.2. All primary and secondary outcomes were statistically significant (*p* < .001).

**Table 2:** Effectiveness Outcome Measures.

**Outcome**	**IITT (*n* = 232)**
**Primary effectiveness endpoint, mean (95% CI)**	
IRLS total score change from baseline to day 90	−8.4 (−7.4, −9.4)
**Secondary effectiveness endpoints, mean (95% CI)**	
CGI-I score at day 90	2.4 (2.2, 2.6)
PGI-I score at day 90	2.6 (2.4, 2.8)
MOS-II score change from baseline to day 90	−12.8 (−10.3, −15.3)
Days/week with RLS symptoms change from baseline to day 90	−1.3 (−0.9, −1.7)
**Additional endpoints, %**	
CGI-I responder rate at day 90	64%
CGI-I improver rate at day 90	82%
PGI-I responder rate at day 90	54%
PGI-I improver rate at day 90	78%

All reported outcomes are data imputed using MCMC multiple imputation methods.

Abbreviations: CGI-I, Clinical Global Impressions-Improvement; CI, confidence interval; IITT, indicated intent to treat; IRLS, International RLS Study Group Rating Scale; MOS-II, Medical Outcomes Study Sleep Problem Index II; PGI-I, Patient-Global Impressions-Improvement.

### Exploratory effectiveness analysis

**Figure 2:**
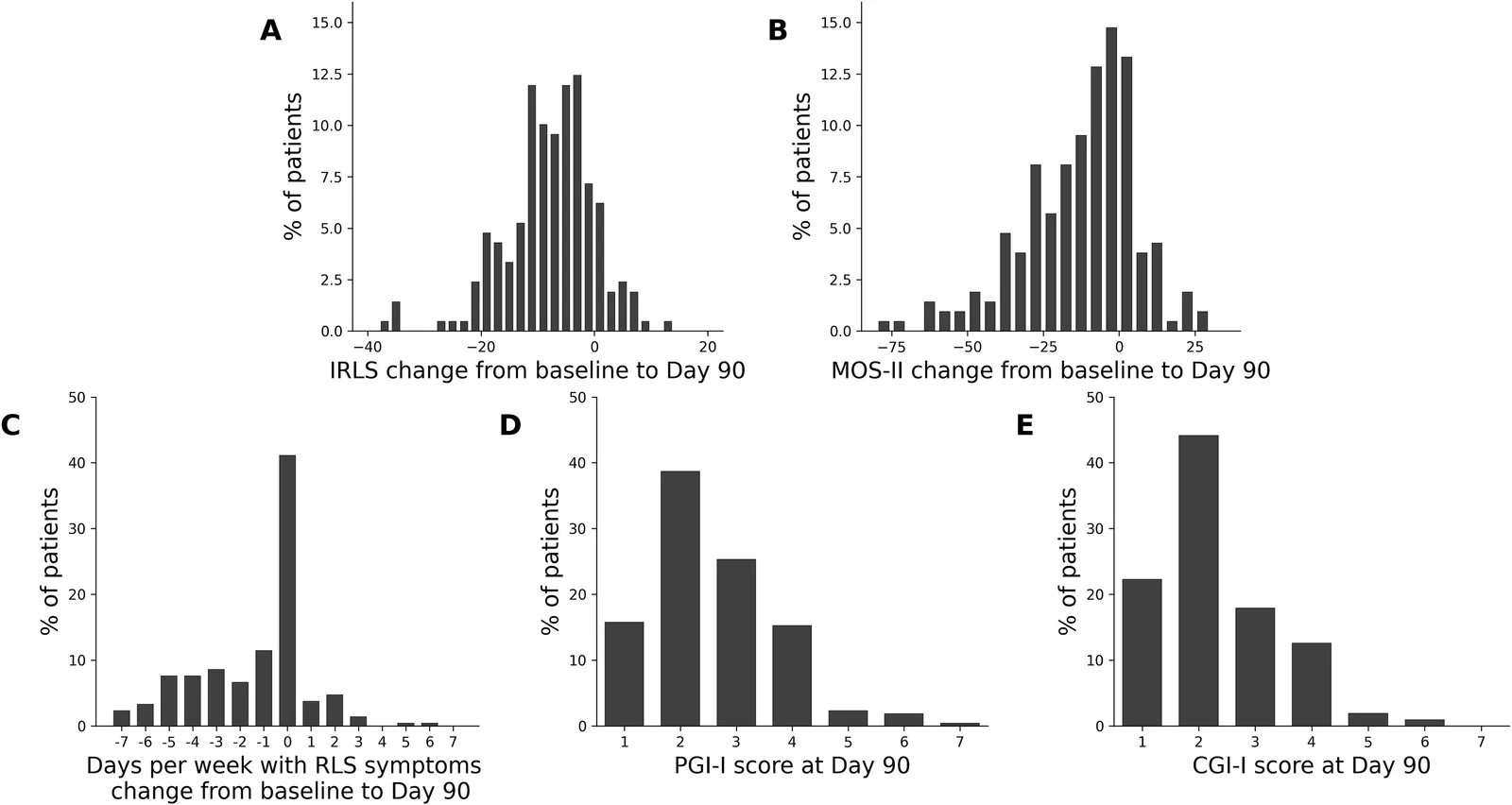
Distributions of effectiveness outcome measures at day 90 compared to baseline. Distributions at day 90 compared to baseline for (A) change in IRLS score, (B) change in MOS-II score, (C) change in days/week with RLS symptoms, (D) PGI-I score, (E) CGI-I score. For PGI-I and CGI-I scores, 1: very much improved, 2: much improved, 3: minimally improved, 4: no change, 5: minimally worse, 6: much worse, 7: very much worse. Data imputed with MCMC multiple imputation are not included in distributions; IRLS: *n* = 209, MOS-II: *n* = 210, days/week: *n* = 209, PGI-I: *n* = 209, CGI-I: *n* = 206. CGI-I, Clinical Global Impressions-Improvement; IRLS, International RLS Study Group Rating Scale; MCMC, Monte Carlo Markov Chain; MOS-II, Medical Outcomes Study Sleep Problem Index II; PGI-I, Patient-Global Impressions-Improvement.

Figure 2 illustrates the distribution of observed responses for all patients and outcome measures. IRLS score improved by a clinically significant amount (≥3 points) [30, 31] in 79% of patients and improved by ≥10 points in 42% of patients; IRLS score worsened by ≥3 points in 6% and by ≥10 points in 0.48%. At day 90, 38% of patients had IRLS score < 15, the typical threshold for moderate-to-severe RLS [7, 32, 33]. The distribution of change in days/week of RLS symptoms was skewed left (skewness: −0.45), with reduction in 48% of patients, no change in 41%, increase in 11%, and 36% reporting a reduction of ≥2 days per week. The CGI-I and PGI-I distributions were skewed right (skewness: both 0.84). For CGI-I and PGI-I scores, 82% and 78% of patients improved, respectively (Table 2, Improver rate).

We assessed changes in each item of the MOS sleep scale to better understand which aspects of sleep are most improved (Table S2). All items improved by a significant amount following Holm-Bonferroni correction except for items 5 (shortness of breath) and 11 (taking naps during the day). Significant improvements with effect size >0.4 (Cohen’s *d*) were observed in items 12 (get the amount of sleep needed), 4 (feel rested in morning), 6 (feel drowsy during day), 3 (sleep not quiet), 8 (awaken and trouble falling back asleep), and 1 (time to fall asleep). Together, these results show that TOMAC improves sleep adequacy, daytime somnolence, sleep onset latency, and sleep disturbance.

The incidence of probable moderate-to-severe anxiety (GAD-7 ≥ 10) decreased from 27% to 19% from baseline to day 90 (62/232 to 43/232; *p* = .007) and the incidence of probable moderate-to-severe depression (PHQ-8 ≥ 10) decreased from 28% to 18% (66/232 to 42/232; *p* < .0001). The insomnia severity index was not assessed at day 90.

### Safety

Device-related AEs occurred in 8% of the IITT population by day 90 and were always moderate or mild in severity (Table 3). Three patients discontinued in the study due to device-related AEs: 2 due to discomfort, and 1 due to muscle spasms. The most common AEs were discomfort (4%), and administration site reactions (mild 2%, moderate 1%). AEs were also assessed for the full THRIVE population, and rates and classification of AEs were similar to the IITT population (Table S3). As clinicians can prescribe TOMAC off-label, the full population includes patients who were contraindicated for TOMAC. Patients who were contraindicated for TOMAC because of active medical implants (*n* = 8) and epilepsy (*n* = 6) did not have any device-related AEs by day 90.

**Table 3:** Treatment-Emergent Adverse Events.

**Variable**	**By day 90 visit** **IITT (*n* = 232)**
AE, *n* (%)	
Any	67 (29%)
Device-related	18 (8%)
Serious or severe AE, *n* (%)	
Any	8 (3%)
Device-related	0 (0%)
Discontinuation due to AE, *n* (%)	
Any	5 (2%)
Device-related	3 (1%)
MedDRA preferred term^*^, *n* (%)	
Discomfort	9 (4%)
Administration site reaction, mild	4 (2%)
Administration site reaction, moderate	3 (1%)

Abbreviations: AE, adverse event; IITT, indicated intent to treat; MedDRA, Medical Dictionary for Regulatory Activities.

^*^MedDRA preferred terms for top 3 most common device-related AEs.

### Changes in adjunctive medication

Next, we compared the dosage of adjunctive RLS medication between study entry and day 90. Patients who reduced one medication and increased another were not included in this analysis. Pooled across all prescription RLS medications, 19% of participants reduced their dosage while 11% increased their dosage (Table 4), and medication reductions were more common than increases (*p* = .01). Within the three main medication classes, this difference was significant for DAs (*p* = .02, Table 4), but not for alpha-2-delta ligands or opioids (*p* > .2, Table 4). The average IRLS change from baseline to day 90 in patients who reduced medication was −8.1 (95% CI: −6.3 to −9.9).

**Table 4:** Changes in Adjunctive RLS Medication

**IITT (*n* = 232)**	**Dopamine agonist changes**	**Opioid changes**	**Alpha-2-delta ligand changes**	**Any medication change** ^*^
**Total changes**				
Decreased dose^†^, No. (%)	24 (18%)	12 (21%)	13 (11%)	45 (19%)
Increased dose^‡^, No. (%)	11 (8%)	10 (17%)	8 (7%)	26 (11%)
*p-*value two proportion *z* test	0.02	0.64	0.25	0.01
**Medication reductions** ^†^				
Average starting dose, PPX ME, morphine ME, or GBP ME, mean (*SD*)	0.84 (0.73)	41.5 (40.1)	798.5 (419.2)	n/a
Average reduction, PPX ME, morphine ME, or GBP ME, mean (*SD*)	−0.33 (0.22)	−17.3 (20.3)	−334.8 (137.3)	n/a
Average % reduction	−52%	−50%	−50%	−51%
IRLS change, mean (*SD*)	−7.6 (6.7)	−7.5 (6.4)	−9.7 (7.1)	−8.1 (6.3)
MOS-II change, mean (*SD*)	−13.6 (17.9)	−13.7 (15.2)	−15.5 (25.3)	−13.8 (19.5)
**Medication increases** ^‡^				
Average starting dose, PPX ME, morphine ME, or GBP ME, mean (*SD*)	0.25 (0.28)	28.8 (29.5)	246.4 (338.8)	n/a
Average increase, PPX ME, morphine ME, or GBP ME, mean (*SD*)	0.27 (0.21)	29.5 (27.9)	447.3 (182.3)	n/a
Average % increase	n/a	n/a	n/a	n/a
IRLS change, mean (*SD*)	−4.1 (6.8)	−12.1 (12.6)	−8.8 (6.3)	−8.6 (9.9)
MOS-II change, mean (*SD*)	−12.0 (24.6)	−8.9 (23.8)	−12.0 (10.8)	−12.1 (21.5)

Abbreviations: GBP, gabapentin; IITT, indicated intent to treat; ME, mg equivalent; PPX, pramipexole; RLS, restless legs syndrome; *SD*, standard deviation.

^*^Any medication changes also include benzodiazepines and other medications prescribed for RLS.

^†^Decreases/reductions in medication are patients who ONLY decreased medication.

^‡^Increases in medication are patients who ONLY increased medication.

**Figure 3:**
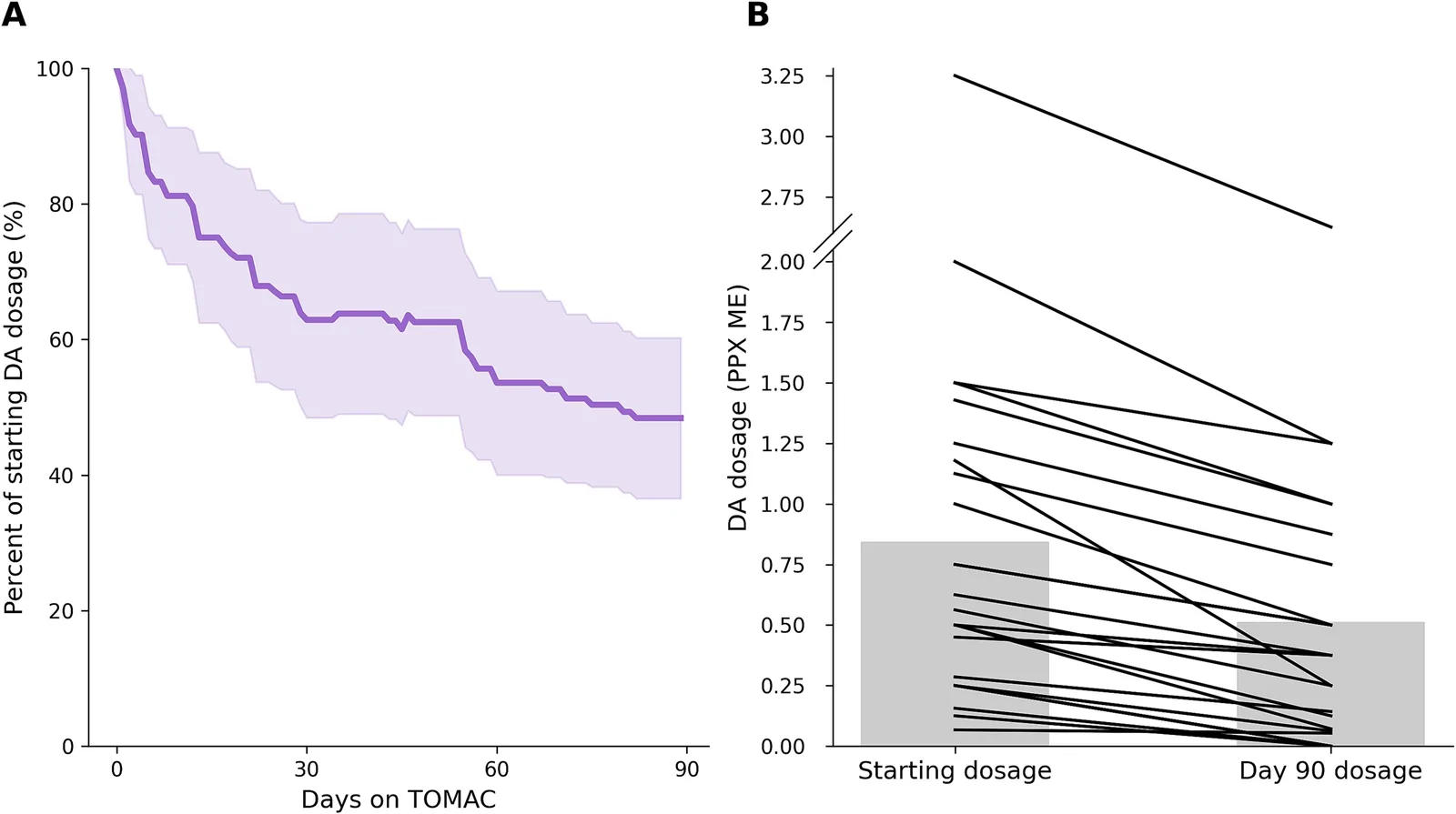
Reductions in dopamine agonist medication dosage. (A) Average percentage of starting DA dose for the 24 patients who reduced DA. Shaded area shows 95% confidence interval of mean. (B) Starting and day 90 dosages of DA converted to PPX ME for the 24 patients who reduced DA. Bars show average starting and day 90 dosage. DA, dopamine agonist; ME, mg equivalent; PPX, pramipexole.

Of the 135 patients taking adjunctive DAs at enrollment, 24 reduced DA dosage at day 90 (“DA reducers,” 18%) whereas 11 increased DA dosage (*p* = .02, Table 4). For the 24 DA reducers, DA dosage reduction was 52% (Figure 3). Most DA reducers (63%) decreased medication dosage a single time as opposed to incremental reduction; the first reduction was an average of 39% of the starting dose. Of the 24 patients who reduced their DA dosage at any point in the first 90 days (without increasing other medication), all 24 maintained a reduction in DA dosage at day 90 (100% success rate). Favorable long-term tolerability of DA dose reduction was indicated by the magnitude of average IRLS change from baseline to day 90 among DA reducers (mean IRLS change: −7.6; 95% CI: −4.9 to −10.3). Moreover, favorable short-term tolerability was indicated as well as only 20% of DA reducers reported a biweekly worsening of overall RLS severity of 2 points or more (0 to 10 scale) on the first biweekly questionnaire after DA reduction (Figure S1).

### Timescales of TOMAC response

**Figure 4:**
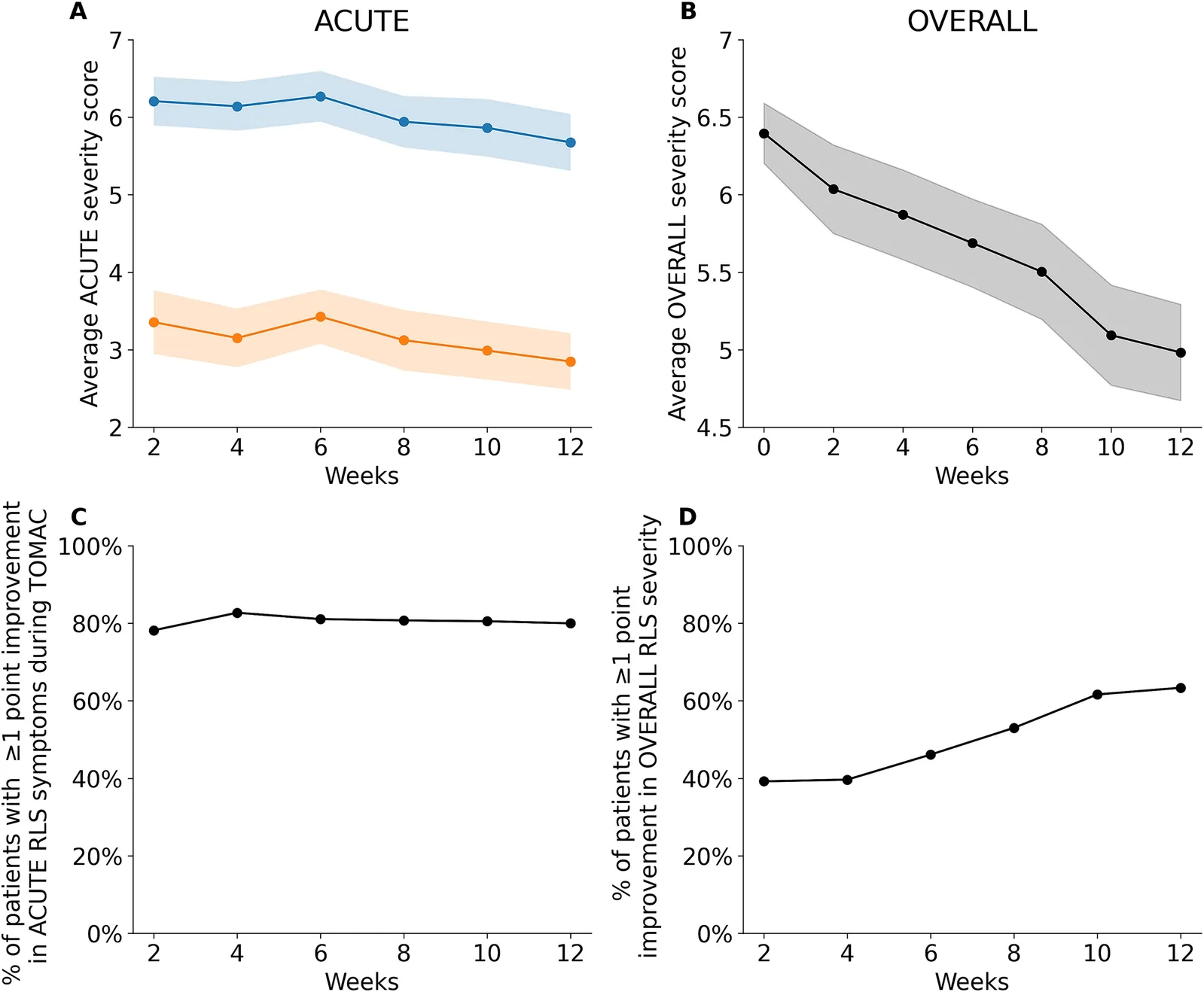
Timescales of response to TOMAC. (A) Acute RLS severity scores. Topmost line/blue: average acute RLS severity score immediately before TOMAC session started for each survey (0–10 scale). Bottommost line/orange: average acute RLS severity score immediately before TOMAC session ended for each survey (0-10 scale). Shaded area 95% CI (B) Change in overall RLS severity score. Average overall RLS severity score per biweekly survey (0–10 scale). Shaded area 95% CI. Week 0 data was estimated based on baseline IRLS (see Materials and Methods section). (C) Percent of patients with reduction in acute RLS severity score at the end of TOMAC sessions compared to just before TOMAC for each survey. (D) Percent of patients with improvement in overall RLS severity score compared to baseline for each survey. CI, confidence interval, RLS, restless legs syndrome; TOMAC, tonic motor activation.

Next, we assessed the timescale of changes in TOMAC response and RLS improvement. Whereas IRLS was only assessed at study entry and day 90, electronic patient-reported questionnaires assessed overall RLS severity (0 to 10 scale) and acute RLS symptom severity (0 to 10 scale) before and at the end of TOMAC sessions on a biweekly basis. Figure 4 shows plots of both overall RLS severity and acute RLS symptom relief. The percentage of patients with at least a 1-point reduction in acute RLS symptom severity score at the end of a TOMAC session compared to just before was 78% at week 2, 81% at week 6, and 80% at week 12 (Figure 4C). Additionally, patients reported an average change in acute RLS symptom severity during TOMAC sessions of −2.9 (out of 10) at week 2, −2.8 at week 6, and −2.8 at week 12, with an overall average change over weeks 2 through 12 of −2.9 points (Figure 4A). The percentage of patients with at least a 1-point reduction in overall RLS severity score compared to baseline was 39% at week 2, 46% at week 6, and 63% at week 12 (Figure 4D). These results show that acute relief from RLS symptoms is common in the first 2 weeks and is maintained over 12 weeks of TOMAC use, whereas overall RLS severity reduction takes longer to reach its maximum.

### Predictors of response

**Figure 5:**
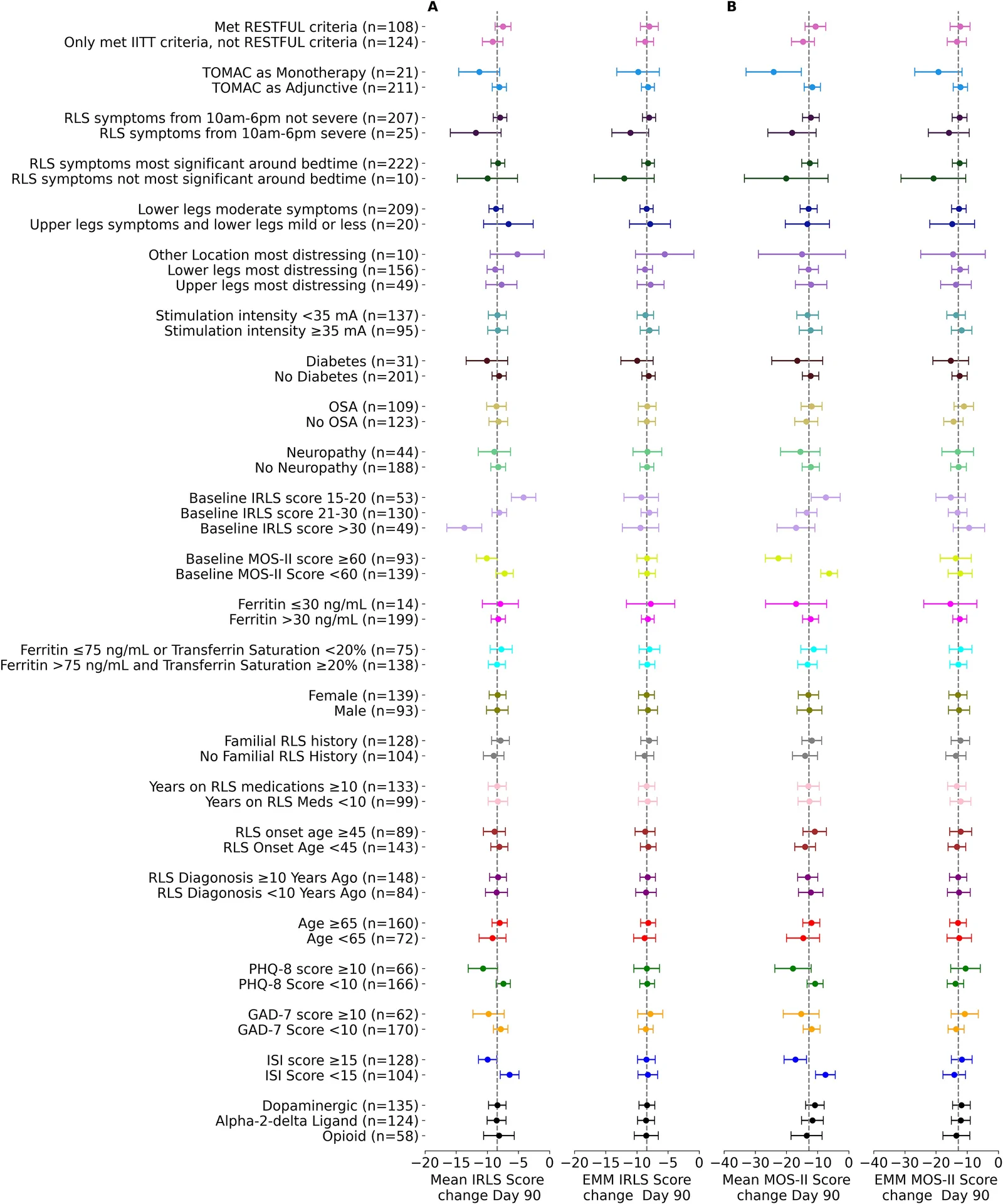
Subgroup comparisons in IRLS and MOS-II change. Mean change and 95% confidence intervals in (A) IRLS score and (B) MOS-II score at day 90 across different subgroups. Left panels show the mean change before correcting for baseline values. Right panels show the estimated marginal means of each subgroup correcting for either baseline (A) IRLS score or (B) MOS-II score. Dotted line shows overall average for IITT population. EMM, estimated marginal mean; GAD-7, Generalized Anxiety Disorder-7; IITT, indicated intent to treat; IRLS, International RLS Study Group Rating Scale; ISI Insomnia Severity Index; MOS-II, Medical Outcomes Study Sleep Problem Index II; OSA, obstructive sleep apnea; PHQ-8, Patient Health Questionnaire-8.

Exploratory analysis was conducted to determine if any baseline characteristics were related to differences in outcomes. Changes in IRLS score and MOS-II score were compared across 36 different categories including medical history, RLS history, adjunctive RLS medications, and demographics, and the Holm-Bonferroni method was used to address multiple comparisons (Figure 5, Figure S2, and Tables S4 and S5). Using this approach, higher baseline IRLS values were associated with greater IRLS reduction (*p* < .0001, Figure 5A, left  panel) and higher baseline MOS-II values were associated with greater MOS-II reduction (*p* < .0001, Figure 5B, left  panel). This may be due to regression to the mean as reported previously [34], as evidenced by the fact that PGI-I and CGI-I scores were not significantly correlated with IRLS or MOS-II baseline scores (*p*-values > .18, Tables S6 and S7). Therefore, we corrected for baseline IRLS and MOS-II values as continuous covariates respectively (Figure 5A and B, right  panels). Prior to this correction, but not after correction, baseline ISI and baseline PHQ-8 were associated with IRLS change and MOS-II change. After this correction, no characteristics were statistically significantly associated with outcomes.

The anatomical distribution of RLS symptoms did not affect outcomes. There was no significant difference between patients who reported their most distressing location as upper legs versus lower legs (ANCOVA: IRLS: *p* = .47, MOS-II: *p* = .65, Figure 5). Additionally, there was no difference between patients who reported upper legs symptoms and mild or no lower leg symptoms compared to patients with moderate or greater lower leg symptoms (ANCOVA: IRLS: *p* = .77, MOS-II: *p* = .65, Figure 5).

Patients who met the AASM clinical practice guidelines for intravenous iron infusion (transferrin saturation < 20% or ferritin ≤75 μg/L) [4] compared to those who did not also had no significant difference in IRLS and MOS-II changes (ANCOVA: IRLS: *p* = .69, MOS-II: *p* = .62, Figure 5). Additionally, patients who met the ferritin cutoff of ≤30 μg/L for iron deficiency anemia [35] had no significant differences in IRLS and MOS-II changes (ANCOVA: IRLS: *p* = .78, MOS-II: *p* = .50, Figure 5).

Current or past RLS treatment history also did not influence effectiveness. Outcomes for patients on TOMAC monotherapy (monotherapy) versus patients taking at least one adjunctive RLS medication (adjunctive) also did not differ significantly (ANCOVA: IRLS: *p* = .38, MOS-II: *p* = .08, Figure 5). Patients who were prescribed their first RLS medication within the last year also had no differences in outcomes (ANCOVA: IRLS: *p* = .54, MOS-II: *p* = .55, Figure S2). The number of different RLS prescription medications attempted before enrollment did not correlate with effectiveness (ANCOVA: IRLS: *p* = .55, MOS-II: *p* = .51, Tables S4–S6). Finally, patients who were prescribed TOMAC by a sleep specialist versus a primary care practitioner had no difference in outcomes (ANCOVA: IRLS: *p* = .65, MOS-II: *p* = .46, Figure S2).

During the RESTFUL study [7], there were 7 additional inclusion and 19 additional exclusion criteria in addition to the IITT criteria in THRIVE. There was no significant difference between outcomes of patients who met all RESTFUL criteria compared to those who only met the IITT criteria (Figure 5) neither before (*p* > .12) nor after (*p* > .50) correcting for baseline IRLS and MOS-II values. Additionally, none of the individual criteria predicted response after correcting for baseline IRLS and MOS-II values (*p* > .04, Figure 5 and Figure S2).

**Figure 6:**
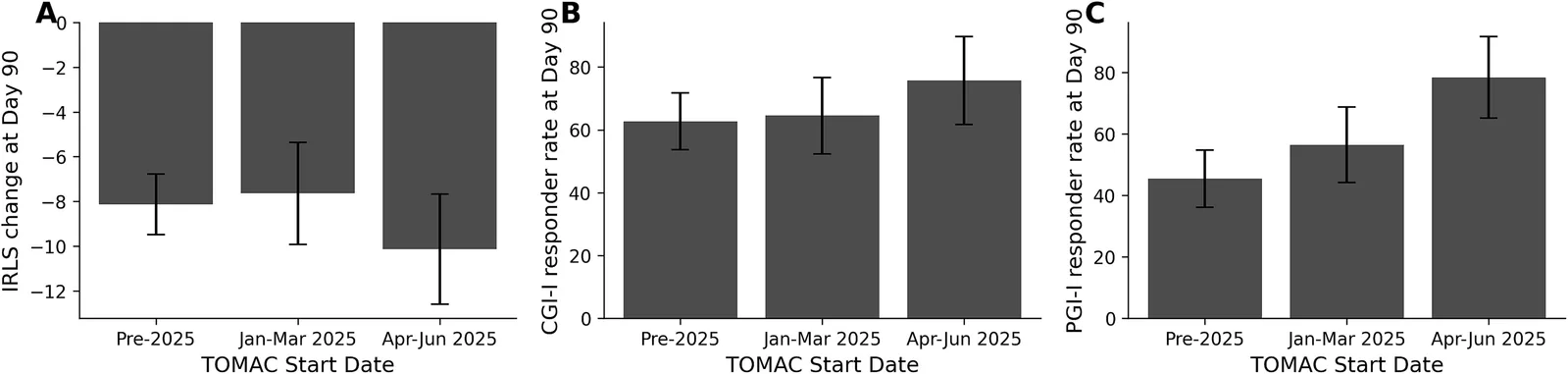
Outcomes by TOMAC start date. Mean outcomes and 95% CI for patients grouped by TOMAC start date: November 2023–December 2024 (*n* = 110), January–March 2025 (*n* = 62), and April–June 2025 (*n* = 37). Figure only includes reported outcomes with no imputed data. (A) Change in IRLS score at day 90 compared to baseline. (B) CGI-I responder rate (C) PGI-I responder rate. CI, confidence interval; CGI-I, clinical global impressions-improvement; IRLS, International RLS Study Group Rating Scale; PGI-I, Patient-Global Impressions-Improvement.

Finally, we assessed if patients who started TOMAC treatment more recently (e.g. in 2025) had different outcomes than patients who started earlier (e.g. in 2024). Patients were split into three groups based on TOMAC start date (Figure 6). PGI-I responder rate increased from 45% pre-2025 (95% CI: 36% to 54%) to 56% January–March 2025 (95% CI: 44% to 68%) to 78% April–June 2025 (95% CI: 65% to 91%). CGI-I responder rate increased from 63% pre-2025 (95% CI: 54% to 72%) to 65% January–March 2025 (95% CI: 53% to 77%) to 73% April–June 2025 (95% CI: 58% to 88%). Change in IRLS score at day 90 changed from −8.1 pre-2025 (95% CI: −6.7 to −9.5) to −7.6 January–March 2025 (95% CI: −5.3 to −9.9) to −10.1 April–June 2025 (95% CI: −7.6 to −12.6). Overall, there is a trend toward improvement over time, with PGI-I score and TOMAC start date being significantly correlated (*r* = −0.19, *p* = .005).

### Sensitivity analysis

We next assessed if concomitant changes to treatment of RLS or other sleep disorders—aside from TOMAC—could partially explain the improvements observed. Concomitant initiation of iron therapy was rare; a total of 5 patients had an intravenous iron infusion, and 1 patient started oral iron in the 90 days before enrollment or during the study. Furthermore, the 23 patients on iron therapy at baseline had no difference in effectiveness outcomes compared to all other patients (Figure S2, IRLS Cohen’s *d* = −0.16, all *p* > .12) The 30 patients who reported an unstable dosage of RLS medication in the month prior to enrollment also had no difference in effectiveness (Figure S2, IRLS Cohen’s *d* = 0.21, all *p* > .27). Initiation of new treatments for sleep disorders other than RLS were also rare; a total of 2 patients reported starting CPAP treatment in the 90 days before enrollment or during the study and 10 patients reported initiating other new treatments (Table S8). In total, 18 patients had a concomitant change in iron therapy or sleep disorder treatment and 214 patients did not. All primary and secondary effectiveness analyses were robust to removing these 18 patients from the analysis population (Table S8, IRLS Cohen’s *d* = 0.17, all *p* > .29). There was no evidence that changes in adherence to CPAP treatment could have explained the observed improvements in RLS or sleep; OSA patients treated with CPAP (*n* = 73) had similar effectiveness to all other patients (Figure S2, IRLS Cohen’s *d* = 0.09, all *p* > .14). Whereas antihistamines are known to worsen RLS symptoms [36] and thus cessation thereof could improve RLS symptoms, only 2 patients reported cessation of antihistamines within the 90 days before enrollment or during the study and there was no difference in effectiveness outcomes between the 22 patients taking antihistamines at enrollment and the other 210 patients (Figure S2, IRLS Cohen’s *d* = 0.08, all ANCOVA *p* > .53). We also assessed the robustness of the outcomes to two alternative approaches for imputing missing data for the 23 patients with no data available after baseline, a “worst-case” approach and a “matched imputation based on exit status” approach (see **Methods**). The worst-case approach yielded mean IRLS change of −8.2 (95% CI: −7.2 to −9.2), PGI-I responder rate of 52%, and CGI-I responder rate of 61% and the simplified approach yielded mean IRLS change of −8.4 (95% CI: −7.4 to −9.4), PGI-I responder rate of 54%, and CGI-I responder rate of 64%. Therefore, outcomes were robust to alternative imputation approaches.

## Discussion

The 90-day results from the ongoing THRIVE study demonstrate that TOMAC is safe and effective at reducing RLS symptoms in real-world clinical practice. Improvement in the primary endpoint—IRLS score—was clinically significant with an average change of −8.4 and 79% of patients achieving a reduction of at least 3 points, the threshold for a clinically meaningful response [30, 31], and 64% of patients were defined responders based on the CGI-I. The magnitude of these outcomes was similar to improvements reported in the pooled results from TOMAC clinical trials [37]. Additionally, 38% of patients achieved a large enough reduction in disease severity to be downgraded from moderate-to-severe (IRLS score ≥ 15) to mild (IRLS score < 15) RLS after 90 days of TOMAC therapy.

TOMAC also improved sleep and mental health. TOMAC led to improvement in multiple aspects of subjective sleep quality measured by the MOS sleep scale, including reduced sleep onset latency, sleep disturbance, and daytime somnolence, and increased sleep quantity. In addition to sleep improvement, the percentage of patients with PHQ-8 scores and GAD-7 scores above the thresholds associated with clinical depression and anxiety, respectively, declined by 10% and 8%. Clinical psychiatric evaluation would be helpful in future studies to confirm this finding.

TOMAC was well tolerated; there were no serious or severe device-related AEs and only 1% of patients discontinued therapy due to adverse effects. The most commonly reported AEs were discomfort and administration site skin reactions where the charge-dispersing electrodes contact the body, which ranged in severity from mild to moderate. Further investigation is needed to determine which of these adverse effects are caused by electrode materials versus electrical stimulation. The risk profile of TOMAC was favorable to DAs, alpha-2-delta ligands, and opioids, all of which have significant rates of neurological adverse effects and/or irreversible long-term adverse effects [30].

Effectiveness of TOMAC was generalizable to a broader patient population than previously studied. The eligibility criteria for this study were less restrictive than prior randomized clinical trials investigating TOMAC. For example, the RESTFUL study [7] had 12 inclusion and 24 exclusion criteria, many of which were intended to eliminate factors that could confound interpretation of results in a short-term sham-controlled design. In contrast, eligibility for this study required only a diagnosis of RLS and prescription of TOMAC and inclusion in the IITT analysis required only meeting the FDA indication of moderate-to-severe RLS with inadequate response to at least one prescription RLS medication (“refractory”). Despite this less restricted population, improvement in IRLS in THRIVE (8.4 points) was approximately equivalent to RESTFUL (8.7 points) and none of the inclusion or exclusion criteria from RESTFUL were associated with differential response in THRIVE. For example, (1) patients with severe daytime symptoms had equivalent responses to patients with only nighttime symptoms (Figure 5), and (2) patients with their most significant symptoms in their lower legs versus elsewhere had equivalent responses (Figure S2). These findings demonstrate that some of the RESTFUL study eligibility criteria may not be valid reasons for limiting TOMAC availability or insurance coverage.

Clinical history prior to starting TOMAC therapy had minimal impact on outcomes. Whereas higher baseline IRLS score (more severe RLS) predicted greater IRLS improvement, this did not predict lower CGI-I or PGI-I scores, suggesting regression to the mean. Similarly, higher baseline MOS-II score (worse sleep problems) predicted greater MOS-II improvement but not lower CGI-I or PGI-I scores.

A systematic review of randomized clinical trials [37] reported a weak effect of sex on PGI-I, but this effect was not observed in THRIVE (Figure 5 and Table S7) or in the open-label extension phase of the RESTFUL study [11]. Therefore, it is possible that this sex-based difference was related to differential placebo response as opposed to differential response to TOMAC, as speculated previously [37]. The prior review also found a weak association between higher stimulation intensity and less MOS-II improvement, which was not seen in THRIVE. One explanation for this difference is that some patients who participated in previous clinical trials may have been programmed to higher-than-optimal stimulation intensities that could interfere with falling asleep, and improvement in the TOMAC titration process prevented this problem in THRIVE.

Interestingly, there was no evidence of differential response for patients who reported RLS symptoms with an anatomical distribution in the lower legs and feet, the dermatomes associated with the peroneal nerve (Figure 5), than RLS symptoms at other locations. This is consistent with the therapeutic benefits of walking, which tends to relieve RLS symptoms throughout the body. Plausible mechanistic loci for this diffuse effect of TOMAC include intersegmental spinal cord, brain stem, or cortical modulation. Adjunctive RLS medications were prevalent in THRIVE patients with moderate-to-severe RLS, indicating inability to get sufficient relief from medication. However, category of baseline adjunctive medication did not influence TOMAC effectiveness (Figure 5), which could be an advantage of TOMAC over alpha-2-delta ligands, which tend to be less effective in patients with a long-history of dopaminergic medication [38]. Furthermore, consistent with a recent systematic review [37], there was no significant difference in outcomes between patients using TOMAC as monotherapy compared to patients who used TOMAC as an adjunctive to other RLS medication, and there was even a trend toward slightly greater effectiveness as monotherapy. This confirms that the observed improvements in RLS and sleep are due to TOMAC treatment as opposed to adjunctive medication.

As THRIVE is a real-world observational study, one important limitation is that there are uncontrolled factors which could potentially lead to changes in RLS severity unrelated to TOMAC. Our sensitivity analyses presented herein are intended to mitigate many of these potential confounds, by demonstrating that the observed improvements in RLS cannot be explained by concomitant initiation of iron therapy, initiation of therapeutics for RLS or other sleep disorders (other than TOMAC), or cessation of medications that exacerbate RLS. Additionally, we have provided evidence showing that recent initiation of RLS treatment was rare in this refractory population and could not explain the observed improvements. Taken together, these analyses suggest that the RLS improvements observed in THRIVE are predominately due to TOMAC therapy but do not conclusively rule out the potential impact of other uncontrolled factors, such as lifestyle changes and the placebo effect. For example, we did not collect information regarding exercise, alcohol, caffeine intake, or objective CPAP adherence [36].

Whereas clinical history and adjunctive medications did not explain interindividual variability in response, it is plausible that differences in adherence to instructions for use could explain much of this variation. Although we did not collect any objective measures of adherence, we found a significant trend toward an increase in PGI-I score over the period of THRIVE enrollment during which the TOMAC manufacturer made iterative attempts to improve the rigor of its titration and patient care program—programs directed at training patients to follow instructions for use. These changes may have led to improvements in outcomes, but we have no direct evidence to support this conclusion. If most variation in TOMAC response is related to differential usage behavior, this would reflect a similarity between TOMAC and CPAP, another patient-administered wearable therapeutic medical device indicated to treat a prevalent sleep disorder (obstructive sleep apnea) for which adherence is a key factor in determining effectiveness [39, 40].

Reductions in adjunctive medication dosage were well-tolerated. This finding is particularly noteworthy for DA medications which are notoriously difficult to taper due to the characteristic withdrawal response, which involves a dramatic 1–2 week increase in RLS severity and insomnia [41]. Furthermore, there is substantial clinical interest in reducing DA dosage to decrease the severity or risk of augmentation. Remarkably, for patients in THRIVE, there was no evidence of a significant increase in RLS severity in patients who reduced DA dosage at any point in time. These results suggest that substantial DA dosage reductions can be tolerated during TOMAC treatment. Considering the observational nature of this study, it is unclear why some patients (and their corresponding healthcare providers) attempted DA dose reduction and others did not. It will be important to analyze this over longer follow-up durations when 1-year THRIVE outcome data is available.

This study had several limitations. Whereas its observational design had the advantage of accurately representing real-world clinical practice, it results in a patient population that is heterogeneous and influenced by prescriber behavior, insurance coverage, and FDA indications for use. For example, the average patient age was greater than reported in RESTFUL, possibly because the insurance coverage rate for patients on Medicare (most of whom are 65 and older) is higher than the coverage rate for non-Medicare patients (most of whom are below the age of 65). Additionally, there were 145 unique prescribers who may have different strategies for prescribing TOMAC within the continuum of RLS care. The timing of the study within the first 1–2 years of TOMAC commercial availability means that these prescribers may include a disproportionately high number of early adopters and may not be representative of all healthcare providers who manage RLS. Another limitation is that there was no objective tracking of adherence or usage of TOMAC in this study and, thus, it cannot be determined if effectiveness was greater in patients that followed instructions for use compared to those who did not. While the 90-day duration is longer than the 8-week RESTFUL study duration [7], it is shorter than the 24-week duration of the RESTFUL extension study [11], which showed larger improvements with continued use. Therefore, future analyses are needed to establish longer-term effectiveness and should be conducted when 1-year outcomes are available from the THRIVE study. Finally, as this study was purely observational, there was no control sham group to assess placebo effect, which has been documented in RLS [42]. In the 3 previous randomized clinical trials that studied TOMAC [7–9], the change in IRLS score in the sham control group ranged from −3.0 to −3.8 points, and IRLS improvement was always significantly greater in the TOMAC group. The 8.4 point improvement observed here exceeded this sham response by between 4.6 and 5.4 points.

There were two timescales of improvement, more rapid acute relief and more gradual overall RLS improvement. The percentage of patients reporting acute relief increased over the first 6 weeks of usage and then plateaued. One possibility is that this 6-week period corresponds to patients learning how to correctly operate therapy, including positioning the electrodes over the peroneal nerve and increasing stimulation intensity to a level sufficient to activate the nerve. In contrast, overall RLS improvement continued to increase gradually over the first 12 weeks of usage. This gradual improvement may be due to patients developing habits such as using TOMAC consistently at all times of day that they have RLS symptoms, which may improve effectiveness. Another possibility is that neural plasticity at the spinal or corticospinal level results in gradual improvement.

## Supplementary Material

Supplemental_Figures_and_Tables_THRIVE_zsag140

eSAP1_zsag140

eSAP2_zsag140
