# Supplementary material for: Real-world effectiveness and safety of tonic motor activation therapy for refractory restless legs syndrome: 90-day outcomes from the THRIVE prospective observational study
Source: Sleep. 2026 Jun 3;49(9):zsag140. doi: 10.1093/sleep/zsag140 (PMC13553290; doi:10.1093/sleep/zsag140)
Supplement: Supplementary file 1 [file esap1_zsag140.pdf]

Real-world effectiveness and safety of tonic motor activation therapy for refractory restless legs syndrome: 90-day outcomes from the THRIVE prospective observational study

Haramandeep Singh*[1]; Mark J. Buchfuhrer*[2]; Joseph Ojile[3]; Asim Roy[4]; Robert Joseph Thomas[5]; Richard K. Bogan[6]; Avinash Aggarwal[7]; Jessica Preciado[8]; Kurtis J. Swartz[8]; Jonathan D. Charlesworth[8]

*These authors have contributed equally to the manuscript

1. iSleep Physicians, San Ramon, CA, USA
2. Stanford University School of Medicine, Stanford, CA, USA
3. Clayton Sleep Institute, LLC, St. Louis, MO, USA
4. Ohio Sleep Medicine Institute, Dublin, OH, USA
5. Department of Medicine, Division of pulmonary, Critical Care & Sleep Medicine, Beth Israel Deaconess Medical Center, Boston, MA, USA
6. Bogan Sleep Consultants, LLC, Columbia, SC, USA
7. University of Pittsburgh Medical Center, Department of Neurology, Pittsburgh, PA, USA
8. Noctrix Health, Inc., Pleasanton, CA, USA

Corresponding Author:

Dr. Jonathan D. Charlesworth
Noctrix Health, Inc.
6700 Koll Center Pkwy, Ste 310

Pleasanton, CA 94566

Phone: (804) 683-4279

jcharlesworth@noctrixhealth.com

CLINICAL INVESTIGATIONAL PLAN

A Post-Market Observational Clinical Study to Determine the Long-Term Effectiveness and Safety of the Noctrix TOMAC (Tonic Motor Activation) System for the Treatment of Restless Legs Syndrome – The THRIVE Study

**PROTOCOL NUMBER:**	CT-06
**VERSION:**	7.0
**SPONSOR:**	Noctrix Health, Inc.<br>6700 Koll Center Parkway, Suite 310<br>Pleasanton, CA. 94566<br>804-683-4279
**SPONSOR REPRESENTATIVE(S):**	Jessica Preciado, PhD<br>VP, Clinical Affairs<br><br>Dennell A. Vargas<br>Clinical Manager<br><br>Van Lieou<br>Sr. Clinical Project Manager

Investigator Study Acknowledgement

Read and initial below.

____ I understand this protocol contains information that is confidential and proprietary to Noctrix Health, Inc.

____ Any additional information added to this protocol is also confidential and proprietary to Noctrix Health, Inc. and must be treated in the same manner as the contents of this protocol.

____ I have read the entire protocol.

____ I understand what the protocol asks me to do as an Investigator.

____ I will conduct this study following this protocol and will make a reasonable effort to complete the study in the time noted.

____ I will provide this protocol to study staff under my direct supervision. My study staff will keep the protocol and associated documents confidential.

____ I will discuss this information with the study staff to ensure they are fully informed about the study and the test articles.

____ I will not start enrolling in this study until it is approved by a governing Institutional Review Board.

____ I understand the study may be terminated or enrollment suspended at any time by Noctrix Health, Inc., with or without cause, or by me if it becomes necessary to protect the interests of the study subjects.

____
Name of Investigator

______________________________________ ______________________
Investigator Signature Date

TABLE OF CONTENTS

1. Background 8
2. Regulatory Status 10
3. Objective 11
4. Study Design 11
5. Duration 11
6. Study Population Overview 11
7. Inclusion/Exclusion 11
   7.1. Inclusion criteria (IC) 11
   7.2. Exclusion criteria (EC) 12
8. Device 12
   8.1. Description 12
   8.2. Instructions for Use and Administration 13
9. Risks 14
   9.1. Device Risks 14
10. Adverse Events 14
    10.1. Adverse Event Definitions 15
    10.2. AE Severity and Relatedness 16
    10.3. Adverse Event Reporting 17
11. Device Tracking 18
12. Device Deficiencies and Malfunctions 18
13. Ethical and Regulatory Considerations 18
    13.1. Compliance with Good Clinical Research Practice 18
    13.2. Confidentiality of Data 18
    13.3. Institutional Review Board (IRB) and Informed Consent 19
    13.4. Protocol Compliance 19
    13.5. Protocol Revisions 19
    13.6. Study Monitoring 20
    13.7. Safety Reporting 21
    13.8. Electronic Case Report Forms/Electronic Data Capture 22
    13.9. Quality Assurance Audits 23
    13.10. Records Retention 23
    13.11. Publication and Reporting of Study Results 23
14. Personnel Responsibilities 24

14.1. Investigator responsibilities: 24
14.2. Sponsor Responsibilities 24
15. Investigator Qualifications 25
16. Study Procedures 25
16.1. Overview 25
16.2. Recruitment 25
16.3. Informed consent Process 26
16.4. Screening 26
16.5. Device titration 26
16.6. Baseline Demographics and Assessments 26
16.7. Device training 27
16.8. Study Treatment Description 27
16.9. Concomitant Medications and Treatments 27
16.10. Leg movement data collection 28
16.11. Evaluation and study assessment schedule 28
16.12. Administration of Assessments 31
16.13. Study Exit 32
16.14. Subject Discontinuation 32
16.15. Study Termination 32
17. Statistical Methods 33
17.1. Analysis Populations 33
17.2. Sample Size Justification 35
18. Remote Procedures 35
19. References 36

Study Design Overview Synopsis

**Title**	A Post-Market Observational Clinical Study to Determine the Long-Term Effectiveness and Safety of the Noctrix TOMAC (Tonic Motor Activation) System for the Treatment of Restless Legs Syndrome – The THRIVE Study
**Study Device**	The Tonic Motor Activation (TOMAC) System (either NTX100 or NTX150), a device developed by Noctrix Health, Inc. and cleared by the FDA and is intended to reduce symptoms of primary moderate-severe Restless Legs Syndrome and to improve sleep quality in adults refractory to medications.
**Study Objective**	Assess the long-term effectiveness and safety of the TOMAC System.
**Study Population**	All-comers test design including all commercial RLS patients who consent.
**Study Participation Duration**	1 year with an option to consent to a second year.
**Study Interventions**	TOMAC System (either NTX100 or NTX150) programmed to **ACTIVE** mode
**Study Design**	Patients who meet the eligibility criteria and complete an informed consent will complete the following evaluations in addition to standard of care.<br>• Study entry<br>• Day 90 (where Day 0 is day of titration)<br>• Day 180<br>• Day 270<br>• Day 365 visit<br>Patients who have completed the 365 day visit and are part of the Indicated intent-to-treat (IITT) population will be invited to consent to an additional year in the trial and will complete the following evaluations in addition to standard of care:<br>• Day 455<br>• Day 545<br>• Day 635<br>• Day 730 visit
**Maximum Study Duration**	The study will be completed a maximum of 36 months after the study opens to enrollment.
**Total Number of Subjects**	Up to 325 subjects
**Number of Clinical Sites**	Up to 20 clinical sites within the United States.
**Effectiveness Analysis Populations**	• Indicated intent-to-treat (IITT) – subjects who meet the FDA indications for TOMAC and are exposed to treatment

	• Indicated stable medication (ISM) – subjects in the IITT population who do not change RLS medication during the study
**Primary Effectiveness Endpoint**	Change from baseline in IRLS total score at 1-year.
**Key Secondary Effectiveness Endpoints**	**Endpoint 1:** CGI-I score at 1-year relative to baseline.<br>**Endpoint 2**: PGI-I score at 1-year relative to baseline.<br>**Endpoint 3:** Change from baseline in MOS-II total score at 1-year.<br>**Endpoint 4:** Change from baseline in frequency of RLS symptoms (number of days per week) at 1-year.
**Additional Secondary Efficacy Effectiveness Endpoints**	• CGI-I responder rate, defined as a CGI-I response of "Very Much Improved" or "Much Improved", at 1-year.<br>• CGI-I improvement rate, defined as the percentage of patients with a CGI-I response of "Very Much Improved", "Much Improved" or "Minimally Improved".
**Safety Outcomes**	• Adverse events, classified and tabulated by seriousness, relationship to the device, timing, and severity.
**Additional Study Assessments**	• Objective usage data measured via the TOMAC system<br>• EQ-5D (Quality of Life Questionnaire)<br>• ISI (Insomnia Severity Index)<br>• ESS (Epworth Sleepiness Scale)<br>• ASRS (Augmentation Severity Rating Scale)<br>• GAD-7 (General Anxiety Disorder – 7)<br>• PHQ-8 (Patient Health Questionnaire – 8)<br>• Medication history and use assessment<br>• Subject demographic/characterization assessment<br>• Biweekly follow-up survey<br>• Cognitive Function - Abilities – Short Form 8a<br>• CPAP Use Questionnaire<br>• Clinical Marketing Questionnaire<br>• Iron Panel Blood Test<br>• Primary, secondary and additional outcomes at 2 years for the IITT population.

Abbreviations

AE	Adverse Event

CFR	Code of Federal Regulations
CGI-I	Clinical Global Impressions – Improvement
CRF	Case Report Form
DCF	Data Clarification Form
EDC	Electronic Data Capture
FDA	Food and Drug Administration
GCP	Good Clinical Practice
IRB	Institutional Review Board
IRLS	International Restless Legs Syndrome Study Group Rating Scale
IRLSS	International Restless Legs Syndrome Society
ISO	International Organization for Standardization
MOS	Medical Outcomes Study
NPNS	Non-invasive peripheral nerve stimulation
NSR	Non-Significant Risk
PGI-I	Patient Global Impressions – Improvement
QA	Quality Assurance
RLS	Restless Legs Syndrome
SAE	Serious Adverse Event
TENS	Transcutaneous Electrical Nerve Stimulation
USADE	Unanticipated Serious Adverse Device Effect

1. Background

Patients with Restless Legs Syndrome (RLS) have a strong urge to move their legs and report sensations of tingling or pain, usually in their legs. These patients often present with a primary complaint of not being able to fall asleep regularly. This leads to significant quality of life degradation, depression, daytime sleepiness, lack of productivity and a host of downstream effects associated with lack of quality sleep.

1.1. Restless legs syndrome: Background

Restless legs syndrome (RLS) is a sensorimotor disorder that is characterized by a distressing urge to move the legs and in some cases, other parts of the body such as arms [1]. The diagnosis is made by a response to five hallmark identifying criteria instituted by the International Restless Legs Syndrome Society (IRLSS) [2], as quoted below:

> "1. An urge to move the legs usually but not always accompanied by or felt to be caused by uncomfortable and unpleasant sensations in the legs.
>
> 2. The urge to move the legs and any accompanying unpleasant sensations begin or worsen during periods of rest or inactivity such as lying down or sitting.
>
> 3. The urge to move the legs and any accompanying unpleasant sensations are partially or totally relieved by movement, such as walking or stretching, at least as long as the activity continues.
>
> 4. The urge to move the legs and any accompanying unpleasant sensations during rest or inactivity only occur or are worse in the evening or night than during the day.
>
> 5. The occurrence of the above features are not solely accounted for as symptoms primary to another medical or a behavioral condition (e.g., myalgia, venous stasis, leg edema, arthritis, leg cramps, positional discomfort, habitual foot tapping)."

1.2. Epidemiology

In the United States, RLS is believed to affect more than 10 million adults and an estimated 1.5 million children and adolescents [3]. About one-third of those with RLS symptoms are bothered sufficiently enough to seek medical attention. Epidemiologic studies also show that women are at least 50% more susceptible to RLS than men and RLS is more common in older adults, although it can occur in some as early as the pre-school years.

1.3. Clinical treatments available

The current standard of care involves initial prescription of dopaminergic medications – such as Requip, Mirapex, and Neupro – which initially provide symptomatic relief but often become

ineffective over continued usage [4]. Tolerance to these medications is rapid and well-documented [5]; approximately 10% of patients per year become refractory to these medications, and fewer than 20% patients have sustained benefits lasting 10 years or longer [6]. It is also now understood that dopaminergic medications cause what is known as "augmentation", or paradoxical progressive worsening of RLS symptoms that is much faster than the natural progression of the condition. Due to augmentation, patients on dopaminergic medications require increasingly higher doses [7]. Maximal dosage is limited by an increasing risk of side-effects at higher doses, which include compulsive behaviors including substance abuse, hypersexuality, and gambling [8]. As a result of these downsides of dopaminergic agents, a minority of clinicians are starting to prescribe gabapentinioids (e.g. Horizant) as an alternative first-line of treatment; these medications do not typically lead to augmentation but confer risks such as respiratory depression [9], dizziness, and somnolence during the day.

For the large subpopulation of patients who become refractory to dopaminergic medications – typically due to augmentation – there are limited treatment options. As a result of tolerance, augmentation, and dosage limitations, RLS patients often continue to suffer from moderate-severe RLS symptoms while continuing to be reliant on high doses of dopaminergic medications to provide a small degree of relief. To address the massive unmet need, the leading clinicians involved with RLS advocate prescribing off-label opioids [10]. The leading options – oxycodone and methadone, have well documented risks, which include addiction, dependence, overdose, and occasionally death. This situation is especially concerning because primary RLS typically starts in middle age or earlier and persists throughout life, thus patients may end up reliant on opioids for the final decades of their lives.

Recently, a new option for the treatment of RLS has become available. The Tonic Motor Activation (TOMAC) System was cleared by the FDA in April 2023 and is intended to reduce the symptoms of primary moderate-severe Restless Legs Syndrome and to improve sleep quality in adults refractory to medications. This device is the only FDA-cleared intervention to treat adults who are refractory to RLS medications and suffer from primary moderate-severe RLS.

1.4. Procedure

The device – the Tonic Motor Activation (TOMAC) System – is a non-invasive peripheral nerve stimulation device developed by Noctrix Health, Inc. (Sponsor) and is designed to bilaterally stimulate the common peroneal nerve. Stimulation electrodes are positioned superficially and bilaterally on the

lower legs over the head of the fibula bone, a position where the peroneal nerve is closest to the skin. This nerve target innervates regions of the lower extremities commonly associated with RLS symptoms.

1.5 Rationale

The TOMAC System design is inspired by the temporary symptomatic relief that RLS patients experience during voluntary movement of their lower extremities. One of the fundamental diagnostic criteria for RLS is the fact that patients report short-lived symptomatic relief during voluntary movement of their legs and feet [2]. This is conceptually similar to the gate-control theory mechanism of pain relief, wherein activation of large sensory fibers suppresses pain signals. However, for RLS, it appears that muscle activation is especially effective – activating sensory fibers of the affected region of the body (e.g. via rubbing or touching) does not typically result in similar relief.

Non-invasive electrical stimulation is known to elicit muscle activation – this feature is used by two types of approved medical devices – powered muscle stimulators (21 CFR 890.5850) and external functional neuromuscular stimulators (21 CFR 882.5810). However, these devices tend to have distracting side-effects including paresthesia and phasic muscle twitches, and thus are incompatible with sleep and inappropriate for use in treating a sleep condition such as RLS. In contrast, the TOMAC System produces waveforms that are designed to generate muscle activation without these paresthesia and phasic muscle twitches. Therefore, this technology is compatible with sleep.

The TOMAC System stimulates the peroneal nerve at its most superficial position over to the head of the fibula bone to activate muscles of the lower leg including the tibialis anterior at intensity levels that are comfortable and non-distracting. Such comfortable tonic muscle activation is designed to suppress RLS symptoms in a manner similar to voluntary movement – but unlike voluntary movement, is compatible with sleep.

This study evaluates the long-term effectiveness and safety of the TOMAC System in a real-world population.

2. Regulatory Status

The Tonic Motor Activation (TOMAC) System (either NTX100 or NTX150) is intended to reduce symptoms of primary moderate-severe Restless Legs Syndrome and to improve sleep quality in adults refractory to medications. The device was reviewed and granted De Novo approval by the US Food

and Drug Administration (DEN220059) in April 2023. Based on the characteristics of the device and intended application for which it is to be used, the study has been determined to be Nonsignificant risk (NSR) device per the FDA guidance on Significant Risk and Nonsignificant Risk Medical Device Studies [11].

3. Objective

This non-significant risk study is being performed to assess the long-term effectiveness and safety of the TOMAC System in a real-world population.

4. Study Design

The study will be a post-market, observational study to assess the long-term effectiveness and safety of the TOMAC System. There will be a total of 5 study visits, or 9 study visits for those in the IITT population who choose to consent to a second year in the study, in addition to standard of care. Subjects must agree to comply with the study-specific requirements during the follow-up period of 365 days (or 730 days for those in the IITT population who choose to consent to a second year in the study).

5. Duration

The study will be completed approximately 36 months after the study opens to enrollment.

6. Study Population Overview

The study population will consist of adults who meet the inclusion and exclusion criteria. A maximum of 325 subjects are to be enrolled, at up to (20) clinical sites. Subjects who are a part of the IITT population may consent to participate in a second year in the trial.

7. Inclusion/Exclusion

7.1. Inclusion criteria (IC)

All subjects are required to meet the following inclusion criteria to be considered eligible for participation in this study:

1. The subject possesses the necessary equipment, internet/phone accessibility, and communication ability to complete electronic questionnaires and respond to electronic communications and phone calls from the research staff throughout the in-home portion of the study.

2. The subject has signed a valid, IRB-approved informed consent form, can understand the requirements of the study and instructions for device usage, and can converse in English.
3. Diagnosis of restless legs syndrome.

7.2. Exclusion criteria (EC)

Subjects will be excluded from participating in this study if they meet any of the following exclusion criteria:

1. The subject is unable or unwilling to comply with study requirements.
2. The subject has participated in another TOMAC System trial within the previous 3 months.

8. Device

8.1. Description

The TOMAC System is positioned and worn bilaterally on the legs with stimulation electrodes over the head of the fibula bone, thus targeting the common peroneal nerve – this nerve target innervates regions of the lower extremities commonly associated with RLS symptoms.

For each leg, the TOMAC System consists of one Therapy Unit with a mechanism for attachment to the leg and one Charge Dispersing Interface (CDI), described below.

- The Therapy unit is battery-powered and contains:
  - a rechargeable battery and connector for recharging,
  - a circuit board that generates the stimulation waveform,
  - controls that the subject can use to activate stimulation,
  - controls that the subject can use to adjust the intensity of stimulation within a range programmed by the trained titrator,
  - an electronic connection mechanism to deliver a stimulation waveform to the electrodes,
  - an interface that a trained titrator can use to program the range of stimulation intensities
  - an interface that a researcher can use to download compliance and functionality data.
  - a strap that secures the device to the subject using biocompatible materials and a hook and loop self-fastener.
- The Charge Dispersing Interface (CDI) is:
  - an electrolyte-containing medium or a hydrogel material,

- reusable, allowing for up to 1 week of use by one subject,
- adhered to the skin surface and attached to the Therapy Unit via compression

Description of stimulation parameter ranges allowed by protocol:

- Pulse amplitude: up to 40mA
- Frequency: 2kHz - 6kHz
- Pulse width: 80 - 250 microseconds (depending on frequency)
- Duty cycle: 25-100%
- Pulse shape: Charge-balanced
- Duration: up to 30 minutes per session

8.2. Instructions for Use and Administration

Use of the TOMAC System is described briefly herein. For additional details see the Instructions for Use for either the NTX100 or NTX150 TOMAC System.

8.2.1. Route of administration

The TOMAC System is designed to stimulate the common peroneal nerve; stimulation electrodes within the device are positioned superficially and bilaterally on the lower legs over the head of the fibula bone, a position where the peroneal nerve is close to the skin. This nerve target innervates regions of the lower extremities commonly associated with RLS symptoms.

8.2.2. Dosage and dosage regimen

The dosage regimen depends on the intensity, timing, and duration of stimulation:

1. Intensity. The setpoint stimulation intensity may be the lesser of (a) the maximal intensity that the subject reports is non-distracting and comfortable, as determined in the titration session described in the instructions for device use and (b) the maximal intensity for each set of stimulation parameters, as determined based on device output capabilities. Subjects may be allowed to adjust stimulation intensity from the setpoint stimulation intensity, but only within a limited pre-programmed range. For example, the subject may increase intensity if symptoms are severe or reduce intensity if symptoms are mild.

2. Timing. Subjects will be instructed to administer stimulation after RLS symptoms become noticeable and before the RLS symptoms become severe.

3. Duration. Each session of stimulation can last up to 30 minutes.

8.2.3. Contraindications

The Tonic Motor Activation System (NTX100 TOMAC or NTX150 TOMAC) is contraindicated for use in patients with the following:

- Diagnosis of epilepsy or other seizure disorder,
- Active medical device implant anywhere in the body, including but not limited to pacemakers, spinal cord stimulators, deep brain stimulators
- Metal implant in the leg at the therapy site (not including knee replacements)
- Known allergy to device materials (or severe previous reaction to medical adhesives or bandages)
- Cellulitis, open sores, or injury at or near the location of therapy device application
- The device cannot be used while driving, operating machinery, or during any activity in which electrical stimulation can put the patient at risk of injury

9. Risks

9.1. Device Risks

There is a potential for the following side effects, which are typically mild to moderate and resolve over time:

- Mild skin irritation from use of adhesives.
- Temporary interference with sleep while wearing the device. For some people, the device may be uncomfortable. For others, the device may interfere with preferred sleep positions, thus interfering with sleep during usage.
- You should stop using the device and should consult with a physician if you experience adverse reactions from the device.

Proper use of the device as described in the Instructions for Use (IFU) can help reduce or prevent the following complications:

- Discomfort, paresthesia (tingling or prickling sensation), or otherwise irritating or uncomfortable sensations during treatment. This risk is reduced by adjusting the stimulation intensity.

10. Adverse Events

Adverse events (AEs) occurring during the study will be recorded. Descriptions of AEs will include the date of onset, the date of resolution, the seriousness, the severity, the relationship to study device,

and the outcome. All reported AEs will be summarized by the number of subjects reporting AEs, system organ class (where applicable), seriousness, severity, and relationship to study device.

The study Investigator and Coordinator will evaluate, characterize and record in the electronic Case Report Form (eCRF) all adverse events (AEs) occurring in all subjects from the time of enrollment to study exit (or premature withdrawal). Adverse events unresolved at study exit will be followed by the Investigator until resolution occurs or at least 60 days after the subject's participation in the study is complete. AEs may be reported spontaneously by the subject or detected by the Investigator or Coordinator. AEs should be evaluated for diagnoses not just symptoms (i.e., "angina", not "chest pain").

In addition to verbatim terms, the Sponsor may categorize all AEs using MedDRA preferred terms (PT) and system organ classes (SOC). Analysis may report both verbatim and MedDRA terms.

10.1. Adverse Event Definitions

An **adverse event** (AE) is any untoward medical occurrence, independent of its association with the device. AEs also include any adverse laboratory signs or physical exam findings.

A **serious adverse event** (SAE[1]) is any AE that:

- led to a death,
- led to a serious deterioration in the health of the subject that:
    - resulted in a life-threatening illness or injury,
    - resulted in a permanent impairment of a body structure or a body function,
    - required in-patient hospitalization or prolongation of existing hospitalization,
    - resulted in medical or surgical intervention to prevent permanent impairment to body structure or a body function
- or led to fetal distress, fetal death or a congenital abnormality or birth defect.

A **device-related SAE** is an event meeting the SAE definition above that is also rated as probably or definitely related the device. No device-related SAEs have been reported in prior studies. Note that an elective or pre-planned hospitalization for a condition that did not worsen during the study is not an AE.

[1] Definition from ISO14155:2020

An **unanticipated serious adverse device effect** (USADE) is any SAE that is caused by, or associated with, a device, if that effect, problem, or death was not previously identified in nature, severity, or degree of incidence in the investigational plan or application (including a supplementary plan or application).

10.2. AE Severity and Relatedness

Each AE occurring in the study will be characterized by the study Investigator as to severity (Table 1) and relatedness (Table 2).

Table 1. AE Severity Grading System.

Grade	Brief description
Mild	Asymptomatic or mild symptoms; clinical or diagnostic observations only; intervention not indicated
Moderate	Minimal, local, or non-invasive intervention indicated; limiting age-appropriate instrumental ADL[a]
Severe	Severe or medically significant but not immediately life-threatening; hospitalization or prolongation of hospitalization indicated; disabling; limiting self-care ADL[b]

[a]'Instrumental ADL' refers to activities of daily living such as preparing meals, shopping for groceries or clothes, using the telephone, and managing money. [b]'Self-care ADL' refers to bathing, dressing and undressing, feeding oneself, using the toilet, taking medications, and not being bedridden. From the National Cancer Institute Common Terminology Criteria for Adverse Events v4.0 NCI, NIH, DHHS. May 29, 2009 NIH publication #- 09-7473.

Table 2. AE Relatedness Grading System.*

Relationship of AE to study device	Description
Definite	An event that follows a reasonable temporal sequence from administration of the study device; that follows a known or expected response pattern to the study device; and that is confirmed by improvement on stopping.

Probable	An event that follows a reasonable temporal sequence from administration of the study device; that follows a known or expected response pattern to the study device; and that is unlikely to have been caused by concurrent/underlying illness or other drugs, procedures, or other causes.
Possible	An event that follows a reasonable temporal sequence from administration of the study device; that follows a known or expected response pattern to the study device; but may have been caused by concurrent/underlying illness, drugs, procedure, or other causes.
Unlikely	An event that does not follow a reasonable temporal sequence from administration of the study device; that does not follow a known or expected response pattern to the study device, or most likely was caused by concurrent/underlying illness, drugs, procedure, or other causes, because of their known effects.
Not related	An event almost certainly caused by concurrent/underlying illness, drugs, procedure, or other causes.

** AEs occurring before treatment with the study device will be categorized as unrelated to the study device.*

10.3. Adverse Event Reporting

Investigators must report all SAEs to the study Sponsor and governing IRB within 3 business days or according to local IRB guidelines. Investigators should call the study sponsor immediately upon becoming aware of the occurrence of an SAE. The sponsor will contact the Independent Medical Reviewer, to assist in assessing any safety concerns, if needed. The Investigator should be able and willing to provide further information on the specific event when requested by the study Sponsor. If the Investigator learns of an SAE that occurs within 1 month after the subject completes the study, he/she should notify the Sponsor. Investigators must also report all AEs to the governing IRB as determined by that IRB.

Prompt AE evaluation:

- protects the safety of study subjects;
- aids in understanding the overall safety profile of the device;
- prompts, if necessary, modification to the study protocol
- allows improvements in study design or procedures; and
- adheres with standard good clinical practices.

11. Device Tracking

As part of this post-market study the Subject will receive a TOMAC System by prescription from their doctor (either the NTX100 TOMAC System or NTX150 TOMAC System). The Sponsor will track ordering, shipping, receipt, and disposition of all devices used in this post-market trial in cooperation with the Site staff. The devices will remain the property of the Subject upon study completion.

12. Device Deficiencies and Malfunctions

Throughout the study, the Investigator and study staff will report and document all device deficiencies and malfunctions related to the identity, quality, durability, reliability, safety or performance of the device. This includes reporting of device deficiencies/malfunctions that did not lead to an AE but could have if: 1) suitable action had not been taken, 2) intervention had not been made, or 3) circumstances had been less fortunate. If possible, the Investigator should return devices suspected of deficiency or malfunction to the Sponsor for analysis.

13. Ethical and Regulatory Considerations

13.1. Compliance with Good Clinical Research Practice

This study will be conducted in compliance with the principles of the Belmont Report, the Declaration of Helsinki, with the current Good Clinical Practice (GCP) guidelines and with other applicable regulations. The Investigator and all study staff will conduct the study in compliance with this protocol. Voluntary informed consent will be given by every subject prior to the initiation of any study-related procedures. The rights, safety and well-being of the study subjects are the most important considerations and prevail over the interests of science and society. All personnel involved in the conduct of this study must be qualified by education, training and experience to perform their assigned responsibilities.

13.2. Confidentiality of Data

All information and data sent to the Sponsor, Contract Research Organizations, or the Independent Medical Reviewer concerning subjects or their participation in this study will be considered confidential. All data used in the analysis and reporting of this evaluation will be used in a manner without identifiable reference to the subject. The Investigator consents to visits by the staff of the Sponsor and its authorized representatives and the U.S. Food and Drug Administration or any other governmental body to review the study subjects' medical records

including any test or laboratory data.

13.3. Institutional Review Board (IRB) and Informed Consent

Before study initiation, the Investigator must have written and dated approval from the IRB for the protocol, consent form, subject recruitment materials/process (e.g., advertisements), and any other written information to be provided to subjects. The Investigator should also provide the IRB with a copy of the product labeling, information to be provided to subjects and any updates. The Investigator will submit documentation of the IRB approval to the Sponsor. Copies of all correspondence with the IRB regarding this study must be sent to the Sponsor.

The IRB-approved consent form must include all elements required by FDA, state, and local regulations, and may include appropriate additional elements.

The Investigator/designee will explain the study to each potential subject and the subject must indicate voluntary consent by signing and dating the approved informed consent form. The Investigator must provide the subject with a copy of the consent form in a language the subject understands. The Investigator will maintain documentation that informed consent was obtained prior to the initiation of any study-specific procedures.

Withdrawal of IRB approval of the Investigator's part in the investigation must be reported to the Sponsor within 5 working days.

13.4. Protocol Compliance

The Investigator will comply to the extent possible with the IRB-approved protocol. All deviations from the protocol must be documented. The Investigator will notify the Sponsor immediately if a deviation from the protocol was required to protect subject safety.

13.5. Protocol Revisions

Revisions to the study protocol can be made only by the study Sponsor. A revised protocol can be put into place only after governing IRB approval. All administrative letters must be submitted to the IRB for their information.

New or altered consent forms required by the IRB due to a protocol change must be signed by all subjects currently enrolled in the study and must be used for any subsequent subject enrollment.

13.6. Study Monitoring

Representatives of the Sponsor will visit all study sites (on-site or remote) to perform monitoring and data management functions, and provide participating sites with relevant contact information, as necessary. Study monitors may change periodically over the course of this study. All monitors will be qualified to perform their assigned responsibilities, and participating Investigators/site personnel will be notified of any changes as they occur.

On-site and/or remote monitoring of all participating sites will be frequent enough to assure continued acceptability of the data by assessing site compliance with the study protocol, adherence to data collection procedures, and maintenance of study records. Scheduled site visits will include, but are not limited to, the following:

- Site initiation visit: prior to enrolling subjects, an on-site or remote initiation visit will be conducted by clinical study personnel to review this study protocol and discuss eCRF completion and transmittal procedures. Alternatively, a meeting may be conducted for several sites at a common location.

- Interim monitoring site visit: on-site and/or remote monitoring visits will be conducted at all sites to assess the progress of the study and identify any concerns that result from review of the study records, study management documents, or subject informed consent documents. To assure the integrity of the data, a representative number of individual subject records and other supporting documents will be compared to eCRFs completed at the site to determine that:

    - The study protocol is being followed, and only eligible subjects are being enrolled; variances, if they occur, are recorded and reported as appropriate.
    - Informed consent is properly documented.
    - Adverse events are being reported appropriately.
    - Information recorded on eCRFs is complete, accurate and legible.
    - Subjects failing to complete the clinical study and the reason for failure are properly recorded.

- Final monitoring/Close-out site visit: a final visit to participating sites may be made by the study monitor, if necessary. Any ongoing responsibilities will be discussed with the Investigator and/or site personnel as appropriate.

At the close of the study at an investigational site, appropriately trained personnel appointed by the Sponsor will perform a close-out process via the telephone or on-site. The purpose of this visit is to collect all outstanding study data documents, ensure that the Investigator's files are accurate and complete, review record retention requirements, ensure final accounting of all devices shipped to the Investigator, provide for appropriate disposition of any remaining supplies, and ensure that all applicable requirements are met for the study. The observations and actions made during the intervention will be documented and communicated to the Investigator.

Representatives of government regulatory authorities may also evaluate the study records, source documents, Investigator, study staff and facilities.

The Investigator should immediately notify the Sponsor of any audits of this study by any regulatory agency, and must promptly provide copies of any audit reports.

13.7. Safety Reporting

The Sponsor is responsible for ongoing safety evaluation in this study protocol. Sponsor activities regarding safety include:

- classification of all AEs
- review of all AEs reported in the study
- confirm site’s classification of AEs in terms of severity and relatedness to the study device
- review of severity and relatedness with the study Investigator, especially when there is disagreement between the Investigator and the Sponsor
- review of device deficiencies and malfunctions, including determination and documentation of whether deficiencies/malfunctions could have led to an SAE
- ensuring the reporting of all SAEs and device deficiencies/malfunctions that could have led to an SAE to the IRB and, if required, regulatory authorities in a timely fashion
- informing all site Investigators in writing of all SAEs at all sites in a timely fashion
- updating the risk analysis and assessment of corrective or preventive actions potentially required as a result of new information obtained in the investigation

The Sponsor will evaluate all serious adverse events against US reporting requirements (Medical Device Reporting, 21 CFR 812) and the EU Medical Device Regulations (vigilance incident reporting) as per its standard operating procedures. The Sponsor will investigate each SAE to determine whether the event represents an unanticipated serious adverse device effect (USADE, see Section 7). The Sponsor will report any event to regulatory authorities, Investigators and reviewing IRBs/ECs as necessary. If an investigation shows that a USADE presents an unreasonable risk to subjects, the Sponsor will terminate all investigations or parts of investigations presenting that risk as soon as possible. The Sponsor will only resume a terminated investigation after corrective actions have taken place, site Investigators are informed and IRBs/ECs have been notified and given approval to resume the study.

13.8. Electronic Case Report Forms/Electronic Data Capture

The study will use an electronic data capture (EDC) system to implement electronic case report forms (eCRF). The system will allow compliance with 21 CFR 11 Electronic Signatures. All CRF's are housed in the EDC system. The Investigator and Coordinator will be trained in use of the eCRF prior to study initiation. Retraining in use of EDC can occur at any time. The EDC system will be validated prior to use and after any modification is made. Each eCRF will be designed to accommodate the specific features of the trial design. Modification of a eCRF will only be made if deemed necessary by the study sponsor.

An eCRF is required and should be completed for each included subject. The Investigator has ultimate responsibility for the collection and reporting of all data entered on the eCRFs and any other data collection forms (source documents) and ensuring that they are attributable, legible, timely (contemporaneous), authentic/original, accurate, complete, consistent, enduring and available when required. The eCRFs must be signed by the Investigator to attest that the data contained therein are true.

The site will be provided with general eCRF Completion Guidelines which will assist in data entry and data issues/questions. All persons allowed to enter or change eCRF data must appear on the Delegation of Responsibilities Log.

The Sponsor will remotely monitor the eCRFs to identify possible data errors. The system will have a query mechanism whereby the site Coordinator can respond to Sponsor queries. All data discrepancies will be resolved prior to database lock.

13.9. Quality Assurance Audits

Sponsor representatives or designees may conduct site quality assurance (QA) audits during the study. The Investigator must agree to provide the auditor with direct access to all relevant documents and discuss any findings with the auditor.

In the event of an inspection by the FDA or other regulatory authorities, the Investigator must give the inspector direct access to relevant documents and to discuss any findings with the inspector. The Investigator must notify Sponsor in the event of a FDA site audit.

13.10. Records Retention

The Investigator must maintain all study records (including device disposition, informed consents, source documents, correspondence, regulatory documents, contracts etc.) for at least 2 years after study completion. At the Investigator's discretion, all records may be sent to the Sponsor for permanent storage.

The Investigator must contact the Sponsor or designee prior to destroying any records associated with this study. If the Investigator withdraws from the study, all study-associated records must be transferred to a mutually agreed upon designee. Written notification of such a transfer must be given to the Sponsor or designee.

13.11. Publication and Reporting of Study Results

The study will be registered with clinicaltrials.gov before the first subject is enrolled. Study results will be documented in a study report that will be signed by Sponsor representatives.

If the results of this study will be published, all standard editorial and ethical practices, will be followed. Results from multi-center studies must be published or presented at congresses only in their entirety with data pooled from all centers. Individual Investigators may not publish data from individual centers, unless granted specific written permission from the Sponsor to do so.

The list of authors of any formal publication or presentation of study results may include, as appropriate, representatives of the Sponsor.

14. Personnel Responsibilities

14.1. Investigator responsibilities:

Investigators are responsible for ensuring the investigation is conducted according to all signed agreements, the study protocol, and applicable regulatory agency regulations (21 CFR 812), which include:

a) Permit monitor inspection of facilities and records.
b) Permit FDA and other government health authorities' inspection of facilities and records.
c) Submit protocol and informed consent to IRB and await approval.
d) Submit proposed amendments to protocol and informed consent to IRB and await approval, unless the change reduces the risk to subjects.
e) Obtain informed consent of subjects.
f) Implement study in accordance with protocol.
g) Complete case report forms.
h) Record and explain deviations from protocol and report to monitor.
i) Submit annual progress reports, final reports, and adverse effect reports to IRB and sponsor.
j) Record the receipt, disposition, and return of study devices.
k) Refrain from promoting study or study articles in such a way that the potential subject will be biased in his/her responses.
l) Maintain medical histories of subjects.
m) Retain records for two years following completion of the study.

14.2. Sponsor Responsibilities

Listed below are the Sponsor's responsibilities for this study.

a) Assure IRB approval of protocol and informed consent is obtained.
b) Select and train monitors.
c) Select Investigators.
d) Train Investigators in device use.

e) Obtain Agreement Letter and curriculum vitae and proof of appropriate licensure of Investigator and other study staff.
f) Track shipment of devices.
g) Conduct day-to-day administration of study.
h) Investigate unanticipated, device related adverse effects.
i) Document protocol deviations and violations.
j) Obtain statement of financial disclosure.
k) Will appoint trained and qualified representative(s) to perform initial pre-screening.

15.Investigator Qualifications

Investigators must have an active license and board certification and experience in the treatment of RLS, as documented on their Curriculum Vitae and/or in a statement of the Investigator's relevant experience, including dates, location, extent, and type of experience. Each Investigator must undergo training conducted by the Sponsor, on the study device prior to study initiation.

16.Study Procedures

16.1. Overview

The study consists of 1 year of in-home treatment or 2 years of in-home treatment for subjects in the IITT population who consent to participate in a second year of study. There will be two types of sites:

- "Partially-remote" sites: for these sites, two in-office visits take place: one at the beginning of the study and one at the end of the study.
- "Fully-remote" sites: for these sites, all visits with the site occur remotely.

Subjects will also be required to complete a blood test to assess their iron levels. (Note that subjects will also attend an in-person appointment for device titration, but this visit is a part of standard of care and occurs whether or not a patient is enrolled in this study.) Remote follow-ups – calls and/or video calls – are scheduled at additional timepoints to assess interim response to treatment, as discussed in Section 18 below.

16.2. Recruitment

Among applicants, subject eligibility will be determined based on the inclusion and exclusion criteria discussed above. A pre-screening call may be completed prior to the baseline visit (and thus prior to

consent) to exclude most ineligible applicants prior to the baseline visit. The pre-screening call may be performed by a sponsor-appointed representative.

16.3. Informed consent Process

All subjects must be provided a consent form describing the study with sufficient information for subjects to make an informed decision regarding their participation. Subjects must sign the IRB approved informed consent prior to participation in any study specific procedure, with the exception of pre-screening. The subject must receive a paper or electronic copy of the signed and dated consent document. The signed copy of the consent document must be retained in the study binder in paper or electronic format. The schedule of visits and procedures is provided in Table 3.

16.4. Screening

Potential subjects will be screened for eligibility based on the criteria in Section 7 above. A potential subject who signs the study consent and is eligible will be assigned a study ID number, will be considered enrolled, and will count towards the study's sample size.

16.5. Device titration

During a standard-of-care titration visit, the stimulation intensity of the device will be titrated separately for each leg (left and right), using a commercial titration kit. The titration process is designed to identify the maximally effective settings that are comfortable for each patient. Titration is administered by a trained specialist as part of the standard-of-care for all patients (including those not enrolled in the study) using the TOMAC System.

16.6. Baseline Demographics and Assessments

At study entry, the following information will be collected:

- Demographics
- Medical History including RLS History and Concomitant Medications
- Refractory Categorization
- CPAP User Questionnaire

The following assessments will also be completed at the baseline visit:

- International Restless Legs Syndrome Score [12]
- MOS-Sleep (Medical Outcomes Study Sleep Scale) [13]

- Augmentation Severity Rating Scale
- EQ-5D-5L (Health-related Quality of Life Survey)
- Insomnia Severity Index (ISI)
- Epworth Sleepiness Scale (ESS)
- GAD-7 (General Anxiety Disorder – 7)
- PHQ-8 (Patient Health Questionnaire – 8)
- Cognitive Function - Abilities – Short Form 8a

The following information will be collected in the 4 weeks following the baseline visit:

- Iron panel blood test

If the device titration takes place 14 days after the baseline assessments visit, then the IRLS and MOS-Sleep, EQ-5D-5L, ISI, ESS, GAD-7 and PHQ-8 must be administered again. Changes to Concomitant medications and AEs should also be reassessed.

16.7. Device training

All patients (whether enrolled in the trial or not) are trained on proper usage of the TOMAC System, including positioning of the devices on their legs. During this standard-of-care visit, a body marking pen may be used to mark anatomical locations for device placement. Device titration may be adjusted during device training based on subjective feedback from the patient.

16.8. Study Treatment Description

Study subjects are instructed to self-administer treatment nightly as needed – typically after RLS symptoms start and before RLS symptoms become severe. The instructions for timing of device use may be adjusted based on the timing of RLS symptoms experienced by the subject. Once activated, a single session of treatment may run for approximately 30 minutes and then turn off automatically. If device battery life limits the number of uses per night, the following two timings of use may be prioritized: (1) at bedtime, to reduce symptoms and thus help with sleep initiation, and (2) when waking up in the middle of the night, to reduce symptoms and thus help with sleep re-initiation. Device intensity is typically set to the titrated levels, but intensity may be increased if RLS symptoms are especially severe or decreased such as if RLS symptoms are mild.

16.9. Concomitant Medications and Treatments

Concomitant medications and treatments are tracked throughout the study based on subject reported data.

16.10. Leg movement data collection

The TOMAC System may have the capability to collect data on leg movements, such as by using accelerometers and/or gyroscope sensors, while the subject is wearing the devices. These data may be collected during this study and used for subsequent analysis and development.

16.11. Evaluation and study assessment schedule

The following evaluation calls and/or visits are scheduled, as outlined in **Table 3** below. A window of +/- 4 days may be utilized on office follow-ups and a window of +/- 4 days for any phone follow-up, as needed.

Evaluation 1: Visit for enrollment, including consent, screening, device titration, assessments, and training on device usage. For "partially remote" sites this visit will take place in the office. For "fully-remote" sites this visit will take place via phone and/or video call. This may involve a single visit or multiple visits.

Titration Visit: This is a standard-of-care visit for any patient using the TOMAC System.

Blood Test Visit: This visit should occur within 4 weeks of the baseline visit.

Evaluation 2: (90-days after Titration) Phone follow-up to assess TOMAC System use, assessments, and assess the occurrence of adverse events and/or changes to daily medications.

Evaluation 3: (180-days after Titration) Phone follow-up to assess TOMAC System use, assessments, and assess the occurrence of adverse events and/or changes to daily medications.

Evaluation 4: (270-days after Titration) Phone follow-up to assess TOMAC System use, assessments, and assess the occurrence of adverse events and/or changes to daily medications.

Evaluation 5: (365-days after Titration) Visit to assess TOMAC System use, assessments, and assess the occurrence of adverse events and/or changes to daily medications. For "partially remote" sites this visit will take place in the office. For "fully-remote" sites this visit will take place via phone and/or video call.

Evaluation 1 and Evaluation 5 occur in person at "partially-remote" sites and occur via phone and/or video call at "fully-remote" sites. All other Evaluations are designed to be remote interactions (calls or video calls).

For subjects in the IITT population who consent to a second year of participation in the study the following evaluation calls and/or visits are scheduled, as outlined in **Table 3** below. A window of +/- 4 days may be utilized on office follow-ups and a window of +/- 4 days for any phone follow-up, as needed.

Evaluation 6: (455-days after Titration) Phone follow-up to assess TOMAC System use, assessments, and assess the occurrence of adverse events and/or changes to daily medications.

Evaluation 7: (545-days after Titration) Phone follow-up to assess TOMAC System use, assessments, and assess the occurrence of adverse events and/or changes to daily medications.

Evaluation 8: (635-days after Titration) Phone follow-up to assess TOMAC System use, assessments, and assess the occurrence of adverse events and/or changes to daily medications.

Evaluation 9: (730-days after Titration) Visit to assess TOMAC System use, assessments, and assess the occurrence of adverse events and/or changes to daily medications. For "partially remote" sites this visit will take place in the office. For "fully-remote" sites this visit will take place via phone and/or video call.

The schedule of assessments are shown in **Table 3** below. In addition to these assessments, subjects will complete a biweekly survey delivered via electronic means (text or email) throughout the 52-wk study duration. For subjects in the IITT population who consent to a second year in the study, a monthly survey will be delivered via electronic means (text or email) from week 52 to week 104 in the study. Additionally, the devices may automatically log quantitative compliance, usage data, and leg movement data throughout the duration of the study.

TABLE 3: SCHEDULE OF ASSESSMENTS

DATA	**Eval 1 Study Entry Screening** *Remote*	**Titration Visit* Day 0**** *In-office*	**Eval 2 Day 90** *Remote*	**Eval 3 Day 180** *Remote*	**Eval 4 Day 270** *Remote*	**Eval 5 Day 365** *Remote*	Biweekly Texts (26 texts)	**Eval 6 Day 455** *Remote*	**Eval 7 Day 545** *Remote*	**Eval 8 Day 635** *Remote*	**Eval 9 Day 730** *Remote*	Monthly Texts (12 texts)
Screening	x											
Informed Consent	x											
Inclusion/Exclusion	x											
Med Hx/Demographics	x											
ConMeds (Initial, changes)	x		x	x	x	x	x	x	x	x	x	x
RLS History	x											
Refractory categorization	x											
Device titration		x										
AEs			x	x	x	x	x	x	x	x	x	x
Pivotal Trial Matched	x											
IITT Population Questionnaire			x									
Iron Panel Testing Results			x									
Follow-up Questionnaire		x	x	x	x	x		x	x	x	x	
CGI-I			x	x	x	x		x	x	x	x	
PGI-I			x	x	x	x		x	x	x	x	
IRLS	x		x	x	x	x		x	x	x	x	
MOS-SLEEP	x		x	x	x	x		x	x	x	x	
Augmentation Severity Rating Scale	x					x						
EQ-5D-5L	x			x		x					x	
Insomnia Severity Index (ISI)	x			x		x						
Epworth Sleepiness Scale (ESS)	x			x		x					x	
GAD-7	x		x	x	x	x					x	
PHQ-8	x		x	x	x	x					x	
Cognitive Function - Abilities – Short Form 8a	x		x	x		x					x	
Device usage evaluation			x	x	x	x					x	
CPAP User Questionnaire	x					x					x	
Clinical Marketing Questions				x		x					x	

** All patients prescribed the TOMAC System complete a titration visit (not just study subjects.)*

*** Repeat baseline assessments if titration is scheduled more than 14 days after Eval 1 .*

16.12. Administration of Assessments

- **Demographics and Medical History** will be completed by the subject or the site staff based on information from the subject.
- **Refractory Categorization.** The refractory categorization form should be completed by the Investigator, within 72 hours of Evaluation 1.
- **PGI-I** (Patient Global Impressions – Improvement Scale). The subject and not the site staff should make the ratings, but the staff should be available to clarify the meaning of the questions for the subject.
- **CGI-I** (Clinical Global Impressions – Improvement Scale). The CGI-I is completed by the Investigator after conducting a short interview with the subject regarding their RLS symptoms over the past week relative to baseline. The Investigator will not be permitted access to the subject's IRLS responses.
- **IRLS.** The subject and not the site staff should make the ratings, but the staff should be available to clarify the meaning of the questions for the subject.
- **MOS-Sleep.** The subject and not the site staff should make the ratings, but the staff should be available to clarify the meaning of the questions for the subject.
- **Augmentation Severity Rating Scale.** The subject and not the site staff should make the ratings, but the staff should be available to clarify the meaning of the questions for the subject.
- **EQ-5D-5L.** The subject and not the site staff should make the ratings, but the staff should be available to clarify the meaning of the questions for the subject.
- **Insomnia Severity Index (ISI).** The subject and not the site staff should make the ratings, but the staff should be available to clarify the meaning of the questions for the subject.
- **Epworth Sleepiness Scale (ESS).** The subject and not the site staff should make the ratings, but the staff should be available to clarify the meaning of the questions for the subject.
- **GAD-7.** The subject and not the site staff should make the ratings, but the staff should be available to clarify the meaning of the questions for the subject.
- **PHQ-8.** The subject and not the site staff should make the ratings, but the staff should be available to clarify the meaning of the questions for the subject.
- **Cognitive Function - Abilities – Short Form 8a.** The subject and not the site staff should make the ratings, but the staff should be available to clarify the meaning of the questions for the subject.
- **CPAP User Questionnaire** will be completed by the subject or the site staff based on information from the subject.
- **Clinical Marketing Questions** will be completed by the subject or the site staff based on information from the subject.
- **Iron Panel Blood Test** will be completed by the subject and the site staff will enter the data based on the source document test results.

- The **Biweekly Texts** will be completed by the subject.

16.13. Study Exit

Study exit will occur at the conclusion of Evaluation 5, or at the conclusion of Evaluation 9 if they have consented to a second year of evaluations. At study exit, the Investigator/coordinator will complete the study exit eCRF. Adverse events unresolved at study exit will be followed by the Investigator until resolution occurs or at least 60 days after the subject's participation in the study is complete.

16.14. Subject Discontinuation

A subject may be removed from the study prior to completion for any of the following reasons:

- Voluntary withdrawal of consent
- Adverse event preventing further study participation
- Investigator believes risk of further subject participation outweighs benefit
- Persistent non-compliance or lost to follow-up
- Pregnancy
- Subject no longer meets inclusion criteria

The Investigator or research staff will complete a study exit form in the eCRF for any subject who prematurely discontinues from the study. If discontinuation was the result of an AE, the AE will also be recorded in the eCRF.

Upon discontinuation, subjects will receive partial compensation commensurate with their completed Phases.

16.15. Study Termination

The Sponsor may terminate the study as a whole or at individual study sites under the following circumstances:

- Suspicion of risk to subjects, including occurrence of high rate of known AEs or unexpectedly high rate of unexpected AEs
- Poor site compliance with the study protocol
- Inadequate site enrollment
- Obtaining new scientific knowledge that shows that the study is no longer valid or necessary
- Persistent non-compliance with IRB or regulatory requirements
- Persistent failure to comply with obligations arising from the clinical trial agreement
- Other business reasons (e.g., insolvencies or business entity liquidation)

The sponsor will document reasons for study suspension and notify relevant site Investigators and governing IRBs. If suspension occurred because of a safety issue, all Investigators will be notified. When terminating the study, the sponsor and Investigator will assure that adequate consideration is given to the protection of the subjects' interests.

17. Statistical Methods

Refer to the latest release version of the Statistical Analysis Plan (SAP) for the plan for the effectiveness, safety and poolability analyses. This document also details the plans for interim analyses.

17.1. Analysis Populations

17.1.1. Indicated Intent-to-Treat (IITT) Population

The Indicated Intent-to-Treat Population will consist of patients who meet the FDA indications for use at study entry and are exposed to therapy. Full criteria are as follows:

a. Adults aged 22 or above
b. Confirmed diagnosis of primary RLS based on International RLS Study Group diagnostic criteria [2]
c. Medication-refractory
d. IRLS total score ≥ 15
e. Patient receives titrated TOMAC therapy unit within 2-weeks of Eval 1 completion
f. Patient is exposed to at least one TOMAC therapy session
g. No changes to RLS medication or dosage in 30-days prior to Eval 1 completion
h. Patient has none of the FDA contraindications for TOMAC
    - Diagnosis of epilepsy or other seizure disorder;
    - Active medical device implant anywhere in the body, including but not limited to pacemakers, spinal cord stimulators, deep brain stimulators;
    - Metal implant in the leg at the site of study device electrode application (knee replacements are typically not a contraindication);
    - Known allergy to device materials (or severe previous reaction to medical adhesives or bandages);
    - Cellulitis, open sores, or acute injury at or near the location of device electrode application)

The IITT Population will be used as the first of two co-primary analysis populations for all effectiveness endpoints.

17.1.2. Primary Safety Analysis Population

The Primary Safety Analysis Population will consist of all patients in the IITT Population. The Primary Safety Analysis Population will be used as the primary analysis population for all safety analyses.

17.1.3. Secondary Safety Analysis Population

The Secondary Safety Analysis Population will consist of all patients who are exposed to therapy (including titration). The Secondary Safety Analysis Population will be used for a secondary analysis of safety.

17.1.4. Indicated Stable Medication (ISM) Population

The ISM Population will consist of all patients in the IITT population who also meet the following requirement:

a. No changes to RLS medication or dosage at Day 365 compared to Eval 1.
b. For patients on a stable RLS medication regimen that includes "as needed" doses, no clinically significant changes to the frequency/amount of "as needed" doses at Day 365 compared to Day 1. An expert panel blinded to the subject's efficacy/safety outcomes will evaluate if such changes are clinically significant based on expectation of whether it would be expected to affect any primary or key secondary endpoints at Day 365 compared to Eval 1.

The ISM Population will be used as the second of two co-primary analysis populations for all effectiveness endpoints.

A safety analysis of the ISM population will be conducted.

17.1.5. Pivotal Trial Matched (PTM) Population

The PTM population will consist of all patients in the IITT population who also meet all eligibility criteria for enrollment to the RESTFUL Study (NCT04874155). This is an exploratory effectiveness population. For each of the primary, secondary, and exploratory effectiveness endpoints, means and 95% CIs will be presented for this population, but hypothesis testing will not be conducted.

17.1.6. Medication-naïve (MN) Population

All enrolled patients who meet the following requirements will be included in the MN population:

a. Adults aged 22 or above

b. Confirmed diagnosis of RLS based on International RLS Study Group diagnostic criteria [2]
c. No prior treatment with prescription RLS medication at time of study entry.
d. IRLS total score ≥ 15
e. Patient receives titrated TOMAC therapy unit within 2-weeks of Eval 1 completion
f. Patient is exposed to at least one TOMAC therapy session

This is also an exploratory effectiveness population. For each of the primary, secondary, and exploratory effectiveness endpoints, means and 95% CIs will be presented for this population, but hypothesis testing will not be conducted.

17.1.7. Per Protocol (PP) Population

The per protocol population will consist of all patients in the IITT population who completed the study through 1-year and had at least 1 session of TOMAC therapy during each month.

17.2. Sample Size Justification

Sample size calculations were performed for the primary endpoint, reduction in IRLS total score from baseline after one year, based on a one-sample t-test at the one-sided alpha = 0.0125 level (Bonferroni-adjusted to account for the two analysis populations). The "true" mean and standard deviation (SD) were assumed to be the same as the observed mean (SD) reduction in IRLS at the end of the pivotal CT-04 trial (the RESTFUL study), or 8.7 (6.3). Based on these assumptions, the required sample size is only N=10 participants. A total of up to N=325 participants will be enrolled to increase the precision of estimates for the primary and secondary effectiveness endpoints. For example, the two-sided 97.5% confidence interval (CI) for the mean primary effectiveness endpoint estimate based on N=325 participants and an observed SD of 6.3 would have a half-width of ± 0.8.

18.Remote Procedures

During and after the COVID-19 pandemic, replacing some in-person visits with remote interactions may be necessary, for example to reduce risk to subjects and/or to comply with local, state, or federal regulations. In such cases, the study records will indicate which in-person visits were replaced with remote interactions.

In such cases, one of more of the following approaches may be taken:

1. Remote follow-up visits. Remote follow-up visits, where possible, will be completed via call or video call, during which the required study assessments will be administered.
2. Remote consent. Remote consent procedures will follow the recommendations set forth in the "FDA Guidance on Conduct of Clinical Trials of Medical Products during COVID-19 Public Health Emergency" [14]. For example, or more of the following procedures listed in the guidance will be used to confirm consent:
    a. eConsent via a compliant digital platform;
    b. confirmation of consent by an impartial witness via three-way call or video conference;
    c. remote explanation of consent followed by in-person signature of informed consent document (to minimize duration of in-person interaction).

19. References

1. Allen, R.P. et al. Restless legs syndrome: diagnostic criteria, special considerations, and epidemiology. A report from the restless legs syndrome diagnosis and epidemiology workshop at the National Institutes of Health. Sleep Med. (2003). doi: 10.1016/s1389-9457(03)00010-8
2. Allen RP, Picchietti DL, Garcia-Borreguero D, et al. .; International Restless Legs Syndrome Study Group. Restless legs syndrome/Willis-Ekbom disease diagnostic criteria: updated International Restless Legs Syndrome Study Group (IRLSSG) consensus criteria—history, rationale, description, and significance. Sleep Med. 2014;15(8):860–873. doi:10.1016/j.sleep.2014.03.025
3. Allen, R. P. *et al.* Restless legs syndrome prevalence and impact: REST general population study. *Arch. Intern. Med.* (2005). doi:10.1001/archinte.165.11.1286
4. Silber, M.H. Treatment of restless legs syndrome and periodic limb movement disorder in adults. In: UpToDate, Post, TW (Ed), UpToDate, Waltham, MA, 2020
5. García-Borreguero, D. & Williams, A. M. Dopaminergic augmentation of restless legs syndrome. *Sleep Medicine Reviews* (2010). doi:10.1016/j.smrv.2009.11.006
6. Lipford, M.C. and Silber, M.H. Long-term use of pramipexole in the management of restless legs syndrome. Sleep Med. 2012; 13: 1280–1285
7. Hening, W.A. et al. An update on the dopaminergic treatment of restless legs syndrome and periodic limb movement disorder. Sleep (2004). doi: 10.1093/sleep/27.3.560
8. Cornelius, J.R, Tippmann-Peikert, M., Slocumb, N.L., Frerichs, C.F., Silber, M.H. Impulse control disorders with the use of dopaminergic agents in restless legs syndrome: a case-control study. Sleep. 2010 Jan;33(1):81-7.
9. FDA Drug Safety Communication 12-19-2019. Available at: https://www.fda.gov/drugs/drug-safety-and-availability/fda-warns-about-serious-breathing-problems-seizure-and-nerve-pain-medicines-gabapentin-neurontin.
10. Silber, M. H. *et al.* The Appropriate Use of Opioids in the Treatment of Refractory Restless Legs Syndrome. *Mayo Clin. Proc.* (2018). doi:10.1016/j.mayocp.2017.11.007
11. FDA Center for Devices and Radiological Health Guidance Jan 2006. Information sheet guidance for IRBs, clinical investigators, and sponsors: Significant Risk and Nonsignificant risk medical device studies. Available at: http://www.fda.gov/downloads/RegulatoryInformation/Guidances/UCM126418.pdf
12. Walters AS, LeBrocq C, Dhar A, et al. Validation of the International Restless Legs Syndrome Study Group rating scale for restless legs syndrome. *Sleep Med*. 2003;4(2):121-132. doi:10.1016/s1389-9457(02)00258-7
13. Hays, R. D., & Stewart, A. L. (1992). Sleep measures. In A. L. Stewart & J. E. Ware (eds.), Measuring functioning and well-being: The Medical Outcomes Study approach (pp. 235-259), Durham, NC: Duke

University Press.
14. FDA Guidance on Conduct of Clinical Trials of Medical Products during COVID-19 Public Health Emergency. Available at: https://www.fda.gov/media/136238/download
