# Supplementary material for: Real-world effectiveness and safety of tonic motor activation therapy for refractory restless legs syndrome: 90-day outcomes from the THRIVE prospective observational study
Source: Sleep. 2026 Jun 3;49(9):zsag140. doi: 10.1093/sleep/zsag140 (PMC13553290; doi:10.1093/sleep/zsag140)
Supplement: Supplementary file 2 [file esap2_zsag140.pdf]

Real-world effectiveness and safety of tonic motor activation therapy for refractory restless legs syndrome: 90-day outcomes from the THRIVE prospective observational study

Haramandeep Singh*[1]; Mark J. Buchfuhrer*[2]; Joseph Ojile[3]; Asim Roy[4]; Robert Joseph Thomas[5]; Richard K. Bogan[6]; Avinash Aggarwal[7]; Jessica Preciado[8]; Kurtis J. Swartz[8]; Jonathan D. Charlesworth[8]

*These authors have contributed equally to the manuscript

1. iSleep Physicians, San Ramon, CA, USA
2. Stanford University School of Medicine, Stanford, CA, USA
3. Clayton Sleep Institute, LLC, St. Louis, MO, USA
4. Ohio Sleep Medicine Institute, Dublin, OH, USA
5. Department of Medicine, Division of pulmonary, Critical Care & Sleep Medicine, Beth Israel Deaconess Medical Center, Boston, MA, USA
6. Bogan Sleep Consultants, LLC, Columbia, SC, USA
7. University of Pittsburgh Medical Center, Department of Neurology, Pittsburgh, PA, USA
8. Noctrix Health, Inc., Pleasanton, CA, USA

Corresponding Author:

Dr. Jonathan D. Charlesworth
Noctrix Health, Inc.
6700 Koll Center Pkwy, Ste 310

Pleasanton, CA 94566

Phone: (804) 683-4279

jcharlesworth@noctrixhealth.com

STATISTICAL ANALYSIS PLAN (v3.0)

Protocol Number: CT-06 (v7.0)

A Post-Market Observational Clinical Study to Determine the Long-Term Effectiveness and Safety of the Noctrix NTX100 TOMAC (Tonic Motor Activation) System for the Treatment of Restless Legs Syndrome – The THRIVE Study

October 16, 2024

Prepared for
Noctrix Health, Inc.
6700 Koll Center Parkway, Suite 310
Pleasanton, CA 94566

Prepared by
Leavitt Morrison, M.S.
Senior Manager, Biostatistics

Reviewed by
Kimberly Walters, Ph.D.
Director, Biostatistics

Biostatistical Consulting Inc.
1 Hartwell Place
Lexington, MA 02421

Appendix C added November 17, 2025 by Kurtis Swartz

Signature Page for Analysis Plan

Sponsor: Noctrix Health, Inc.

Study Number: CT-06

Protocol Title: A Post-Market Observational Clinical Study to Determine the Long-Term Effectiveness and Safety of the Noctrix NTX100 TOMAC (Tonic Motor Activation) System for the Treatment of Restless Legs Syndrome – The THRIVE Study

Prepared by:<br>Leavitt Morrison, M.S.<br>Senior Manager, Biostatistics	Signature	Date
Reviewed by:<br>Kimberly Walters, Ph.D.<br>Director, Biostatistics	Signature	Date

Biostatistical Consulting Inc.
1 Hartwell Place
Lexington, MA, 02421

Sponsor:

Jonathan Charlesworth, Ph.D.<br>Vice President, Clinical Research	Signature	Date

Noctrix Health, Inc.
6700 Koll Center Parkway, Suite 310
Pleasanton, CA 94566

Table of Contents

Signature Page for Analysis Plan 2
Table of Contents 3
List of Tables 5
List of Listings 7
List of Abbreviations 8
1.0 INTRODUCTION 9
2.0 STUDY OBJECTIVE 11
3.0 STUDY DESIGN 12
3.1 Overview 12
3.2 Method of Assigning Subjects to Treatment 14
3.3 Blinding 14
3.4 Determination of Sample Size 14
3.5 Changes to the Protocol-Specified Analyses 14
3.6 Changes from Previous Version of the SAP 14
4.0 EFFECTIVENESS AND SAFETY ENDPOINTS 15
4.1 Primary Effectiveness Endpoint 15
4.2 Key Secondary Effectiveness Endpoints 15
4.3 Additional Secondary Effectiveness Endpoints 15
4.4 Exploratory Effectiveness Endpoints 15
4.5. Safety Outcomes 15
5.0 STATISTICAL CONSIDERATIONS 16
5.1 General Methodology 16
5.2 Adjustments for Covariates 16
5.3 Handling of Dropouts and Missing Data 16
5.4 Interim Analyses 17
5.4.1 First Interim Analysis 17
5.4.2 Second Interim Analysis 18
5.4.3 Data Analyses for Publication 18
5.5 Multicenter Study 18
5.6 Multiple Comparisons / Multiplicity 18
5.7 Examination of Subgroups 18
6.0 ANALYSIS POPULATIONS 19
6.1 Indicated Intent-to-Treat (IITT) Population 19
6.2 Indicated Stable Medication (ISM) Population 19
6.3 Pivotal Trial Matched (PTM) Population 20

6.4 Medication-naïve (MN) Population ... 20
6.5 Primary Safety Analysis Population ... 20
6.6 Secondary Safety Analysis Population ... 20
6.7 Per Protocol (PP) Population ... 20
7.0 SUBJECT DISPOSITION ... 21
8.0 DEMOGRAPHICS AND BASELINE CHARACTERISTICS ... 22
9.0 PROTOCOL DEVIATIONS ... 23
10.0 EFFECTIVENESS AND SAFETY ANALYSES ... 24
10.1 Primary Effectiveness Endpoint Analysis ... 24
10.2 Secondary Effectiveness Endpoints Analyses ... 24
10.3 Exploratory Effectiveness Endpoints Analyses ... 26
10.4 Safety Analyses ... 26
10.4.1 Adverse Events (AEs) ... 26
10.4.2 RLS Medication Changes ... 26
10.4.3 Concomitant Medications ... 26
11.0 OTHER ANALYSES ... 28
12.0 REFERENCES ... 29

Appendix A: TABLE SHELLS
Appendix B: LISTING SHELLS
Appendix C: Adjustments for 90-day analysis publication

List of Tables

Number	Title
1.1.1	Primary Effectiveness Endpoint (IITT Population)
1.2.1	Primary Effectiveness Endpoint (Indicated Stable Medication Population)
1.3.1	Key Secondary Effectiveness Endpoints (IITT Population)
1.4.1	Key Secondary Effectiveness Endpoints (Indicated Stable Medication Population)
1.5.1	Key Secondary Effectiveness Endpoints through 1 Year (IITT Population)
1.6.1	Key Secondary Effectiveness Endpoints through 1 Year (Indicated Stable Medication Population)
1.7.1	Additional Secondary Effectiveness Endpoints (IITT Population)
1.8.1	Additional Secondary Effectiveness Endpoints (Indicated Stable Medication Population)
2.1.1.1	Adverse Events (AEs) Overview (Primary Safety Analysis Population)
2.1.2.1	Adverse Events (AEs) Overview (Secondary Safety Analysis Population)
2.1.3.1	Adverse Events (AEs) Overview (Indicated Stable Medication Population)
2.2.1.1	Adverse Events (AEs): Incidence by System Organ Class and Preferred Term (Subject Level) (Primary Safety Analysis Population)
2.2.2.1	Adverse Events (AEs): Incidence by System Organ Class and Preferred Term (Subject Level) (Secondary Safety Analysis Population)
2.2.3.1	Adverse Events (AEs): Incidence by System Organ Class and Preferred Term (Subject Level) (Indicated Stable Medication Population)
2.3.1.1	Adverse Events (AEs) by System Organ Class/Preferred Term and Seriousness (Event Level) (Primary Safety Analysis Population)
2.3.2.1	Adverse Events (AEs) by System Organ Class/Preferred Term and Seriousness (Event Level) (Secondary Safety Analysis Population)
2.3.3.1	Adverse Events (AEs) by System Organ Class/Preferred Term and Seriousness (Event Level) (Indicated Stable Medication Population)
2.4.1.1	Adverse Events (AEs) by System Organ Class/Preferred Term and Severity (Event Level) (Primary Safety Analysis Population)
2.4.2.1	Adverse Events (AEs) by System Organ Class/Preferred Term and Severity (Event Level) (Secondary Safety Analysis Population)
2.4.3.1	Adverse Events (AEs) by System Organ Class/Preferred Term and Severity (Event Level) (Indicated Stable Medication Population)
2.5.1.1	Adverse Events (AEs) by System Organ Class/Preferred Term and Relationship to the Device (Event Level) (Primary Safety Analysis Population)
2.5.2.1	Adverse Events (AEs) by System Organ Class/Preferred Term and Relationship to the Device (Event Level) (Secondary Safety Analysis Population)
2.5.3.1	Adverse Events (AEs) by System Organ Class/Preferred Term and Relationship to the Device (Event Level) (Indicated Stable Medication Population)
2.6.1.1	Adverse Events (AEs) by System Organ Class/Preferred Term and Time Period (Event Level) (Primary Safety Analysis Population)
2.6.2.1	Adverse Events (AEs) by System Organ Class/Preferred Term and Time Period (Event Level) (Secondary Safety Analysis Population)

Number	Title
2.6.3.1	Adverse Events (AEs) by System Organ Class/Preferred Term and Time Period (Event Level) (Indicated Stable Medication Population)
3.1.1.1	Concomitant Medications (Primary Safety Analysis Population)
3.1.2.1	Concomitant Medications (Secondary Safety Analysis Population)
3.1.3.1	Concomitant Medications (Indicated Stable Medication Population)
4.1.1.1	RLS Medication Changes (Primary Safety Analysis Population)
4.1.2.1	RLS Medication Changes (Secondary Safety Analysis Population)
4.1.3.1	RLS Medication Changes (Indicated Stable Medication Population)

List of Listings

Number	Title
1.1	Informed Consent and Analysis Populations (Enrolled Subjects)
2.1.1	Clinical Global Impressions – Improvement Scale (CGI-I) (Enrolled Subjects)
2.2.1	Patient Global Impressions – Improvement Scale (PGI-I) (Enrolled Subjects)
2.3.1	International Restless Legs Syndrome (IRLS) (Enrolled Subjects)
2.4.1	Medical Outcomes Study Sleep Scale (MOS-Sleep) (Enrolled Subjects)
3.1.1	Prior and Concomitant Medications (Enrolled Subjects)
3.2.1	RLS Medication Log (Enrolled Subjects)
4.1	Adverse Events (AEs) (Enrolled Subjects)

List of Abbreviations

Abbreviation	Definition
AE	Adverse Event
ASRS	Augmentation Severity Rating Scale
ATC	Anatomical Therapeutic Chemical
CGI-I	Clinical Global Impressions – Improvement
CI	Confidence Interval
CPAP	Continuous Positive Airways Pressure
ESS	Epworth Sleepiness Scale
FDA	Food and Drug Administration
GAD	General Anxiety Disorder
IITT	Indicated Intent-to-Treat
IRLS	International Restless Legs Syndrome Study Group Rating Scale
IRLSS	International Restless Legs Syndrome Society
ISI	Insomnia Severity Index
ISM	Indicated Stable Medication
MCMC	Markov Chain Monte Carlo
MedDRA	Medical Dictionary for Regulatory Activities
MI	Multiple Imputation
MMRM	Mixed Model Repeated Measures
MN	Medication-naive
MOS	Medical Outcomes Study
PGI-I	Patient Global Impressions – Improvement
PHQ	Patient Health Questionnaire
PP	Per Protocol
PT	Preferred Term
PTM	Pivotal Trial Matched
RLS	Restless Legs Syndrome
SAP	Statistical Analysis Plan
SD	Standard Deviation
SOC	System Organ Class
TOMAC	Tonic Motor Activation
WHODRUG	World Health Organization Drug Dictionary

1.0 INTRODUCTION

This Statistical Analysis Plan (SAP) document details the analysis plan for the study entitled "A Post-Market Observational Clinical Study to Determine the Long-Term Effectiveness and Safety of the Noctrix NTX100 TOMAC (Tonic Motor Activation) System for the Treatment of Restless Legs Syndrome – The THRIVE Study". It describes the proposed effectiveness and safety analyses, including planned summary tables and by-subject data listings.

The focus of this SAP is on the description of primary analyses through 1-year follow-up. Analyses of data collected during extended follow-up are also described generally.

Patients with Restless Legs Syndrome (RLS) have a strong urge to move their legs and report sensations of tingling or pain, usually in their legs. These patients often present with a primary complaint of not being able to fall asleep regularly. This leads to significant quality of life degradation, depression, daytime sleepiness, lack of productivity, and a host of downstream effects associated with lack of quality sleep.

RLS is a sensorimotor disorder that is characterized by a distressing urge to move the legs and, in some cases, other parts of the body such as arms [1]. The diagnosis is made by a response to five hallmark identifying criteria instituted by the International Restless Legs Syndrome Society (IRLSS) [2], as quoted below:

> "1. An urge to move the legs, usually but not always accompanied by or felt to be caused by uncomfortable and unpleasant sensations in the legs.
>
> 2. The urge to move the legs and any accompanying unpleasant sensations begin or worsen during periods of rest or inactivity such as lying down or sitting.
>
> 3. The urge to move the legs and any accompanying unpleasant sensations are partially or totally relieved by movement, such as walking or stretching, at least as long as the activity continues.
>
> 4. The urge to move the legs and any accompanying unpleasant sensations during rest or inactivity only occur or are worse in the evening or night than during the day.
>
> 5. The occurrence of the above features are not solely accounted for as symptoms primary to another medical or a behavioral condition (e.g., myalgia, venous stasis, leg edema, arthritis, leg cramps, positional discomfort, habitual foot tapping)."

Diagnostically, RLS is considered either primary, often occurring within families, or secondary, developing in association with other conditions (such as iron deficiency anemia, pregnancy, or end-stage renal disease).

In the United States, RLS is believed to affect more than 10 million adults and an estimated 1.5 million children and adolescents [3]. About one-third of those with RLS symptoms are bothered sufficiently enough to seek medical attention. Epidemiologic studies also show that women are at least 50% more susceptible to RLS than men and RLS is more common in older adults, although it can occur in some people as early as the pre-school years.

The current standard of care involves initial prescription of dopaminergic medications – such as Requip, Mirapex, and Neupro – which initially provide symptomatic relief but often become ineffective over continued usage [4]. Tolerance to these medications is rapid and well-documented [5]; approximately 10% of patients per year become refractory to these medications, and fewer than 20% of patients have sustained benefits lasting 10 years or longer [6]. It is also now understood that dopaminergic medications cause what is known as “augmentation”, or paradoxical progressive worsening of RLS symptoms that is much faster than the natural progression of the condition. Due to augmentation, patients on dopaminergic medications require increasingly higher doses [7]. Maximal dosage is limited by an increasing risk of side-effects at higher doses, which include compulsive behaviors including substance abuse, hypersexuality, and gambling [8]. As a result of these downsides of dopaminergic agents, a minority of clinicians are starting to prescribe gabapentinioids (e.g., Horizant) as an alternative first-line of treatment; these medications do not typically lead to augmentation but confer risks such as respiratory depression [9], dizziness, and somnolence during the day.

For the large subpopulation of patients who become refractory to dopaminergic medications – typically due to augmentation – there are limited treatment options. As a result of tolerance, augmentation, and dosage limitations, RLS patients often continue to suffer from moderate-severe RLS symptoms while continuing to be reliant on high doses of dopaminergic medications to provide a small degree of relief. To address the massive unmet need, the leading clinicians involved with RLS advocate prescribing off-label opioids [10]. The leading options, oxycodone and methadone, have well documented risks, which include addiction, dependence, overdose, and occasionally death. This situation is especially concerning because primary RLS typically starts in middle age or earlier and persists throughout life; thus, patients may end up reliant on opioids for the final decades of their lives.

Recently, a new option for the treatment of RLS has become available. The NTX100 Tonic Motor Activation (TOMAC) System was cleared by the FDA in April 2023 and is intended to reduce the symptoms of primary moderate-severe Restless Legs Syndrome and to improve sleep quality in adults refractory to medications. This device is the only FDA-cleared intervention to treat adults who are refractory to RLS medications and suffer from primary moderate-severe RLS.

2.0 STUDY OBJECTIVE

This non-significant risk study is being performed to assess the long-term effectiveness and safety of the NTX100 TOMAC System in a real-world population.

3.0 STUDY DESIGN

3.1 Overview

The study will be a post-market observational study to assess the long-term effectiveness and safety of the NTX100 TOMAC System. There will be a total of 5 study visits in addition to standard of care. Subjects must agree to comply with the study-specific requirements during the follow-up period of 365 days. A maximum of 325 subjects are to be enrolled, at up to 20 clinical sites within the United States.

The schedule of assessments for this study is presented in Table 1.

Table 1: Schedule of Assessments

DATA	**Eval 1 Study Entry Screening** *Remote*	**Titration Visit* Day 0**** *In-office*	**Eval 2 Day 90** *Remote*	**Eval 3 Day 180** *Remote*	**Eval 4 Day 270** *Remote*	**Eval 5 Day 365** *Remote*	Biweekly Texts (26 texts)	**Eval 6 Day 455** *Remote*	**Eval 7 Day 545** *Remote*	**Eval 8 Day 635** *Remote*	**Eval 9 Day 730** *Remote*	Monthly Texts (12 texts)
Screening	x											
Informed Consent	x											
Inclusion/Exclusion	x											
Med Hx/Demographics	x											
ConMeds (Initial and changes)	x		x	x	x	x	x	x	x	x	x	x
RLS History	x											
Refractory categorization	x											
Device titration		x										
AEs			x	x	x	x	x	x	x	x	x	x
Pivotal Trial Matched	x											
IITT Population Questionnaire			x									
Iron Panel Testing Results			x									
Follow-up Questionnaire		x	x	x	x	x		x	x	x	x	
CGI-I			x	x	x	x		x	x	x	x	
PGI-I			x	x	x	x		x	x	x	x	
IRLS	x		x	x	x	x		x	x	x	x	
MOS-SLEEP	x		x	x	x	x		x	x	x	x	
Augmentation Severity Rating Scale	x					x						
EQ-5D-5L	x			x		x					x	
Insomnia Severity Index (ISI)	x			x		x						
Epworth Sleepiness Scale (ESS)	x			x		x					x	
GAD-7	x		x	x	x	x					x	
PHQ-8	x		x	x	x	x					x	
Cognitive Function - Abilities – Short Form 8a	x		x	x		x					x	
Device usage evaluation			x	x	x	x					x	
CPAP User Questionnaire	x					x					x	
Clinical Marketing Questions				x		x					x	

** All patients prescribed the NTX100 complete a titration visit (not just study subjects).*
*** Repeat baseline assessments if titration is scheduled more than 14 days after Eval 1.*

3.2 Method of Assigning Subjects to Treatment

Not applicable.

3.3 Blinding

No blinding will be performed. All subjects are to receive NTX100 treatment.

3.4 Determination of Sample Size

Sample size calculations were performed for the primary effectiveness endpoint, change from baseline in IRLS total score at one year, based on a one-sample t-test at the one-sided alpha = 0.0125 level (Bonferroni-adjusted to account for the two co-primary analysis populations). The "true" mean and standard deviation (SD) were assumed to be the same as the observed mean (SD) change from baseline in IRLS total score at the end of the pivotal CT-04 trial (the RESTFUL study), or -8.7 (6.3). Based on these assumptions with 90% power, the required sample size is only N=10 participants. A total of up to N=325 participants will be enrolled to increase the precision of estimates for the primary and secondary effectiveness endpoints. For example, the two-sided 97.5% confidence interval (CI) for the true mean for the primary effectiveness endpoint with N=325 participants and an observed SD of 6.3 would have a half-width of 0.79.

3.5 Changes to the Protocol-Specified Analyses

There were no changes to the analyses specified in the protocol.

3.6 Changes from Previous Version of the SAP

There were no substantive changes to the analyses specified in the previous SAP.

Section 6.1 was updated to counteract changes to the Protocol-Specified eligibility criteria between Protocol v4.0 and v6.0. Protocol v6.0 expanded the eligibility criteria to allow enrollment of patients who were contraindicated for NTX100 treatment ('contraindicated patients'). To maintain a consistent IITT population definition, Section 6.1 was modified to clarify that those contraindicated patients would continue to be excluded from the IITT population.

Repeat analyses and analysis populations were added to account for additions to the protocol (v4.0 and later) which allow participants to reconsent to extended follow-up time.

4.0 EFFECTIVENESS AND SAFETY ENDPOINTS

4.1 Primary Effectiveness Endpoint

The primary effectiveness endpoint is the change from baseline (study entry) in IRLS total score at 1-year. The IRLS total score change at subsequent 1-year intervals (e.g., 2-years, 3-years, etc.) will also be analyzed as a secondary effectiveness endpoint.

The IRLS total score change will be the first endpoint considered in hierarchical testing at 1-year (the primary endpoint) and for each subsequent timepoint.

4.2 Key Secondary Effectiveness Endpoints

The key secondary effectiveness endpoints are as follows:

Endpoint 1: CGI-I score at 1-year intervals relative to baseline.

Endpoint 2: PGI-I score at 1-year intervals relative to baseline.

Endpoint 3: Change from baseline in MOS-II total score at 1-year intervals.

Endpoint 4: Change from baseline in frequency of RLS symptoms (number of days per week) at 1-year intervals.

The key secondary effectiveness endpoints will also be included in hierarchical testing at each relevant timepoint. The IRLS total score change will be considered first at each 1-year interval, followed by each of the key secondary endpoints in the order listed above.

4.3 Additional Secondary Effectiveness Endpoints

Additional secondary effectiveness endpoints are as follows:

- CGI-I responder, defined as a CGI-I response of "Very Much Improved" or "Much Improved", at 1-year intervals.
- CGI improvement, defined as a CGI-I response of "Very Much Improved", "Much Improved", or "Minimally Improved", at 1-year intervals.

4.4 Exploratory Effectiveness Endpoints

The exploratory effectiveness endpoints are as follows:

- EQ-5D (Quality of Life Questionnaire)
- ISI (Insomnia Severity Index)
- ESS (Epworth Sleepiness Scale).

4.5. Safety Outcomes

Adverse events, classified and tabulated by seriousness, severity, relationship to the device, timing, and severity.

5.0 STATISTICAL CONSIDERATIONS

5.1 General Methodology

The statistical analysis of the data obtained from this study will be performed using SAS® version 9.4 or higher.

Data collected in this study will be documented using summary tables and by-subject data listings. Continuous variables will be summarized using descriptive statistics, specifically the number of subjects, mean, median, standard deviation, minimum and maximum. Categorical variables will be summarized using frequencies and percentages for each category. For continuous data, the minimum and the maximum will use the same decimal place accuracy as the raw data. The mean, median, and standard deviation will use one more decimal place than the raw data. For categorical data, percentages will be reported to one decimal place. P-values will be reported to 4 decimal places. p-values less than 0.0001 will be displayed as <0.0001 in the tables.

A 0.0125 significance level will be used for the hypothesis tests for the primary effectiveness endpoint and the key secondary effectiveness endpoints. The overall one-sided 0.025 significance level was adjusted based upon the Bonferroni method (0.025/2 = 0.0125) to account for the two co-primary analysis populations (Indicated Intent-to-Treat Population and Indicated Stable Medication Population). Within each of these populations, endpoints will be tested annually. Starting at 1-year post-baseline, the primary and key secondary effectiveness endpoints will be tested hierarchically within each co-primary population (primary effectiveness endpoint followed by key secondary effectiveness endpoints 1-4 in order by year), stopping at the first non-statistically significant result. No hypothesis testing will be performed for the Pivotal Trial Matched Population or the Medication-naive Population.

Data listings will be sorted by site number and subject number.

5.2 Adjustments for Covariates

A mixed model repeated measures (MMRM) model including the endpoint value at baseline, visit, and their interaction as predictors (covariates) will be fit for the primary effectiveness endpoint and for key secondary effectiveness endpoints 3 and 4. A similar MMRM model will be fit for key secondary effectiveness endpoints 1 and 2 with visit as the lone predictor variable.

5.3 Handling of Dropouts and Missing Data

Missing data for the primary effectiveness endpoint and all secondary effectiveness endpoints will be handled using multiple imputation (MI) methods. Using SAS® PROC MI, the Markov Chain Monte Carlo (MCMC) method of imputation will be used to create a monotone missing data pattern (only if the missing data pattern is non-monotone). After ensuring a monotone missing pattern, the linear regression and logistic regression methods of imputation will be used to impute the remaining missing data for continuous and categorical endpoints, respectively. For the primary effectiveness endpoint and key secondary effectiveness endpoints 3 and 4, the imputation model will include the following variables in the specified order: the endpoint values at baseline, 90 days, 180 days, 270 days, and 1-year intervals (as applicable). For key secondary effectiveness endpoints 1 and 2 and for the additional secondary effectiveness endpoints, the imputation model will include the following variables in the specified order: the endpoint values at 90 days, 180 days, 270 days, and 1-year intervals (as applicable). The imputation will be

repeated and 50 complete datasets will be created. For each imputed dataset, the mean for the given endpoint and the corresponding standard error will be calculated. PROC MIANALYZE of SAS® will be used to combine the results from the imputed datasets to produce an overall estimate of the true mean and the corresponding 95% confidence interval and to conduct a test of the relevant null hypotheses specified in Sections 10.1 and 10.2.

For each primary and key secondary effectiveness endpoint, a mixed model repeated measures (MMRM) analysis will also be conducted as a secondary analysis based on observed data to evaluate the primary and key secondary effectiveness endpoints across all timepoints overall through one year (or more as applicable) of follow-up. The MMRM will include the endpoint value at baseline, visit, and their interaction as predictors for the primary effectiveness endpoint and for key secondary effectiveness endpoints 3 and 4. The MMRM will include visit as a predictor for key secondary effectiveness endpoints 1 and 2. An unstructured covariance matrix will be used to model the correlation between results for a given subject.

Imputation analyses at 1-year will be conducted as described above. Repeat imputation analyses to address extended follow-up will further include all relevant timepoints from 1-year post-baseline on.

Missing data will be imputed as described above for all effectiveness analyses, except analyses of the Per Protocol (PP) Population and MMRM analyses, which will be based on observed data only. There will be no other imputation of missing data.

5.4 Interim Analyses

The pre-specified effectiveness analysis populations are defined based on expected characteristics of the real-world treated patient population. However, these populations may differ from expectations based on the decisions of clinicians and patients. To account for this, we plan to perform interim analyses to determine if the pre-specified definitions need to be adjusted or if additional populations need to be specified.

5.4.1 First Interim Analysis

Timing: The first interim analysis will be conducted following enrollment of the first 100 patients.

Planned decision 1: If fewer than 75 of the first 100 enrolled patients are included in the Indicated Intent-to-Treat (IITT) Population, then the following IITT criteria will be changed in the following order and the following manner until at least 75 of the first 100 enrolled patients are included in the IITT Population:

1. “Subject receives titrated therapy unit within 2-weeks of Eval 1 completion" will be changed to “Subject receives titrated therapy unit within 4-weeks of Eval 1 completion".
2. "No changes to RLS medication or dosage in 30-days prior to Eval 1 completion" will be changed to "No changes to RLS medication or dosage in week prior to Eval 1 completion".
3. “Subject receives titrated therapy unit within 2-weeks of Eval 1 completion" will be removed.

4. "No changes to RLS medication in 30-days prior to Eval 1 completion" will be removed.

Planned decision 2: If enrollment in the Medication-naïve (MN) Population would be increased by more than 20% by changing the minimum IRLS total score criterion from 15 to 11 (the minimum cutoff for moderate RLS), then this change will be made.

5.4.2 Second Interim Analysis

Timing: The second interim analysis will be conducted following completion of the first 100 patients.

Planned decision: If fewer than 40 of the first 100 completed patients were included in the Indicated Stable Medication (ISM) Population, then (1) the ISM population will be removed as an effectiveness analysis population and designated as an exploratory analysis population and (2) the full one-sided alpha of 0.025 will be allocated to the IITT Population.

5.4.3 Data Analyses for Publication

Informal "interim" analyses may occur to support one or more publications. There will be no formal hypothesis testing and there are no plans to alter the study based on these results. As such, there is no substantive risk of bias in this open-label study.

5.5 Multicenter Study

This is a multicenter study. Up to 20 clinical sites within the United States will participate in the study.

5.6 Multiple Comparisons / Multiplicity

The fixed sequence method of statistical testing will be used for the primary and key secondary effectiveness endpoints in order to control the overall type I error rate. Within each of the two co-primary analysis populations, the primary and key secondary effectiveness endpoints will be tested hierarchically (primary effectiveness endpoint followed by key secondary effectiveness endpoints 1-4 in order) at 1-year post-baseline. If one key secondary effectiveness endpoint does not meet statistical significance, formal testing of subsequent key secondary effectiveness endpoints will not be conducted (but nominal p-values will be calculated).

If all analyses at 1-year result in rejection of the null hypothesis, formal analyses will proceed in the same order annually until the first analysis that results in failure to reject the null in the relevant co-primary analysis population.

5.7 Examination of Subgroups

No subgroup analyses are planned.

6.0 ANALYSIS POPULATIONS

6.1 Indicated Intent-to-Treat (IITT) Population

The IITT Population will consist of subjects who meet the FDA indications for use at study entry and are exposed to therapy. Full criteria are as follows:

a. Adults aged 22 or above
b. Confirmed diagnosis of primary RLS based on International RLS Study Group diagnostic criteria
c. Medication-refractory
d. IRLS total score ≥ 15
e. Subject receives titrated TOMAC therapy unit within 2-weeks of Eval 1 completion.
f. Subject is exposed to at least one TOMAC therapy session
g. No changes to RLS medication or dosage in 30-days prior to Eval 1 completion.
h. Subject has none of the FDA contraindications for TOMAC.
    - Diagnosis of epilepsy or other seizure disorder;
    - Active medical device implant anywhere in the body, including but not limited to pacemakers, spinal cord stimulators, deep brain stimulators;
    - Metal implant in the leg at the site of study device electrode application (knee replacements are typically not a contraindication);
    - Known allergy to device materials (or severe previous reaction to medical adhesives or bandages);
    - Cellulitis, open sores, or acute injury at or near the location of device electrode application

The IITT Population will be used as the first of two co-primary analysis populations for all effectiveness endpoints at 1-year. Participants in the IITT Population who consent to additional follow-up will be included in the respective analyses of subsequent years (e.g., IITT analysis at 2-years post-baseline will include IITT Population participants who consent to additional follow-up through year two).

6.2 Indicated Stable Medication (ISM) Population

The ISM Population will consist of all subjects in the IITT population who also meet the following requirements:

a. No changes to RLS medication or dosage at Day 365 compared to Eval 1.
b. For patients on a stable RLS medication regimen that includes “as needed” doses, no clinically significant changes to the frequency/amount of “as needed” doses. An expert panel blinded to the subject's effectiveness/safety outcomes will evaluate if such changes are clinically significant based on expectation of whether it would be expected to affect any primary or key secondary effectiveness endpoints at Day 365 compared to Eval 1.

The ISM Population will be used as the second of two co-primary analysis populations for all effectiveness endpoints. A safety analysis of the ISM Population will also be conducted. Participants in the ISM Population who consent to additional follow-up will be included in the

respective analyses of subsequent years (e.g., ISM analysis at 2-years post-baseline will include ISM Population participants who consent to additional follow-up through year two).

6.3 Pivotal Trial Matched (PTM) Population

The PTM Population will consist of all subjects in the IITT population who also meet all eligibility criteria for enrollment to the RESTFUL Study (NCT04874155). This is an exploratory effectiveness analysis population for all effectiveness endpoints.

6.4 Medication-naïve (MN) Population

All enrolled patients who meet the following requirements will be included in the MN population:

a. Adults aged 22 or above.
b. Confirmed diagnosis of primary RLS based on International RLS Study Group diagnostic criteria
c. No prior treatment with prescription RLS medication at time of study entry.
d. IRLS total score ≥ 15.
e. Subject receives titrated TOMAC therapy within 2-weeks of Eval 1 completion.
f. Subject is exposed to at least one TOMAC therapy session.

This is also an exploratory effectiveness analysis population.

6.5 Primary Safety Analysis Population

The Primary Safety Analysis Population will consist of all subjects in the IITT Population. The Primary Safety Analysis Population will be used as the primary analysis population for all safety analyses.

6.6 Secondary Safety Analysis Population

The Secondary Safety Analysis Population will consist of all patients who are exposed to therapy (including titration). The Secondary Safety Analysis Population will be used for a secondary analysis of safety.

6.7 Per Protocol (PP) Population

The PP Population will consist of all subjects in the IITT population who completed the study through 1-year, had at least 1 session of TOMAC therapy during each month, and have evaluable data for at least one of the primary or key secondary effectiveness endpoints without any protocol violations that would affect any of these endpoints. The PP Population is an exploratory effectiveness analysis population for all effectiveness endpoints.

7.0 SUBJECT DISPOSITION

The number of enrolled participants will be presented. The number and percentage of enrolled participants who are in the IITT Population, the ISM Population, the PTM Population, the MN Population, the PP Population, the Primary Safety Analysis Population, and the Secondary Safety Analysis Population, who completed the main study as per protocol, who did not complete the main study as per protocol, and, for subjects who did not complete the study as per protocol, the reason for not doing so will be summarized. The numbers and percentages of participants entering and completing extended follow-up will each be summarized annually. Whether or not the subject exited the study with unresolved adverse events will be summarized using frequencies and percentages.

8.0 DEMOGRAPHICS AND BASELINE CHARACTERISTICS

Demographics will be summarized using descriptive statistics for the continuous variable of age and frequencies and percentages for the categorical variables of sex (at birth), ethnicity, and race.

RLS history, medical history, and refractory categorization will be summarized using frequencies and percentages. RLS severity and the Augmentation Severity Rating Scale (ASRS) will be summarized using descriptive statistics for continuous variables and frequencies and percentages for categorical variables.

All of these analyses will be based on the IITT Population.

9.0 PROTOCOL DEVIATIONS

The number and percentage of subjects with any protocol deviations will be presented. The number and percentage of protocol deviations for which a protocol waiver was granted by the Sponsor will be presented. For each deviation type, the number and percentage of protocol deviations associated with that deviation type will be presented. For each deviation reason, the number and percentage of protocol deviations with that reason for the deviation will be presented. The numbers and percentages of clinically significant and not clinically significant protocol deviations will be presented. These results will be presented for the IITT Population.

10.0 EFFECTIVENESS AND SAFETY ANALYSES

10.1 Primary Effectiveness Endpoint Analysis

The primary effectiveness endpoint, the change from baseline (study entry) in IRLS total score at 1-year, will be summarized using descriptive statistics, together with a 95% confidence interval for the true mean based on the t-distribution. The null and alternative hypotheses are as follows:

H_0: $\mu \geq 0$

vs.

H_1: $\mu < 0$

where μ is the true mean at 1-year. For changes to IRLS total score, negative values indicate improvement to RLS. The null hypothesis will be tested using a one-sided, one-sample t-test based on available data. If there are missing data for this endpoint, it will also be analyzed using multiple imputation methods (as described in Section 5.3) to test the null hypothesis at 1-year given above, and the imputation analysis will be considered to be the primary analysis in hierarchical testing using the fixed sequential approach.

The same hypothesis test will be repeated annually for this endpoint at the new milestone (e.g., 2-years) for participants entering extended follow-up for that period. The multiple imputation method will incorporate additional follow-up time accordingly.

As a secondary analysis, an MMRM model including the endpoint value at baseline, visit, and their interaction as predictors will be fit to observed data for the primary effectiveness endpoint, with an unstructured covariance matrix used to model the correlation between results for a given subject. This MMRM analysis will be performed to estimate the change from baseline in IRLS total score through 1-year and annually (i.e., across all post-baseline timepoints overall), and as a secondary analysis to address missing data.

10.2 Secondary Effectiveness Endpoints Analyses

The key secondary effectiveness endpoints are as follows:

Endpoint 1: CGI-I score at 1-year relative to baseline.

Endpoint 2: PGI-I score at 1-year relative to baseline.

Endpoint 3: Change from baseline in MOS-II total score at 1-year.

Endpoint 4: Change from baseline in frequency of RLS symptoms (number of days per week) at 1-year.

The key secondary effectiveness endpoints will be summarized using descriptive statistics, together with 95% confidence intervals for the true means based on the t-distribution. The null and alternative hypotheses for key secondary effectiveness endpoints 1 and 2 are as follows:

H_0: $\mu \geq 4$

vs.

H_1: $\mu < 4$

where μ is the true mean. For CGI-I and PGI-I scores, 4 represents "No change", and values less than 4 indicate improvement to RLS.

The null and alternative hypotheses for key secondary effectiveness endpoints 3 and 4 are as follows:

H_0: $\mu \geq 0$

vs.

H_1: $\mu < 0$

where μ is the true mean. For these two endpoints, negative values indicate improvements to sleep.

For all four key secondary effectiveness endpoints, the null hypothesis will be tested using a one-sided, one-sample t-test based on available data. Each of these endpoints for which there are missing data will also be analyzed using multiple imputation methods (as described in Section 5.3) to test the null hypotheses at 1-year given above, and the imputation analysis will be considered to be the primary analysis in hierarchical testing.

As a secondary analysis, an MMRM model including either (1) visit only (key secondary effectiveness endpoints 1 and 2) or (2) the endpoint value at baseline, visit, and their interaction (key secondary effectiveness endpoints 3 and 4) as predictors will be fit for the key secondary effectiveness endpoints, with an unstructured covariance matrix used to model the correlation between results for a given subject. This MMRM analysis will be performed to estimate the results for each endpoint through 1-year and annually (i.e., across all post-baseline timepoints overall), and as a secondary analysis to address missing data.

The primary and key secondary effectiveness endpoints will be analyzed first at 1-year and annually thereafter using a fixed sequential method in the order they are listed. Key secondary effectiveness endpoint #1 will only be analyzed if the primary effectiveness endpoint analysis results in rejection of the null hypothesis, and subsequent key secondary effectiveness endpoints will only be analyzed if all prior key secondary effectiveness endpoint analyses resulted in rejection of the null hypothesis (e.g., key secondary effectiveness endpoint #3 would only be tested if the primary analyses of the primary and first two key secondary effectiveness endpoints result in rejection of the null hypothesis). Analyses of subsequent key secondary effectiveness endpoints will continue hierarchically until the first analysis that results in failure to reject the null hypothesis, and some or all of the key secondary effectiveness endpoint analyses may not be formally performed.

If all analyses at 1-year result in rejection of the null hypothesis, formal analyses will proceed in the same order annually until the first analysis that results in failure to reject the null in each relevant co-primary analysis population.

The additional secondary effectiveness endpoints, CGI-I responder and CGI-I improvement, will each be summarized using the frequency and percentage of successes, together with a 95% exact

(Clopper-Pearson) confidence interval for the true percentage of successes. These endpoints will also be summarized using multiple imputation methods as described in Section 5.3. No hypothesis testing will be conducted.

10.3 Exploratory Effectiveness Endpoints Analyses

The exploratory effectiveness endpoints are as follows:

- EQ-5D (Quality of Life Questionnaire)
- ISI (Insomnia Severity Index)
- ESS (Epworth Sleepiness Scale)

The exploratory effectiveness endpoints will be summarized using descriptive statistics, together with 95% CIs for the true means based on the t-distribution. No hypothesis testing will be conducted.

10.4 Safety Analyses

All of the safety analyses will be conducted based on the Primary and Secondary Safety Analysis Populations, and the ISM Population.

10.4.1 Adverse Events (AEs)

The number and percentage of subjects with at least one adverse event, at least one serious adverse event, at least one severe adverse event, and at least one device-related adverse event will be presented.

Adverse events will be coded using the Medical Dictionary for Regulatory Activities (MedDRA). AEs will be summarized at the subject level using counts and percentages by MedDRA system organ class (SOC) and preferred term (PT). AEs will also be summarized at the event level using counts and percentages by SOC/PT and seriousness, by SOC/PT and severity, by SOC/PT and relationship to the device, and by SOC/PT and time period (Days 0-90, 91-180, 181-270, and 271-365; yearly for Days 366+).

10.4.2 RLS Medication Changes

The following statistics will be presented for the IITT Population:

- Number and percentage of subjects with an increase to dosage of RLS medication at Day 365 compared to Eval 1. This does not include subjects who switched prescription RLS medications or subjects who changed the frequency/amount of as needed doses.
- Number and percentage of subjects with a decrease to dosage of RLS medication at Day 365 compared to Eval 1. This does not include subjects who switched prescription RLS medications or subjects who changed the frequency/amount of as needed doses.

10.4.3 Concomitant Medications

The World Health Organization Drug Dictionary (WHODRUG) will be used to classify concomitant medications by therapeutic class and preferred term (PT). The number and

percentage of subjects receiving concomitant medications will be summarized by Anatomical Therapeutic Chemical (ATC) level 3 and preferred term. Subjects will be counted only once for an ATC class, even if the subject took the same medication on multiple occasions or more than one medication in the same ATC class. Similarly, subjects will be counted at most once for a PT, even if they took the same medication on multiple occasions or more than one medication with the same preferred term.

11.0 OTHER ANALYSES

CGI-I score, PGI-I score, total IRLS score, MOS-II total score, GAD-7 anxiety total score, and PHQ-8 total score will each be summarized by visit using descriptive statistics.

CPAP users' data and bi-weekly texts data, will be summarized by visit using descriptive statistics for continuous variables and frequencies and percentages for categorical variables.

Clinical marketing questionnaire data will be summarized by visit using frequencies and percentages.

Device malfunctions data will be summarized using frequencies and percentages.

At Day 365 (and annually thereafter), the number of active users will be calculated based on objective usage data from Left and Right leg device logs. An active user will be defined as a study participant in the IITT Population with at least 1 completed NTX100 session for either leg during the previous 30-day period (Days 336-365). Participants with incomplete data for both legs during the 30-day period will not be included in the analysis. The percentage of active users will also be calculated as a percentage of the number of participants in the IITT Population. Statistics will also be presented for Left leg and Right leg separately.

At Day 365 (and annually thereafter), objective usage data from Left and Right leg device logs will be used to calculate the number of months of device usage for each participant during the first 12 30-day periods since Day 0 (Days 1-360). A month of use will be defined as at least 1 completed NTX100 session during the 30-day period. Data will be presented separately for each leg (Right and Left). The number of months of incomplete data will also be summarized for each leg; for example, incomplete data could occur if a device is damaged.

All of these analyses will be conducted based on the IITT Population (including subsets based on consent to extended follow-up periods, as relevant).

12.0 REFERENCES

1. Allen, R.P. et al. Restless legs syndrome: diagnostic criteria, special considerations, and epidemiology. A report from the restless legs syndrome diagnosis and epidemiology workshop at the National Institutes of Health. *Sleep Med*. (2003). doi: 10.1016/s1389-9457(03)00010-8
2. 2012 Revised IRLSSG Diagnostic Criteria for RLS. Available at: http://irlssg.org/diagnostic-criteria
3. Allen, R.P. *et al.* Restless legs syndrome prevalence and impact: REST general population study. *Arch. Intern. Med.* (2005). doi:10.1001/archinte.165.11.1286
4. Silber, M.H. Treatment of restless legs syndrome and periodic limb movement disorder in adults. In: UpToDate, Post, TW (Ed), UpToDate, Waltham, MA, 2020
5. García-Borreguero, D. & Williams, A.M. Dopaminergic augmentation of restless legs syndrome. *Sleep Medicine Reviews* (2010). doi:10.1016/j.smrv.2009.11.006
6. Lipford, M.C. and Silber, M.H. Long-term use of pramipexole in the management of restless legs syndrome. *Sleep Med*. 2012; 13: 1280–1285
7. Hening, W.A. et al. An update on the dopaminergic treatment of restless legs syndrome and periodic limb movement disorder. *Sleep* (2004). doi: 10.1093/sleep/27.3.560
8. Cornelius, J.R, Tippmann-Peikert, M., Slocumb, N.L., Frerichs, C.F., Silber, M.H. Impulse control disorders with the use of dopaminergic agents in restless legs syndrome: a case-control study. *Sleep*. 2010 Jan;33(1):81-87.
9. FDA Drug Safety Communication 12-19-2019. Available at: https://www.fda.gov/drugs/drug-safety-and-availability/fda-warns-about-serious-breathing-problems-seizure-and-nerve-pain-medicines-gabapentin-neurontin.
10. Silber, M. H. *et al.* The appropriate use of opioids in the treatment of refractory restless legs syndrome. *Mayo Clin. Proc.* (2018). doi:10.1016/j.mayocp.2017.11.007

Appendix A: TABLE SHELLS

Table 1.1.1

PRIMARY EFFECTIVENESS ENDPOINT
IITT POPULATION

Variable	Statistic	NTX100 (N=xxx)
Change from baseline (study entry) in IRLS total score at 1 year	n	xxx
	Mean	xx.x
	95% CI for Mean (1)	(xx.x, xx.x)
	Median	xx.x
	SD	xx.x
	Minimum - Maximum	xx - xx
	p-value (2)	x.xxxx
Change from baseline (study entry) in IRLS total score at 1 year (3)	n	xxx
	Mean	xx.x
	95% CI for Mean (1)	(xx.x, xx.x)
	Standard Error	xx.x
	Minimum - Maximum	xx - xx
	p-value	x.xxxx
Change from baseline (study entry) in IRLS total score through 1 year	n	xxx
	Mean	xx.x
	95% CI for Mean (1)	(xx.x, xx.x)
	Median	xx.x
	SD	xx.x
	Minimum - Maximum	xx - xx
	p-value (2)	x.xxxx
	p-value (4)	x.xxxx

Program Name: Creation date, time

Source: Listing 2.3

(1) Confidence interval based on the t-distribution.

(2) p-value based on a one-sided, one-sample t-test of the null hypothesis that the true mean is greater than or equal to 0 versus the alternative hypothesis that it is less than 0. A 0.0125 significance level was used. and the test was conducted on the non-missing data.

(3) p-value based on a one-sided t-test of the null hypothesis that the true mean is greater than or equal to 0 versus the alternative hypothesis that it is less than 0. A 0.0125 significance level was used. All results were based on multiple imputation methods, which were used to impute missing data.

(4) p-value based on a one-sided t-test of the null hypothesis that the true mean is greater than or equal to 0 versus the alternative hypothesis that it is less than 0. A 0.0125 significance level was used. p-value was calculated based on estimates from a mixed model repeated measures (MMRM) analysis.

[Programmer's Note: This table will be repeated for the Indicated Stable Medication Population (Table 1.2.1).]

Table 1.3.1

KEY SECONDARY EFFECTIVENESS ENDPOINTS
IITT POPULATION

Variable	Statistic	NTX100 (N=xxx)
1. CGI-I score at 1 year relative to baseline	n	xxx
	Mean	x.x
	95% CI for Mean (1)	(x.x, x.x)
	Median	x.x
	SD	x.x
	Minimum - Maximum	x - x
	p-value (2)	x.xxxx
1. CGI-I score at 1 year relative to baseline (3)	n	xxx
	Mean	x.x
	95% CI for Mean (1)	(x.x, x.x)
	Standard Error	x.x
	Minimum - Maximum	x - x
	p-value	x.xxxx
2. PGI-I score at 1 year relative to baseline	n	xxx
	Mean	x.x
	95% CI for Mean (1)	(x.x, x.x)
	Median	x.x
	SD	x.x
	Minimum - Maximum	x - x
	p-value (2)	x.xxxx
2. PGI-I score at 1 year relative to baseline (3)	n	xxx
	Mean	x.x
	95% CI for Mean (1)	(x.x, x.x)
	Standard Error	x.x
	Minimum - Maximum	x - x
	p-value	x.xxxx
3. Change from baseline in MOS-II total score at 1 year	n	xxx
	Mean	x.x
	95% CI for Mean (1)	(x.x, x.x)
	Median	x.x
	SD	x.x
	Minimum - Maximum	x - x
	p-value (4)	x.xxxx
3. Change from baseline in MOS-II total score at 1 year (5)	n	xxx
	Mean	x.x
	95% CI for Mean (1)	(x.x, x.x)
	Standard Error	x.x
	Minimum - Maximum	x - x
	p-value	x.xxxx

4. Change from baseline in frequency of RLS symptoms (number of days per week) at 1 year	n	xxx
	Mean	x.x
	95% CI for Mean (1)	(x.x, x.x)
	Median	x.x
	SD	x.x
	Minimum - Maximum	x - x
	p-value (4)	x.xxxx
4. Change from baseline in frequency of RLS symptoms (number of days per week) at 1 year (5)	n	xxx
	Mean	x.x
	95% CI for Mean (1)	(x.x, x.x)
	Standard Error	x.x
	Minimum - Maximum	x - x
	p-value	x.xxxx

Program Name: Creation date, time

Source: Listings 2.1, 2.2, 2.3, and 2.4

Note: The fixed sequence testing method was used for the primary effectiveness and key secondary effectiveness endpoints. Variables 1-4 should be tested in that order. Statistical testing is appropriate for a given variable only if the p-values are statistically significant (p-value ≤ 0.0125) for the previous variables; however, p-values are being presented for all of the variables regardless of the results for previous variables for information purposes only.

(1) Confidence interval based on the t-distribution.

(2) The p-value is from a one-sided, one-sample t-test, testing the null hypothesis that the true mean is greater than or equal to 4 versus the alternative hypothesis that it is less than 4. A 0.0125 significance level was used, and the test was conducted on the non-missing data.

(3) The p-value is from a one-sided t-test, testing the null hypothesis that the true mean is greater than or equal to 4 versus the alternative hypothesis that it is less than 4. A 0.0125 significance level was used. and multiple imputation methods were used to impute missing data.

(4) The p-value is from a one-sided, one-sample t-test, testing the null hypothesis that the true mean is greater than or equal to 0 versus the alternative hypothesis that it is less than 0. A 0.0125 significance level was used, and the test was conducted on the non-missing data.

(5) The p-value is from a one-sided t-test, testing the null hypothesis that the true mean is greater than or equal to 0 versus the alternative hypothesis that it is less than 0. A 0.0125 significance level was used and multiple imputation methods were used to impute missing data.

[Programmer's Note: This table will be repeated for the Indicated Stable Medication Population (Table 1.4.1).]

Table 1.5.1

KEY SECONDARY EFFECTIVENESS ENDPOINTS THROUGH 1 YEAR
IITT POPULATION

Variable	Statistic	NTX100 (N=xxx)
1. CGI-I score through 1 year relative to baseline	n	xxx
	Mean	x.x
	95% CI for Mean (1)	(x.x, x.x)
	Median	x.x
	SD	x.x
	Minimum - Maximum	x - x
	p-value (2)	x.xxxx
2. PGI-I score through 1 year relative to baseline	n	xxx
	Mean	x.x
	95% CI for Mean (1)	(x.x, x.x)
	Median	x.x
	SD	x.x
	Minimum - Maximum	x - x
	p-value (2)	x.xxxx
3. Change from baseline in MOS-II total score through 1 year	n	xxx
	Mean	x.x
	95% CI for Mean (1)	(x.x, x.x)
	Median	x.x
	SD	x.x
	Minimum - Maximum	x - x
	p-value (3)	x.xxxx
4. Change from baseline in frequency of RLS symptoms (number of days per week) through 1 year	n	xxx
	Mean	x.x
	95% CI for Mean (1)	(x.x, x.x)
	Median	x.x
	SD	x.x
	Minimum - Maximum	x - x
	p-value (3)	x.xxxx

Program Name: Creation date, time

Source: Listings 2.1, 2.2, 2.3, and 2.4

(1) Confidence interval based on the t-distribution.

(2) The p-value is from a one-sided, one-sample t-test, testing the null hypothesis that the true mean is greater than or equal to 4 versus the alternative hypothesis that it is less than 4. A 0.0125 significance level was used. Results are based on a mixed model repeated measures (MMRM) analysis.

(3) The p-value is from a one-sided, one-sample t-test, testing the null hypothesis that the true mean is greater than or equal to 0 versus the alternative hypothesis that it is less than 0. A 0.0125 significance level was used. Results are based on a mixed model repeated measures (MMRM) analysis.

[Programmer's Note: This table will be repeated for the Indicated Stable Medication Population (Table 1.6.1).]

Table 1.7.1

ADDITIONAL SECONDARY EFFECTIVENESS ENDPOINTS
IITT POPULATION

Variable	Statistic	NTX100 (N=xxx)
CGI-I Responder at 1 Year		
Yes	n (%)	xx (xx.x)
No	n (%)	xx (xx.x)
	95% CI for % 'Yes' (1)	(xx.x, xx.x)
CGI-I Responder at 1 Year (2)		
Yes	n (%)	xx (xx.x)
No	n (%)	xx (xx.x)
	95% CI for % 'Yes'	(xx.x, xx.x)
CGI-I Improvement at 1 Year		
Yes	n (%)	xx (xx.x)
No	n (%)	xx (xx.x)
	95% CI for % 'Yes' (1)	(xx.x, xx.x)
CGI-I Improvement at 1 Year (2)		
Yes	n (%)	xx (xx.x)
No	n (%)	xx (xx.x)
	95% CI for % 'Yes'	(xx.x, xx.x)

Program Name: Creation date, time

Source: Listing 2.1

Note: CGI-I responder is defined as a CGI-I response of "Very much improved" or "Much improved". CGI-I improvement is defined as a CGI-I response of "Very much improved", "Much improved", or "Minimally Improved".

(1) Exact (Clopper-Pearson) confidence interval for the true percentage of responders.

(2) Results are based on multiple imputation methods.

[Programmer's Note: This table will be repeated for the Indicated Stable Medication Population (Table 1.8).]

Table 2.1.1.1

ADVERSE EVENTS (AEs): OVERVIEW
PRIMARY SAFETY ANALYSIS POPULATION

Parameter	NTX100 (N=xxx) n (%)
At least one AE?	
Yes	xx (xx.x)
No	xx (xx.x)
At least one serious AE?	
Yes	xx (xx.x)
No	xx (xx.x)
At least one severe AE?	
Yes	xx (xx.x)
No	xx (xx.x)
At least one device-related AE? (1)	
Yes	xx (xx.x)
No	xx (xx.x)

Program Name: Creation date, time
Source: Listing 4
(1) AEs that are definitely, probably, or possibly related to the device, or for which the relationship to the device is missing, are considered device-related.

[Programmer's Note: This table will be repeated for the Secondary Safety Analysis Population (Table 2.1.2.1), and the Indicated Stable Medication Population (Table 2.1.3).]

Table 2.2.1.1

ADVERSE EVENTS (AEs): INCIDENCE BY SYSTEM ORGAN CLASS AND PREFERRED TERM (SUBJECT LEVEL)
PRIMARY SAFETY ANALYSIS POPULATION

System Organ Class/Preferred Term	NTX100 (N=xxx) n (%)
System Organ Class 1	xx (xx.x)
Preferred Term 1	xx (xx.x)
Preferred Term 2	xx (xx.x)
...	
System Organ Class 2	xx (xx.x)
Preferred Term 1	xx (xx.x)
Preferred Term 2	xx (xx.x)
...	

Program Name:
Source: Listing 4

Creation date, time

[Programmer's Note: This table will be repeated for the Secondary Safety Analysis Population (Table 2.2.2.1), and the Indicated Stable Medication Population (Table 2.2.3.1).]

Table 2.3.1.1

ADVERSE EVENTS (AEs) BY SYSTEM ORGAN CLASS/PREFERRED TERM AND SERIOUSNESS (EVENT LEVEL)
PRIMARY SAFETY ANALYSIS POPULATION

System Organ Class/Preferred Term	Category 1 n (%)	Category 2 n (%)	Category 3 n (%)	Category 4 n (%)	Category 5 n (%)	Category 6 n (%)	Category 7 n (%)	Category 8 n (%)	Total with Non-Missing Seriousness n (100%)	Missing n
All AEs	xx (xx.x)	xx (xx.x)	xx (xx.x)	xx (xx.x)	xx (xx.x)	xx (xx.x)	xx (xx.x)	xx (xx.x)	xx (100.0)	xx
System Organ Class 1	xx (xx.x)	xx (xx.x)	xx (xx.x)	xx (xx.x)	xx (xx.x)	xx (xx.x)	xx (xx.x)	xx (xx.x)	xx (100.0)	xx
Preferred Term 1	xx (xx.x)	xx (xx.x)	xx (xx.x)	xx (xx.x)	xx (xx.x)	xx (xx.x)	xx (xx.x)	xx (xx.x)	xx (100.0)	xx
Preferred Term 2	xx (xx.x)	xx (xx.x)	xx (xx.x)	xx (xx.x)	xx (xx.x)	xx (xx.x)	xx (xx.x)	xx (xx.x)	xx (100.0)	xx
...										

Program Name: Creation date, time
Source: Listing 4
Note: Category 1: Hospitalization; Category 2: Death; Category 3: Life-threatening; Category 4: Disability or permenent damage; Category 5: Congenital abnormality/birth defect; Category 6: Required intervention to prevent prevent permenent impairment or damage; Category 7: Other Serious (Important Medical events); Category 8: Not a serious AE. Percentages for each category are calculated within each System Organ Class/Preferred Term based on the number of events with non-missing seriousness. The categories are not mutually exclusive, so the percentages may sum to more than 100%.

[Programmer's Note: This table will be repeated for the Secondary Safety Analysis Population (Table 2.3.2.1), and the Indicated Stable Medication Population (Table 2.3.3.1).]

Table 2.4.1.1

ADVERSE EVENTS (AEs) BY SYSTEM ORGAN CLASS/PREFERRED TERM AND SEVERITY (EVENT LEVEL)
PRIMARY SAFETY ANALYSIS POPULATION

System Organ Class/Preferred Term	Mild<br>n (%)	Moderate<br>n (%)	Severe<br>n (%)	Total with Non-Missing Severity<br>n (100%)	Missing<br>n
All AEs	xx (xx.x)	xx (xx.x)	xx (xx.x)	xx (100.0)	xx
System Organ Class 1	xx (xx.x)	xx (xx.x)	xx (xx.x)	xx (100.0)	xx
Preferred Term 1	xx (xx.x)	xx (xx.x)	xx (xx.x)	xx (100.0)	xx
Preferred Term 2	xx (xx.x)	xx (xx.x)	xx (xx.x)	xx (100.0)	xx
...					

Program Name: Creation date, time
Source: Listing 4
Note: Percentages are calculated within each System Organ Class/Preferred Term based on the number of events with non-missing severity.

[Programmer's Note: This table will be repeated for the Secondary Safety Analysis Population (Table 2.4.2.1), and the Indicated Stable Medication Population (Table 2.4.3).]

Table 2.5.1.1

ADVERSE EVENTS (AEs) BY SYSTEM ORGAN CLASS/PREFERRED TERM AND RELATIONSHIP TO THE DEVICE (EVENT LEVEL)
PRIMARY SAFETY ANALYSIS POPULATION

System Organ Class/Preferred Term	Not Related n (%)	Unlikely Related n (%)	Possibly Related n (%)	Probably Related n (%)	Definitely Related n (%)	Total with Non-Missing Relationship n (100%)	Missing n
All AEs	xx (xx.x)	xx (xx.x)	xx (xx.x)	xx (xx.x)	xx (xx.x)	xxx (100.0)	xx
System Organ Class 1	xx (xx.x)	xx (xx.x)	xx (xx.x)	xx (xx.x)	xx (xx.x)	xxx (100.0)	xx
Preferred Term 1	xx (xx.x)	xx (xx.x)	xx (xx.x)	xx (xx.x)	xx (xx.x)	xxx (100.0)	xx
Preferred Term 2	xx (xx.x)	xx (xx.x)	xx (xx.x)	xx (xx.x)	xx (xx.x)	xxx (100.0)	xx
...							

Program Name: Creation date, time
Source: Listing 4
Note: Percentages are calculated within each System Organ Class/Preferred Term based on the number of events with non-missing relationship.

[Programmer's Note: This table will be repeated for the Secondary Safety Analysis Population (Table 2.5.2.1), and the Indicated Stable Medication Population (Table 2.5.3).]

Table 2.6.1.1

ADVERSE EVENTS (AEs) BY SYSTEM ORGAN CLASS/PREFERRED TERM AND TIME PERIOD (EVENT LEVEL)
PRIMARY SAFETY ANALYSIS POPULATION

System Organ Class/Preferred Term	Day 0-90<br>n (%)	Day 91-180<br>n (%)	Day 181-270<br>n (%)	Day 271-365<br>n (%)
All AEs	xx (xx.x)	xx (xx.x)	xx (xx.x)	xx (xx.x)
System Organ Class 1	xx (xx.x)	xx (xx.x)	xx (xx.x)	xx (xx.x)
Preferred Term 1	xx (xx.x)	xx (xx.x)	xx (xx.x)	xx (xx.x)
Preferred Term 2	xx (xx.x)	xx (xx.x)	xx (xx.x)	xx (xx.x)
...				

Program Name: Creation date, time
Source: Listing 4
Note: Percentages are calculated within each System Organ Class/Preferred Term based on the number of events with non-missing time period.

[Programmer's Note: This table will be repeated for the Secondary Safety Analysis Population (Table 2.6.2.1), and the Indicated Stable Medication Population (Table 2.6.3).]

Table 3.1.1.1

CONCOMITANT MEDICATIONS
PRIMARY SAFETY ANALYSIS POPULATION

ATC Classification<br>Preferred Term	NTX100<br>(N=xxx)<br>n (%)
Any Concomitant Medications	xx (xx.x)
ATC Classification 1	xx (xx.x)
Preferred Term 1	xx (xx.x)
Preferred Term 2	xx (xx.x)
...	...
ATC Classification 2	xx (xx.x)
Preferred Term 1	xx (xx.x)
Preferred Term 2	xx (xx.x)
...	...

Program Name: Creation date, time
Source: Listing 3.1

Note: Medication coding was based on the current WHO Drug Dictionary. ATC = Anatomic Therapeutic Class.
Percentages were calculated based on N, the number of subjects in the Primary Safety Analysis Population.
Concomitant medications are medications that were taken after the initiation of study treatment and any medication that started prior to the initiation of study treatment and was ongoing on or after that date.

[Programmer's Note: This table will be repeated for the Secondary Safety Analysis Population (Table 3.1.2.1), and the Indicated Stable Medication Population (Table 3.1.3.1).]

Table 4.1.1.1

RLS MEDICATION CHANGES
PRIMARY SAFETY ANALYSIS POPULATION

Variable	NTX100 (N=xxx) n (%)
Clinically significant increase to prescription RLS medication at Day 365 compared to Eval 1	
Yes	xxx (xx.x)
No	xxx (xx.x)
Clinically significant decrease to prescription RLS medication at Day 365 compared to Eval 1	
Yes	xxx (xx.x)
No	xxx (xx.x)

Program Name: Creation date, time
Source: Listing 3.2

[Programmer's Note: This table will be repeated for the Secondary Safety Analysis Population (Table 4.1.2.1), and the Indicated Stable Medication Population (Table 4.1.3.1).]

Appendix B: LISTING SHELLS

Listing 1.1.1

INFORMED CONSENT AND ANALYSIS POPULATIONS
ENROLLED SUBJECTS

			Member of Analysis Population						
Site Number	Subject Number	Date/Time Informed Consent Signed	IITT Population	Indicated Stable Medication Population	Pivotal Trial Matched Population	Medication-naive Population	Per Protocol Population	Primary Safety Analysis Population	Secondary Safety Analysis Population
xx	xxx	yyyy-mm-dd/hh:mm	xxx	xxx	xxx	xxx	xxx	xxx	xxx

Program Name: Creation date, time

[Programmer's Note: Report Time Informed Consent Signed in 24-hour time.]

Listing 2.1.1

CLINICAL GLOBAL IMPRESSIONS - IMPROVEMENT SCALE (CGI-I)
ENROLLED SUBJECTS

Site Number	Subject Number	Visit Date	Evaluation #	CGI-I Score
xx	xxx	yyyy-mm-dd	x<br>Other, specify	x

Listing 2.2.1

PATIENT GLOBAL IMPRESSIONS - IMPROVEMENT SCALE (PGI-I)
ENROLLED SUBJECTS

Site Number	Subject Number	Visit Date	Evaluation #	PGI-I Score
xx	xxx	yyyy-mm-dd	x<br>Other, specify	x

Listing 2.3.1

INTERNATIONAL RESTLESS LEGS SYNDROME (IRLS)
ENROLLED SUBJECTS

Site Number	Subject Number	Visit Date	Evaluation #	Q1	Q2	Q3	Q4	Q5	Q6	Q7	Q8	Q9	Q10	Total IRLS
xx	xxx	yyyy-mm-dd	x<br>Other, specify	x	x	x	x	x	x	x	x	x	x	xx

Listing 2.4.1

MEDICAL OUTCOMES STUDY SLEEP SCALE (MOS-SLEEP)
ENROLLED SUBJECTS

Site Number	Subject Number	Visit Date	Evaluation #	Q1	Q2 (hrs)	Q3	Q4	Q5	Q6	Q7	Q8	Q9	Q10	Q11	Q12	MOS-I Subscale Score	MOS-II Subscale Score
xx	xxx	yyyy-mm-dd	x<br>Other, specify	x	x	x	x	x	x	x	x	x	x	x	x	xx	xx

Program Name: Creation date, time

Note: For Q1, 1=0-15 minutes, 2=16-30 minutes, 3=31-45 minutes, 4=45-60 minutes, 5=> 60 minutes. For Q3-Q12, 1=All of the time, 2=Most of the time, 3=A good bit of the time, 4=Some of the time, 5=A little of the time, 6=None of the time.

Listing 3.1.1

PRIOR AND CONCOMITANT MEDICATIONS
ENROLLED SUBJECTS
Part 1 of 2

Site Number	Subject Number	Any Concomitant Medications?	Medication Name	Start Date	End Date	Corresponding AE? (#1)	If 'Yes' in #1, AE #	Indication
xx	xxx	xxx	xxxxx	yyyy-mm-dd	yyyy-mm-dd<br>Ongoing	xxx	X	xxxxxx

Listing 3.1.1

PRIOR AND CONCOMITANT MEDICATIONS
ENROLLED SUBJECTS
Part 2 of 2

Site Number	Subject Number	Any Concomitant Medications?	Medication Name	Unit Dose	Frequency	Route
xx	xxx	xxx	xxxxx	xxxxxx	xxxxxx	Xxxxxx

Listing 3.2.1

RLS MEDICATION LOG
ENROLLED SUBJECTS

Site Number	Subject Number	Eval #	Medication	Time	Dose	Route	Started	Ended	Is this a Significant Change in RLS Medication?	Reason for Change	Date of Change
xx	xxx	xx	xxxxx	hh:mm	xxxx	xxxx	yyyy-mm-dd	yyyy-mm-dd<br>Ongoing	xxx	xxxxxxx	yyyy-mm-dd

[Programmer's Note: Report Time as 24-hour time.]

Listing 4.1

ADVERSE EVENTS (AEs)
ENROLLED SUBJECTS
Part 1 of 3

Site Number	Subject Number	AE Number	Event Description	Details	System Organ Class	Preferred Term	Event Start Date	Event End Date	Severity	Relation to the Study Device
xx	xxxx	x	xxxxxxxxxx	xxxxxxxxxx	xxxxxxxxxx	xxxxxxxxxx	yyyy-mm-dd	yyyy-mm-dd<br>Ongoing	xxxxxx	xxxxxxxxxx

Listing 4.1

ADVERSE EVENTS (AEs)
ENROLLED SUBJECTS
Part 2 of 3

Site Number	Subject Number	AE Number	Relation to a Pre-existing Condition (#1)	If ‘Possible’, ‘Probable’, or ‘Definite’ in #1, pre-existing condition	Action Taken	Serious AE (#1)	If 'Yes' in #1, applicable category	Outcome	Notes
xxx	xxxx	x	xxxxxxxxxx	xxxxxxxx	xxxxx<br>Other, describe	xxx	xxxxxx<br>Other Serious, specify	xxxxx<br>Other, describe	xxxxxxx

Listing 4.1

ADVERSE EVENTS (AEs)
ENROLLED SUBJECTS
Part 3 of 3

Site Number	Subject Number	AE Number	Follow-up Timepoint Eval #	Date	AE status
xxx	xxxx	x	x	yyyy-mm-dd	xxxxx

Appendix C: Adjustments for 90-day analysis publication

A publication will be submitted on the 90-day outcomes for all participants. The SAP above will be followed except replacing outcomes at 1 year with outcomes at 90 days. As these outcomes do not contain multiple time points for each metric, the following analysis will be adjusted:

4.4 Exploratory Effectiveness Endpoints

There were no exploratory effectiveness endpoints collected at day 90. Therefore, this analysis will not be completed for the day 90 publication

5.1 General Methodology

Analysis will be performed using R version 4.5 or higher. No analysis will be done in SAS.

5.2 Adjustments for Covariates

We will not adjust for covariates using a mixed model repeated measures (MMRM) analysis as the 90 day results do not contain any repeated measures.

5.3 Handling of Dropouts and Missing Data

Missing data at day 90 will be imputed using multiple imputation (MI) methods. Participants who drop out before day 90 and report outcomes at the time of withdraw will use those outcomes for their day 90 results. For patients with no outcomes other than baseline value, the R MICE library version 3.18.0, the Markov Chain Monte Carlo (MCMC) method of imputation will be used to impute missing Day 90 results. For the primary effectiveness endpoint and key secondary effectiveness endpoints 3 and 4, the imputation model will include the following variables in the specified order: the endpoint values at baseline, whether the participant returned the device or not before day 90, and endpoint values at day 90. For key secondary effectiveness endpoints 1 and 2 and for the additional secondary effectiveness endpoints, the imputation model will include the following variables in the specified order: whether the participant returned the device or not before day 90 and the endpoint value at day 90. The imputation will be repeated, and 100 complete datasets will be created. For each imputed dataset, the mean for the given endpoint and the corresponding standard error will be calculated in R and the pool functions from the MICE library will be used to combine the results from the imputed datasets to produce an overall estimate of the true mean, along with the standard error and the corresponding 95% confidence interval and to conduct a test of the relevant null hypotheses specified in Sections 10.1 and 10.2.

No mixed model repeated measures (MRMM) analysis will be used as the 90 day results do not contain any repeated measures.
