# Supplementary material for: Real-world effectiveness and safety of tonic motor activation therapy for refractory restless legs syndrome: 90-day outcomes from the THRIVE prospective observational study
Source: Sleep. 2026 Jun 3;49(9):zsag140. doi: 10.1093/sleep/zsag140 (PMC13553290; doi:10.1093/sleep/zsag140)
Supplement: Supplementary file 3 [file supplemental_figures_and_tables_thrive_zsag140.pdf]

Real-world effectiveness and safety of tonic motor activation therapy for refractory restless legs syndrome: 90-day outcomes from the THRIVE prospective observational study

Haramandeep Singh*[1]; Mark J. Buchfuhrer*[2]; Joseph Ojile[3]; Asim Roy[4]; Robert Joseph Thomas[5]; Richard K. Bogan[6]; Avinash Aggarwal[7]; Jessica Preciado[8]; Kurtis J. Swartz[8]; Jonathan D. Charlesworth[8]

*These authors have contributed equally to the manuscript

1. iSleep Physicians, San Ramon, CA, USA
2. Stanford University School of Medicine, Stanford, CA, USA
3. Clayton Sleep Institute, LLC, St. Louis, MO, USA
4. Ohio Sleep Medicine Institute, Dublin, OH, USA
5. Department of Medicine, Division of pulmonary, Critical Care & Sleep Medicine, Beth Israel Deaconess Medical Center, Boston, MA, USA
6. Bogan Sleep Consultants, LLC, Columbia, SC, USA
7. University of Pittsburgh Medical Center, Department of Neurology, Pittsburgh, PA, USA
8. Noctrix Health, Inc., Pleasanton, CA, USA

Corresponding Author:

Dr. Jonathan D. Charlesworth
Noctrix Health, Inc.
6700 Koll Center Pkwy, Ste 310

Pleasanton, CA 94566

Phone: (804) 683-4279

jcharlesworth@noctrixhealth.com

Table S1: Medication conversion factors

Medication	Equivalent	Conversion Factor
Pregabalin	Gabapentin	6
Gabapentin	Gabapentin	1
Gabapentin Enacarbil	Gabapentin	0.52
Ropinirole	Pramipexole	0.25
Pramipexole	Pramipexole	1
Rotigotine	Pramipexole	0.25
Methadone	Morphine	4.7
Oxycodone	Morphine	1.5
Belbuca (Buprenorphine film)	Morphine	0.03
Buprenorphine / Naloxone	Morphine	30
Buprenorphine patch	Morphine	12.6
Codeine	Morphine	0.15
Carbidopa / Levodopa	Pramipexole	0.01
Hydrocodone	Morphine	1
Morphine sulfate	Morphine	1
Tramadol	Morphine	0.2

Table S2: MOS Sleep Item-by-Item Analysis

Question #	Category	Mean Day 0	Mean Change	Cohens D	p-value
12	Get sleep needed	74.8	16.3	0.53	<.0001*
4	Feel rested	71.0	15.2	0.52	<.0001*
6	Feel drowsy during day	63.1	-15.7	-0.51	<.0001*
3	Sleep not quiet	61.9	-14.4	-0.47	<.0001*
8	Awaken and trouble falling back asleep	56.9	-14.7	-0.47	<.0001*
1	How long to fall asleep	47.0	-13.5	-0.41	<.0001*
7	Trouble falling asleep	50.7	-12.8	-0.37	<.0001*
9	Trouble staying awake during the day	37.5	-9.0	-0.28	<.0001*
2	Number of hours of sleep	5.9	0.4	0.25	0.0007*
10	Snore	27.8	-7.6	-0.25	0.0009*
5	Short of breath	15.8	-3.7	-0.15	0.0153
11	Take naps during day	32.5	-3.5	-0.10	0.0276

*Significantly different than 0 using 1-sided t-test with Holm-Bonferroni correction

Abbreviations: MOS, Medical Outcomes Study Sleep

Table S3: Adverse events for full patient population

Variable	By Day 90 Visit<br>All (n=325)
AE	
Any	97 (30%)
Device-related	26 (8%)
Serious or Severe AE	
Any	10 (3%)
Device-related	0 (0%)
Discontinuation due to AE	
Any	6 (2%)
Device-related	3 (1%)
MedDRA Preferred Term[a]	
Discomfort, Mild	11 (3%)
Administration site reaction, Mild	6 (2%)
Administration site reaction, Moderate	4 (1%)

[a] MedDRA preferred terms for top 3 most common device related AEs

Abbreviations: AE, adverse event; MedDRA, Medical Dictionary for Regulatory Activities

Table S4: Pearson's Correlation of continuous baseline characteristics and IRLS change

Characteristic	R	p-value	R, correcting for baseline IRLS	p-value, correcting for baseline IRLS
Baseline IRLS	-0.4	<.0001	n/a	n/a
Baseline GAD-7	-0.1	0.13	0.1	0.13
Stimulation Intensity	0	0.97	0.1	0.44
Number of RLS medications ever before enrollment	0	0.76	0	0.55
Years since diagnosis	0	0.92	0	0.67
Serum ferritin (μg/L)	-0.1	0.47	0	0.74
TSAT %	0	0.54	0	0.74
Years on RLS medication	0	0.73	0	0.76
Baseline ISI	-0.3	<.0001	0	0.77
Baseline MOS-II	-0.2	0.0006	0	0.78
Baseline PHQ-8	-0.2	0.0007	0	0.9
Age	0.1	0.45	0	0.95
RLS onset age	0	0.92	0	0.96

Abbreviations: GAD-7, Generalized Anxiety Disorder-7; IRLS, International RLS Study Group Rating Scale; ISI, Insomnia Severity Index; MOS-II, Medical Outcomes Study Sleep Problem Index II; PHQ-8, Patient Health Questionnaire-8; RLS, restless legs syndrome; TSAT, transferrin saturation.

Table S5: Pearson's Correlation of continuous baseline characteristics and MOS-II change

Characteristic	R	p-value	R after correcting for baseline MOS-II	p-value after correcting for baseline MOS-II
Baseline MOS-II	-0.5	<.0001	n/a	n/a
Baseline GAD-7	-0.1	0.38	0.2	0.01
Baseline IRLS	-0.2	0.0044	0.1	0.04
Baseline ISI	-0.4	<.0001	0.1	0.18
Years on RLS medication	0	0.51	-0.1	0.2
Serum ferritin (μg/L)	0	0.56	0.1	0.31
Age	0	0.5	-0.1	0.41
Baseline PHQ-8	-0.3	<.0001	0	0.49
Number of RLS medications ever before enrollment	0	0.57	0	0.51
Years since diagnosis	0	0.66	0	0.53
RLS onset age	0.1	0.29	0	0.71
Stimulation Intensity	0	0.7176	0	0.81
TSAT %	0	0.66	0	0.96

Abbreviations: GAD-7, Generalized Anxiety Disorder-7; IRLS, International RLS Study Group Rating Scale; ISI, Insomnia Severity Index; MOS-II, Medical Outcomes Study Sleep Problem Index II; PHQ-8, Patient Health Questionnaire-8; RLS, restless legs syndrome.

Table S6: Pearson's Correlation of continuous baseline characteristics and CGI-I and PGI-I score

Characteristic	CGI-I R	CGI-I p-value	PGI-I R	PGI-I p-value
Baseline GAD-7	0.12	0.07	0.07	0.34
RLS onset age	0.12	0.07	0.10	0.13
Baseline ISI	0.10	0.14	0.01	0.92
Years on RLS medication	-0.09	0.17	-0.07	0.34
Baseline MOS-II	0.09	0.20	0.00	0.96
Baseline PHQ-8	0.08	0.21	0.02	0.75
Baseline IRLS	0.07	0.30	0.06	0.36
TSAT %	0.07	0.35	-0.02	0.79
Years since diagnosis	-0.05	0.44	-0.03	0.64
Stimulation Intensity	-0.04	0.61	0.01	0.93
Age	0.02	0.73	-0.01	0.88
Serum ferritin (μg/L)	0.00	0.99	0.04	0.58
Number of RLS medications ever before enrollment	0.00	1.00	-0.04	0.59

Abbreviations: CGI-I, Clinical Global Impressions-Improvement; GAD-7, Generalized Anxiety Disorder-7; IRLS, International RLS Study Group Rating Scale; RLS, restless legs syndrome; ISI Insomnia Severity Index; MOS-II, Medical Outcomes Study Sleep Problem Index II; PGI-I, Patient Global Impressions – Improvement; PHQ-8, Patient Health Questionnaire-8; TSAT, transferrin saturation.

Table S7: Two-sided t-test for CGI-I and PGI-I score between categorical baseline characteristics

Characteristic	CGI-I score difference (Patients with – Patients without)	CGI-I p-value	PGI-I score difference (Patients with – Patients without)	PGI-I p-value
Diabetes	-0.53	0.01	-0.30	0.19
AASM Iron infusion Cutoff	0.26	0.10	0.20	0.23
Taking any iron therapy at enrollment	0.41	0.12	0.30	0.28
Familial RLS	-0.21	0.17	-0.07	0.64
RESTFUL Criteria: RLS symptoms not most significant in Lower Legs	-0.25	0.18	-0.30	0.13
Sleep Specialist	-0.19	0.21	0.02	0.89
Using CPAP machine at enrollment	-0.20	0.22	-0.25	0.14
All RESTFUL Criteria	-0.17	0.25	-0.16	0.30
Neuropathy	-0.22	0.26	-0.04	0.86
RESTFUL Criteria: RLS symptoms not most significant around bedtime	-0.18	0.27	-0.18	0.30
Lower Legs	0.39	0.28	0.59	0.12
PTM_Q10	0.34	0.28	0.49	0.15
On SSRI,SNRI, or TCA at enrollment	-0.19	0.28	-0.12	0.52
Alpha-2-delta ligands	-0.15	0.33	-0.21	0.17
RESTFUL Criteria: Stable dose of RLS medication 1 month prior to enrollment	0.21	0.36	-0.01	0.97
Stimulation intensity ≥ 35 mA	-0.14	0.36	0.03	0.84
Female	-0.12	0.42	0.05	0.75
OSA	-0.11	0.48	-0.19	0.24
RESTFUL Criteria: Stable dose of medication dose likely to impact RLS 1 month prior to enrollment	0.21	0.53	-0.02	0.95
Upper Legs	0.12	0.53	0.25	0.20
Opioids	0.08	0.66	0.08	0.65
Other location	0.16	0.67	0.17	0.66
RESTFUL Criteria: RLS symptoms from 10am-6pm are severe	0.10	0.68	0.10	0.70
On antihistamine at enrollment	-0.10	0.7	-0.17	0.53
RESTFUL Criteria: No diagnosis of iron-deficient anemia	0.11	0.70	0.00	1.00
Upper Legs Lower Mild	-0.10	0.70	0.08	0.76
Lower Legs Moderate	0.10	0.70	-0.18	0.49
Dopaminergic Agonists	-0.04	0.79	-0.01	0.97
RESTFUL Criteria: 'Age ≤21 or ≥80'	-0.05	0.88	-0.06	0.86
No RLS Rx	-0.04	0.88	-0.07	0.80
Other RLS Medication	-0.01	0.98	-0.12	0.74
Benzodiazepine	0.01	0.99	0.16	0.70

Abbreviations: AASM, American Academy of Sleep Medicine; CGI-I, Clinical Global Impressions-Improvement; CPAP, Continuous Positive Airway Pressure; GAD-7, Generalized Anxiety Disorder-7; IRLS, International RLS Study Group Rating Scale; RLS, restless legs syndrome; ISI Insomnia Severity Index; MOS-II, Medical Outcomes Study Sleep Problem Index II; OSA, obstructive sleep apnea; PGI-I, Patient Global

Impressions – Improvement; PHQ-8, Patient Health Questionnaire-8; SNRI, Serotonin-norepinephrine Reuptake Inhibitors; SSRI, Selective Serotonin Reuptake Inhibitors (SSRIs); TCA, Tricyclic Antidepressants TSAT, transferrin saturation.

Table S8: New iron or sleep disorder treatments do not explain response to TOMAC

Metric	No new* sleep disorder or iron treatment (n=214)	No new* iron treatment (n=226)	No new* sleep disorder treatment (n=220)	New* IV Iron (n = 5)	New* oral iron (n = 1)	New* CPAP (n = 2)	New* sleep Rx (n = 11)	p value no new* treatments vs new treatments
IRLS total score change from baseline to Day 90, mean (95% CI)	-8.4(-7.3, -9.5)	-8.5 (-7.4, -9.6)	-8.5 (-7.4, -9.6)	-8.3 (-2.6, -14.0)	-11.0 (n/a, n/a)	-7.5 (-0.6, -14.4)	-6.6 (-3.0, -10.2)	0.50
CGI-I score at Day 90, mean (95% CI)	2.4 (2.6, 2.2)	2.4 (2.6, 2.2)	2.3 (2.5, 2.1)	2.8 (3.9, 1.7)	2.0 (n/a, n/a)	2.5 (4.0, 1.0)	2.6 (3.3, 1.9)	0.29
PGI-I score at Day 90, mean (95% CI)	2.6 (2.8, 2.4)	2.6 (2.8, 2.4)	2.6 (2.8, 2.4)	3.1 (4.3, 1.9)	2.0 (n/a, n/a)	3.0 (4.6, 1.4)	2.4 (3.1, 1.7)	0.92
MOS-II score change from baseline to Day 90, mean (95% CI)	-12.9 (-10.3, -15.5)	-12.5 (-9.9, -15.1)	-12.5 (-9.9, -15.1)	-11.1 (3.3, -25.5)	-10.6 (n/a, n/a)	-1.4 (4.6, -7.4)	-19.6 (-8.7, -30.5)	0.92
Days per week with RLS symptoms change from baseline to Day 90, mean (95% CI)	-1.3 (-1.0, -1.6)	-1.4 (-1.1, -1.7)	-1.4 (-1.1, -1.7)	-1.0 (1.0, -3.0)	-2.0 (n/a, n/a)	-0.5 (0.5, -1.5)	-0.7 (1.0, -2.4)	0.42

Abbreviations: IRLS, International RLS Study Group Rating Scale; IV, intravenous; MOS, Medical Outcomes Study Sleep; RLS, Restless Legs Syndrome

*Treatments were considered “new” if they were started at any time from 90 days prior to enrollment through the Day 90 endpoint.

Figure S1: Changes in RLS severity and dose aligned to date of change for dopamine agonist reducers

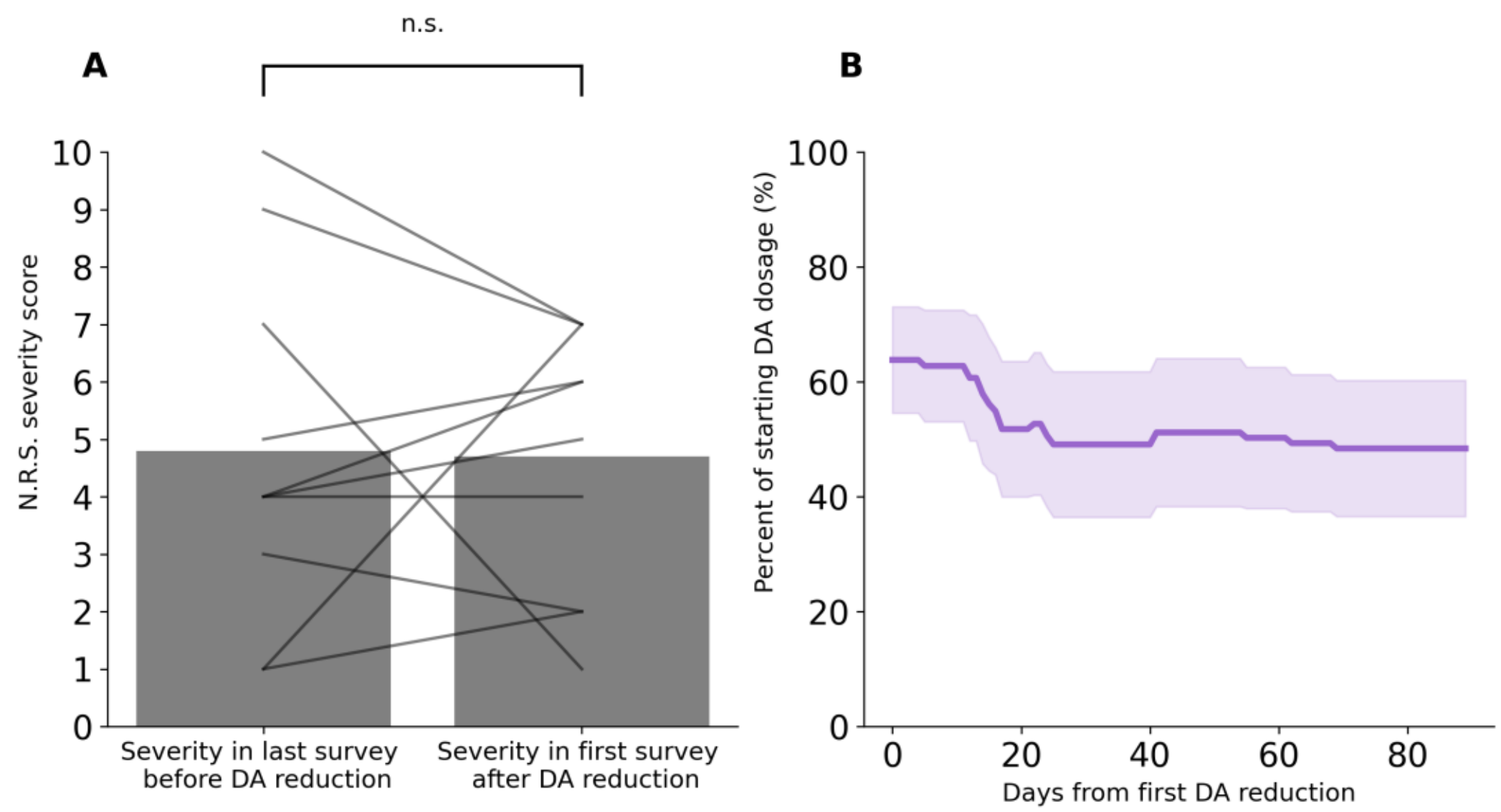

Figure S1 Legend: (A) Patient responses to biweekly questionnaire on overall RLS severity in the last survey taken before the first dopamine reduction to the first survey taken at least 1 day and no more than 14 days after the first dopamine reduction. **(B)** Patient averaged percentage reduction in DA dose (PPX MME) aligned to first reduction (Day 0). Abbreviations: DA, dopamine agonist; RLS, restless legs syndrome

Figure S2: Additional subgroup comparisons in IRLS and MOS-II change

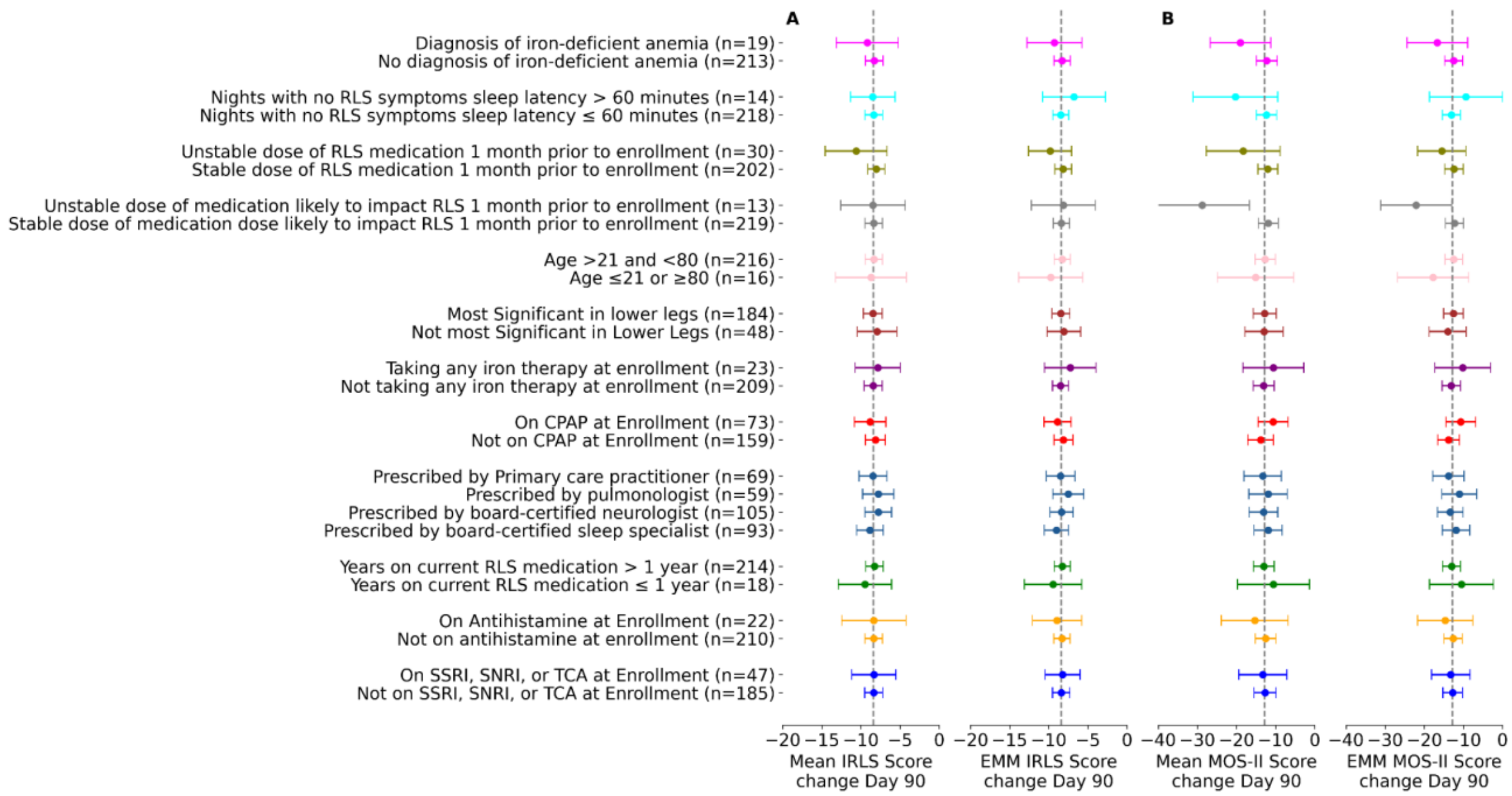

Figure S2 Legend: Mean change and 95% confidence intervals in **(A)** IRLS score and **(B)** MOS-II score at day 90 across different subgroups. Left panels show the mean change before correcting for baseline values. Right panels show the estimated marginal means of each subgroup correcting for either baseline **(A)** IRLS score or **(B)** MOS-II score. Dotted line shows overall average for IITT population. IRLS, International RLS Study Group Rating Scale; MOS-II, Medical Outcomes Study Sleep Problem Index II; IITT, indicated intent to treat; EMM, estimated marginal mean; SSRI, Selective Serotonin Reuptake Inhibitors (SSRIs); SNRI, Serotonin-norepinephrine Reuptake Inhibitors; TCA, Tricyclic Antidepressants
